# Genome-wide SNP profiling of worldwide goat populations reveals strong partitioning of diversity and highlights post-domestication migration routes

**DOI:** 10.1186/s12711-018-0422-x

**Published:** 2018-11-19

**Authors:** Licia Colli, Marco Milanesi, Andrea Talenti, Francesca Bertolini, Minhui Chen, Alessandra Crisà, Kevin Gerard Daly, Marcello Del Corvo, Bernt Guldbrandtsen, Johannes A. Lenstra, Benjamin D. Rosen, Elia Vajana, Gennaro Catillo, Stéphane Joost, Ezequiel Luis Nicolazzi, Estelle Rochat, Max F. Rothschild, Bertrand Servin, Tad S. Sonstegard, Roberto Steri, Curtis P. Van Tassell, Paolo Ajmone-Marsan, Paola Crepaldi, Alessandra Stella, the AdaptMap Consortium

**Affiliations:** 1 DIANA Dipartimento di Scienze Animali, della Nutrizione e degli Alimenti, Università Cattolica del S. Cuore, Piacenza, Italy; 2 BioDNA Centro di Ricerca sulla Biodiversità e sul DNA Antico, Università Cattolica del S. Cuore, Piacenza, Italy; 3 School of Veterinary Medicine, Department of Support, Production and Animal Health, São Paulo State University (UNESP), Araçatuba, Brazil; 4 Dipartimento di Medicina Veterinaria, University of Milan, Milan, Italy; 5 Department of Animal Science, Iowa State University, Ames, IA USA; 6 Department of Molecular Biology and Genetics, Center for Quantitative Genetics and Genomics, Aarhus University, Århus, Denmark; 7 Consiglio per la Ricerca in Agricoltura e l’Analisi dell’Economia Agraria (CREA) - Research Centre for Animal Production and Aquaculture, Monterotondo, Rome, Italy; 8 Population Genetics Lab, Smurfit Institute of Genetics, Trinity College of Dublin, Dublin, Ireland; 9 Faculty of Veterinary Medicine, Utrecht University, Utrecht, Netherlands; 10 Animal Genomics and Improvement Laboratory, Agricultural Research Service, United States Department of Agriculture, Beltsville, MD USA; 11 Present Address: Laboratory of Geographic Information Systems (LASIG), School of Architecture, Civil and Environmental Engineering (ENAC), École Polytechnique Fédérale de Lausanne (EPFL), Lausanne, Switzerland; 12 Fondazione Parco Tecnologico Padano, Lodi, Italy; 13 GenPhySE, INRA, Université de Toulouse, INPT, ENVT, 31326 Castanet Tolosan, France; 14 Recombinetics Inc., Saint Paul, MN USA; 15 National Institute of Aquatic Resources, Technical University of Denmark, DTU, Lyngby, Denmark; 16 Present Address: Center for Genetic Epidemiology, Department of Preventive Medicine, Keck School of Medicine, University of Southern California, Los Angeles, CA USA; 17 Istituto di Biologia e Biotecnologia Agraria, Consiglio Nazionale delle Ricerche, Milan, Italy

## Abstract

**Background:**

Goat populations that are characterized within the AdaptMap project cover a large part of the worldwide distribution of this species and provide the opportunity to assess their diversity at a global scale. We analysed genome-wide 50 K single nucleotide polymorphism (SNP) data from 144 populations to describe the global patterns of molecular variation, compare them to those observed in other livestock species, and identify the drivers that led to the current distribution of goats.

**Results:**

A high degree of genetic variability exists among the goat populations studied. Our results highlight a strong partitioning of molecular diversity between and within continents. Three major gene pools correspond to goats from Europe, Africa and West Asia. Dissection of sub-structures disclosed regional gene pools, which reflect the main post-domestication migration routes. We also identified several exchanges, mainly in African populations, and which often involve admixed and cosmopolitan breeds. Extensive gene flow has taken place within specific areas (e.g., south Europe, Morocco and Mali-Burkina Faso-Nigeria), whereas elsewhere isolation due to geographical barriers (e.g., seas or mountains) or human management has decreased local gene flows.

**Conclusions:**

After domestication in the Fertile Crescent in the early Neolithic era (ca. 12,000 YBP), domestic goats that already carried differentiated gene pools spread to Europe, Africa and Asia. The spread of these populations determined the major genomic background of the continental populations, which currently have a more marked subdivision than that observed in other ruminant livestock species. Subsequently, further diversification occurred at the regional level due to geographical and reproductive isolation, which was accompanied by additional migrations and/or importations, the traces of which are still detectable today. The effects of breed formation were clearly detected, particularly in Central and North Europe. Overall, our results highlight a remarkable diversity that occurs at the global scale and is locally partitioned and often affected by introgression from cosmopolitan breeds. These findings support the importance of long-term preservation of goat diversity, and provide a useful framework for investigating adaptive introgression, directing genetic improvement and choosing breeding targets.

**Electronic supplementary material:**

The online version of this article (10.1186/s12711-018-0422-x) contains supplementary material, which is available to authorized users.

## Background

Goats (*Capra hircus*) are the most important livestock species for poverty alleviation and rural development [1]. More than 90% of goats are farmed in Asia and Africa [1]. On the one hand, their smaller size and lower management requirements compared to cattle means that rearing goats can be afforded also by low-income farmers in remote areas. Many goat breeds can withstand harsh conditions and survive primarily by scavenging for nourishment, and, thus, require little investment for maintenance. On the other hand, when investment capital is available, goats can yield handsome returns [1]. Thus, goats are present in a wide variety of production environments. For these and other reasons, e.g., limited formal crossbreeding and presence of only a few cosmopolitan breeds compared to other livestock species [1], goats are also among the best animals for studying genetic diversity and adaptation.

Goats are among the “big five” livestock species (cattle, sheep, goats, pigs and chickens) recognized by the FAO [1] and are considered as the first to have been domesticated. Domestication occurred around 10,000 years ago (YBP year before present) in Southwest Asia, with at least four distinct domestication events [2], which all involved the bezoar (*Capra aegagrus*) as wild ancestor. Following domestication, goats accompanied humans in their migrations and dispersed throughout the world. As in other domestic species, goats migrated into Europe and arrived at the far north and west edges of the continent by about 5000 YBP [3]. Expansion southwards to Africa and eastwards into Asia occurred at the same time [4]. Goats were present in North Africa around 6000–7000 YBP [5], and in the Sahara and Ethiopia around 5000 YBP [6, 7]. The presence of trypanosomiasis in Central Africa slowed the expansion southwards, and goats reached south sub-Saharan Africa only around 2000 YBP. In Asia, available evidence indicates that goats were present in China around 4500 YBP [8], and that they moved further south and east during the subsequent millennia. Local domestication events in Asia have been hypothesized [9, 10] to support the Asian origin of cashmere goat breeds, but these hypotheses are not supported by recent molecular evidence [2]. Goats reached the Americas and Oceania approximately during the 15th and 18th century of the common era (CE), respectively, along with European migrations [9].

Hence, goats have colonized a wide variety of different geographic and agro-ecological areas around the world. Following colonization, genetic drift, reproductive isolation, together with natural and human-mediated selection led to the development of nearly 600 breeds [1]. These breeds differ by a range of characteristics: body size and weight; hair type and colour; ear shape; horn shape, size and number; milk, meat and fibre production, adaptation to husbandry and environmental conditions. Genetic variation underlies the differences that are observed for these traits. The molecular genetic characterization of breed diversity provides information useful for assessing and mitigating inbreeding, for the proper management of animal genetic resources [11, 12], the reconstruction of breed origin, the investigation of the genetic bases of relevant phenotypes, and the prioritization of breeds for conservation [13]. If biological samples are geo-referenced, molecular data can be integrated with climatic and socio-economic data into geographic information systems (GIS) to allow for a greater range of inferences to be drawn [14, 15].

Previous genetic studies that explored the variation of sheep populations worldwide revealed a low degree of differentiation (2.98% of total variation) between continents and high levels of haplotype sharing [16]. In contrast, cattle populations are highly structured into continental groups that mirror the different ancestry contributions from the gene pools of *Bos taurus* and *Bos indicus*–*Bos javanicus* (13% of variation) and, within taurine cattle, between European and African *B. taurus* (3.2%) [17, 18].

Although several goat breeds have already been characterized genetically [19], most studies have involved only a few breeds analysed in a national context (e.g. [20–22]). Two large-scale investigations based on microsatellite markers highlighted the occurrence of a number of regional gene pools, together with a clinal reduction in variability from the domestication centre in Southwest Asia towards North Europe [23], Indonesia and China [24].

Contrary to the weak population structuring found within mitochondrial DNA (mtDNA) lineages due to the high frequency (> 90%) and worldwide distribution of haplogroup A [25], a clear geographical differentiation between continents was revealed by Y-chromosome haplotype analyses [4, 26, 27]; in addition to the widely distributed haplotype Y1A, haplotypes Y2A and Y1B have been reported in Europe, North Africa and the Near East, whereas haplotype Y2B was found in Asia and haplotype Y2C in Turkey.

A recent study based on ancient DNA (aDNA) data showed that both mitochondrial and nuclear molecular variation of Neolithic goat herds was strongly structured [28]. This evidence supported the hypothesis of multiple wild origins for early domestic goat populations that was already suggested from analyses of modern mtDNA data [2, 25, 29], and further indicated that recruitment from different local bezoar populations was extensive.

In addition, according to nuclear aDNA evidence, early goat populations from the western, eastern and southern regions of the “Near East” contributed differently to the European, African and Asian modern goat populations. Subsequently, post-Neolithic migrations and trade led to an increase in animal movements and exchanges, with resulting admixture and reduction of genetic partitioning [28], which weakened the phylogeographic structure of mtDNA, as testified by the fact that haplogroup A had already become predominant in samples from the Chalcolithic age [7100–5700 before common era (BCE)].

Taken together, the available evidence points at a lack of geographical partitioning of modern maternal lineages, the existence of highly structured variation for the Y-chromosome, nuclear DNA and neolithic mtDNA data, and the occurrence of a number of local gene pools within different geographical regions. However, a comprehensive description of the worldwide distribution of present day goat diversity is still lacking.

The commercial goat 52 K single nucleotide polymorphism (SNP) chip has been available since approximately 5 years [30], and has already been used for national and multi-country goat diversity studies [21, 31, 32]. Using this standardized tool for genotyping, the AdaptMap initiative [33] gathered a dataset that includes genotypes of 148 goat populations.

The aims of this study were to (i) investigate the distribution of goat genetic variation around the world, (ii) compare the present-day diversity patterns to those observed in ancient goat samples and in other ruminant livestock species, and (iii) reconstruct admixture and migration events that have shaped goat post-domestication history.

## Methods

### Construction of the working dataset and quality control

**Fig. 1:**
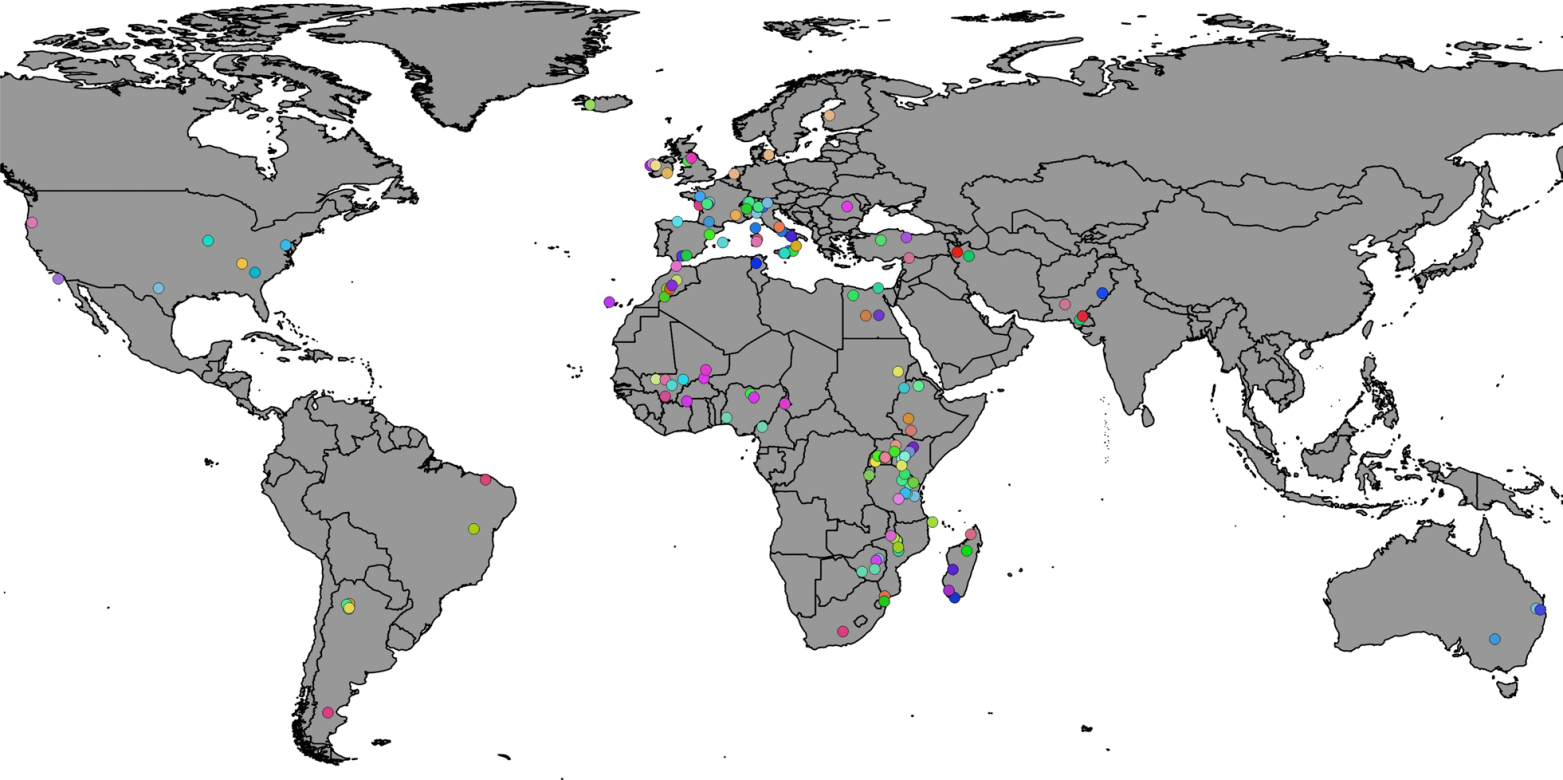
Geographical distribution of the goat breeds included in the AdaptMap project on the world map

The AdaptMap raw dataset includes 4653 animals representing 169 populations from 35 countries across six continents (see Additional file 1: Table S1 and Fig. 1): 46 populations from Europe, 83 from Africa, 21 from West Asia, seven from North America, eight from South America and four from Oceania (for further details on the geographical distribution of breeds see [33]). We used the Illumina GoatSNP50 BeadChip [30] that includes 53,347 SNPs to genotype the animals. Original SNP genomic positions were remapped to the new goat reference sequence ARS1 [34].

We performed data quality control (QC) by using Plink 1.9 [35, 36]. Individuals and SNPs that did not pass the following thresholds were removed: individual genotype call rate higher than 0.96, SNP call rate higher than 0.98, and pairwise identity-by-state between genotypes (based on all markers) less than 0.99. SNPs that were completely monomorphic across the whole dataset were also removed.

To identify individuals with high relatedness (e.g., parent–offspring pairs), we used an in-house script to calculate the number of Mendelian errors (ME) in pairwise comparisons between all individuals [37]. Pairs of animals with less than 100 ME were considered as related [38]. Animals that occurred most frequently within the pairs of related individuals were removed.

Twelve crossbred populations were omitted from the analyses (BOEx, GALxSAA, MTBx, MUBx, OIGx, SAAxANB, SAAxCRE, SEAx, SEAxALP, SEAxGAL, SEAxSAA and SEAxTOG, for the breed code legend see Additional file 1: Table S1), together with five populations with less than three animals sampled (BAG, GSH, MYO, SCL, TET). Breeds for which a large number of samples was collected from multiple locations (ALP, ANG, BOE, LNR, NBN, SAA) were split according to their origin (see Additional file 1: Table S1). The size of the populations with more than 50 sampled individuals (ALP_CH, ALP_IT, ANG_AR, BOE_CH, BRK, BUR, CRE, LNR_DK, NBN_EG, OSS, RAN, SAA_FR, SEA, SID, WAD) was reduced to 50 through a representative random sample selection procedure implemented in the BITE R package [39], which decreases the sample size to a user-defined number while maintaining the variance structure of the original set.

The working version of the AdaptMap dataset (hereafter called “working dataset” or “WD”, 3197 animals, 144 populations and 48,827 SNPs) was used for subsequent analyses. Among the SNPs, 46,666 were mapped on autosomes, 1595 on chromosome X and 566 were unmapped. GenABEL [40] and BITE R packages [39] were used to estimate minor allele frequencies (MAF), SNP and individual call rates.

Phasing and imputing of missing data were carried out on 3197 animals and 46,654 autosomal SNPs with unique positions (hereafter called “phased dataset” or “PD”) by using Beagle v4.0 (r1399) [41].

### Construction of continental datasets

To better explore the geographical partitioning of diversity both between and within the most represented continents, separate datasets were created for Africa (“African dataset” or “AD” = 1183 animals, 56 populations, 48,827 SNPs), Europe (“European dataset” or “ED” = 995 animals, 42 populations, 48,827 SNPs), and west Asia (“West Asian dataset” or “WAD” = 555 animals, 23 populations, 48,827 SNPs), and for the three continents together (“3-continent” dataset or “3CD” = 2729 animals, 121 populations, 48,827 SNPs). Furthermore, for the 3CD and African datasets, individuals were grouped according to the results of the population structure analysis carried out on the working dataset in order to mirror gene pools rather than nominal breeds (see Additional file 2). In particular, the 3CD dataset was compared to WD to evaluate the overall effect of the removal of admixed populations and of gene pool subdivision on the partitioning of diversity.

### Estimation of genetic diversity, gene flow and historic effective population size trends

To estimate within-population diversity and between-population differentiation, we used the software Arlequin version 3.5.2.2 [42] to calculate (i) observed (H_O_) and expected heterozygosity (H_E_), subsequently corrected over the number of usable SNPs, i.e., those having less than 5% missing data, by applying the formulas H_O_corrected_ = H_O_uncorrected_ (number of polymorphic SNPs/number of usable SNPs) and H_E_corrected_ = H_E_uncorrected_ (number of alleles—1)/number of alleles, where number of alleles = 2 (number of individuals in the WD); (ii) the inbreeding coefficient, F_IS_ [43], (iii) Wright’s F_ST_ fixation index [44], and their respective statistical significances.

The composite-likelihood method implemented in Jaatha version 2.7.0 [45, 46] was applied to estimate gene flow as the number of migrants per generation that were exchanged between populations. For the analysis, the following parameter values were set: (i) split time τ, which specifies how many generations ago the populations split, τ is measured in units of 4Ne generations ago, where Ne is the (diploid) effective population size of the first population, and is comprised within the 0.02–5 interval; (ii) scaled migration rate M, given by M = 4Nem, where m is the fraction of individuals of each population replaced by immigrants from the other population at each generation and varies between 0.01 and 25; (iii) mutation parameter θ, which is linked to 4Ne times the neutral mutation rate per locus and ranges from 1 to 20, and (iv) recombination parameter, which is 4Ne times the probability of recombination between the ends of the locus per generation, equal to 20.

Historical trends in effective population size (Ne) were estimated with the SNeP software [47] with default settings and a correction to adjust linkage disequilibrium (LD) (r^2^) values for small sample sizes. Since the large differences in population size within the WD set could negatively affect the estimations of SNeP, we fixed the number of analysed individuals per population to 22. Populations with fewer animals (19 populations from Europe, 44 from Africa, 13 from west Asia, three from North America, two from South America and one from Oceania; see complete list in Additional file 2) were discarded, whereas populations with a sample size larger than 22 were sub-sampled using the aforementioned procedure implemented in the BITE R package [39].

### Analysis of genetic structure

Multi-dimensional scaling (MDS) plots based on kinship matrices were constructed with GenABEL [40] and BITE R packages [39] at the level of both single individuals and populations to investigate the relationships within and between breeds.

Analysis of MOlecular VAriance (AMOVA) [48] was carried out with the Arlequin software on the single continent datasets, and on the WD and 3CD sets to estimate the partitioning of variation within and between continents, and to understand how the presence/absence of admixed populations and gene-pool based grouping impacted the variance distribution. In all analyses, three hierarchical levels were considered, “within individuals”, “among individuals within populations”, and “among populations”. For the 3CD set, we added the “among groups” level corresponding to the subdivision of the populations into continental groups.

To assess possible differences in the LD structure between continents, we used the phased data to calculate the pairwise LD between SNPs with PLINK 1.9 [35, 36] by considering a distance of up to 10 Mbp. For each population, SNP pairs with an r^2^ higher than 0.3 were selected. Then, we calculated the number of populations per continent for which a specific pair of SNPs was in LD.

To assess reticulate relationships between populations, we used Arlequin to calculate the Reynolds unweighted distances, D_R_, between populations [49], which were subsequently visualized via a Neighbour-net graph with the software SplitsTree ver. 4.14.2 [50].

Population structure was assessed by the maximum-likelihood based approach implemented in the Admixture software v1.3.0 [51]. Analyses were run for K values ranging from 2 to 100 under the assumptions of Hardy–Weinberg equilibrium, complete linkage equilibrium and under the ‘unsupervised’ mode (i.e., no prior information on the population of origin of the individuals). To identify the best fitting number of hypothetical populations, for each K value, both fivefold cross-validation error values and the number of iterations needed to reach convergence were considered.

To investigate fine-scale genetic differentiation, we ran Chromopainter and FineStructure software version 2.0.4 [52] on phased data. Chromopainter was run on unlinked mode to calculate the chromosomal chunk donor frequencies using a uniform recombination map, and the coancestry matrix was then computed (using greedy optimization and default settings). FineStructure clustering for each analysis was repeated until independent runs broadly agreed, i.e., until the difference in cluster number (K) reached 5. Convergence was assessed by inspection of the coincidence matrix of the final clustering of the runs.

### Migration events

The occurrence of migration events was evaluated with the Treemix software [53] on the unphased version of the WD set with the Bezoar (BEZ) population set as a root. Windows with 200 consecutive SNPs were used to account for the non-independence of SNPs located in close vicinity. Migrations from *m*0 to *m*11 were tested, with three replicates per *m* to assess consistency. To further assess the consistency of the migration edges found over the WD set, the Treemix software was also run on the 3CD set with the same settings as described above for a number of migrations between *m0* and *m15*, and with three replicates per *m*.

## Results

The overall mean values of observed (H_O_) and expected (H_E_) heterozygosity, were equal to 0.356 and 0.366, respectively, whereas within-population H_O_ ranged from 0.135 (ICL_IS breed from Iceland) to 0.415 (SAA_IT from Italy), and H_E_ from 0.151 (ICL_IS) to 0.425 (CRP_RO) (see Additional file 1: Table S1). Within-continent average heterozygosities calculated over the WD set were for: Africa, H_O_ = 0.354 and H_E_ = 0.360; Oceania, H_O_ = 0.349 and H_E_ = 0.378; Europe: H_O_ = 0.355 and He = 0.379; West Asia, H_O_ = 0.375 and H_E_ = 0.348; North America: H_O_ = 0.317 and H_E_ = 0.399; South America: H_O_ = 0.362 and H_E_ = 0.379.

Eighteen populations had statistically significant F_IS_ values. The only population for which a significant negative value of − 0.086 was obtained was CAN_BR, while the highest F_IS_ (0.251) was found for BEZ_IR, the Iranian Bezoars (see Additional file 1: Table S1 and Additional file 3: Figure S1). Among the 16 other populations with positive significant F_IS_ values, eight were native to Africa (SEA_KE, SEA_MZ, SEA_UG, SID_EG, SOF_MG, WAD_NG, WAD_CM and OSS_EG), three to Europe (LNR_DK, PYR_FR and CCG_IT), two to West Asia (BEZ_IR and BRI_PK), one to South America (CRE_AR), while three were populations belonging to cosmopolitan breeds reared in Tanzania, USA and Argentina (MLY_TZ, MLY_US and SAA_AR).

**Fig. 2:**
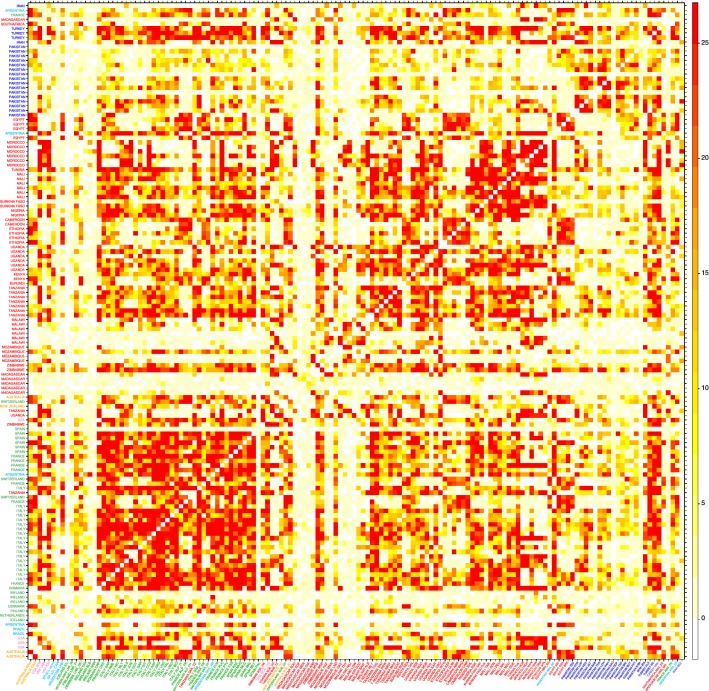
Heatmap-like representation of between-population gene flow. Gene flow was estimated in terms of number of migrants between pairs of populations with the Jaatha software. The corresponding numerical values are in Table S1 (see Additional file 1: Table S1)

Wright’s fixation index F_ST_ ranged from 0.000 (CRO_UG vs. NGD_UG) to 0.556 (ICL_IS vs. MAN_MZ) (see Additional file 4: Table S2 and Additional file 5: Figure S2). Most of the breeds that showed relatively high F_ST_ values in pairwise comparisons were native to islands, such as ICL_IS from Iceland (F_ST_ was always ≥ 0.3), ARR_IE from Ireland, MEN_MG and SOF_MG from Madagascar, PAL_ES from the Canary islands, except for BAB_PK, KAC_PK, KAM_PK and PAT_PK from Pakistan. Most low F_ST_ values were observed within specific geographical areas in Africa, e.g., Morocco and North Africa in general, Uganda–Burundi–Kenya, Tanzania–Ethiopia, or Mali–Burkina Faso–Nigeria–Cameroon, etc. (see Additional file 4: Table S2 and Fig. 2).

Estimates of gene flow obtained with the Jaatha software were visualized via a heatmap (Fig. 2) and the numerical values are in Table S2 (see Additional file 4: Table S2). They show extensive gene flow within well-defined geographical areas, as in the case of Southern Europe, Morocco, Ethiopia-Cameroon, Mali-Burkina Faso-Nigeria and Pakistan. Extensive gene flow among geographical areas involve mostly the Turkish breeds and a few breeds native to Zimbabwe, and also between the populations from South Europe and North Africa. In contrast, a marked lack of gene flow was highlighted for the wild goats from Iran, and the insular populations from Madagascar, Ireland, Iceland and the Canary islands, but also for the breeds from Pakistan, Malawi and Mozambique.

**Fig. 3:**
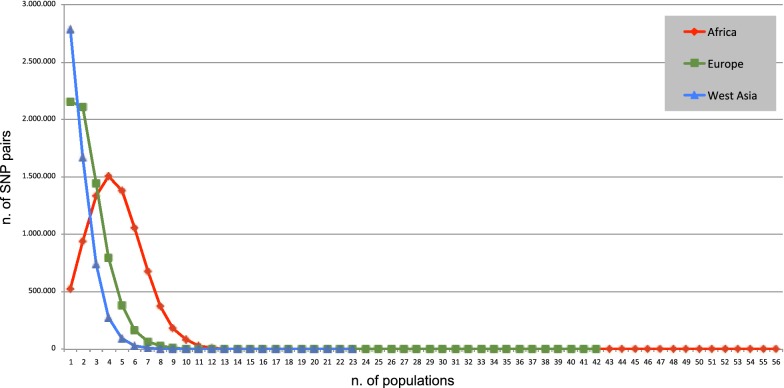
Distribution of SNP pairs in linkage disequilibrium within continents. The Y axis indicates the number of SNP pairs found in LD and the X axis the number of populations in which a given SNP pair was found in LD

Analysis of LD trends at the within-continent level identified clear differences between the African populations and those from Europe and West Asia (Fig. 3). According to the numerical values that describe the between-breed within-continent distribution of the pairs of SNPs in LD (see Additional file 6: Table S3), 8,130,168, 7,163,898 and 5,602,280 SNP pairs are in LD within the African, European and West Asian populations, respectively. Figure 3 shows that, for the European and West Asian populations, a specific pair of SNPs is in LD within one or two populations (mode value = 1 in both cases), and for the African populations, a specific pair of SNPs in LD is shared by three to five populations (mode value = 4), which possibly reflects their low degree of differentiation.

The analysis of effective population sizes identified a general reduction over the last 1000 generations (see Additional file 7: Figure S3), which corresponds approximately to the last 4000 years. Breeds are subdivided roughly into two groups, i.e., with a smaller or larger effective population size. The plots of Ne values across generations (see Additional file 8) show that the gap between these two groups, which is clearly visible in Figure S3 (see Additional file 7: Figure S3) up to ca. 200 generations ago, is still present until recently. A few breeds show different trends: KIL_TR experienced a more marked reduction in Ne compared to the other breeds of the same group. On the contrary, the decrease in Ne is more gradual for the PAT_PK, LND_MZ, MSH_ZW, MTB_ZW, SOF_MG populations. Finally, CRE_AG and RAN_AU gradually pass from the group with the smaller Ne to that with the larger Ne.

Although Ne values that are calculated for large numbers of generations BP can fluctuate more, it is interesting to note that in the plot that refers to 959 generations ago (see Additional file 8), the Ne size for populations from Turkey and Egypt (KIL_TR, KLS_TR, BRK_EG, SID_EG) was larger than that for all other breeds; this situation changes over time and by 366 generations ago, the Ne of these populations is already in line with that of several other African and European breeds.

**Fig. 4:**
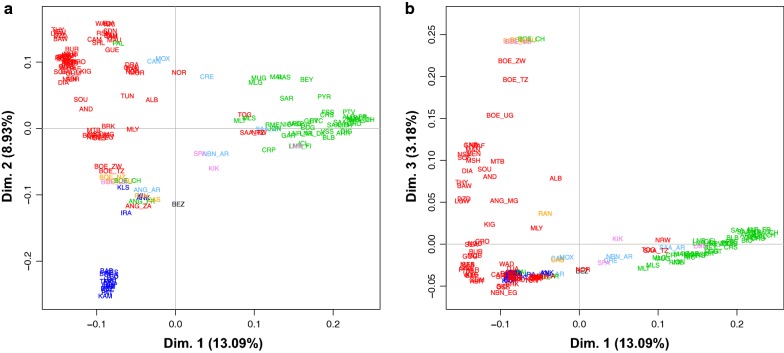
Multi-dimensional scaling plot. Dimension 1 versus 2 (**a**) and dimension 1 versus 3 (**b**). The population labels are coloured according to the continent of origin as follows: red = Africa, green = Europe, blue = West Asia, pink = North America, light blue = South America, orange = Oceania, black = wild goats. To increase readability, the country codes are omitted from the population labels, with the exception of breeds sampled in multiple countries

**Fig. 5:**
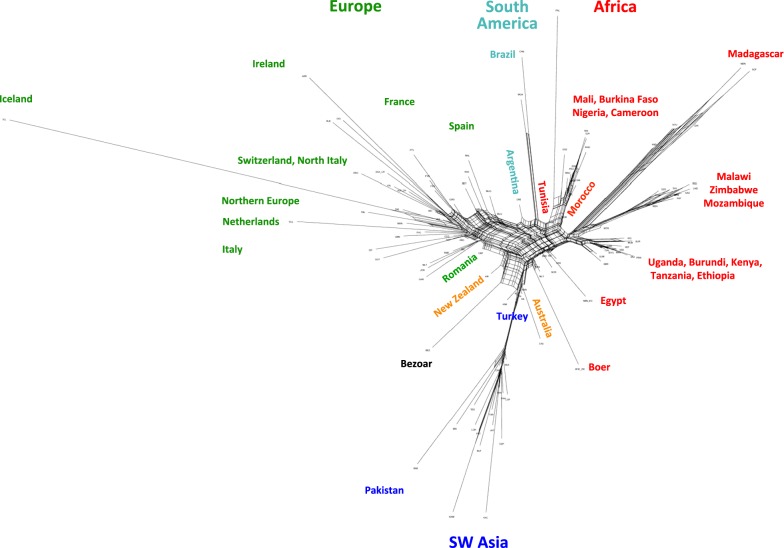
Neighbor-net graph based on between-breed Reynolds distances. Reynolds genetic distances were calculated from the working dataset

**Fig. 6:**
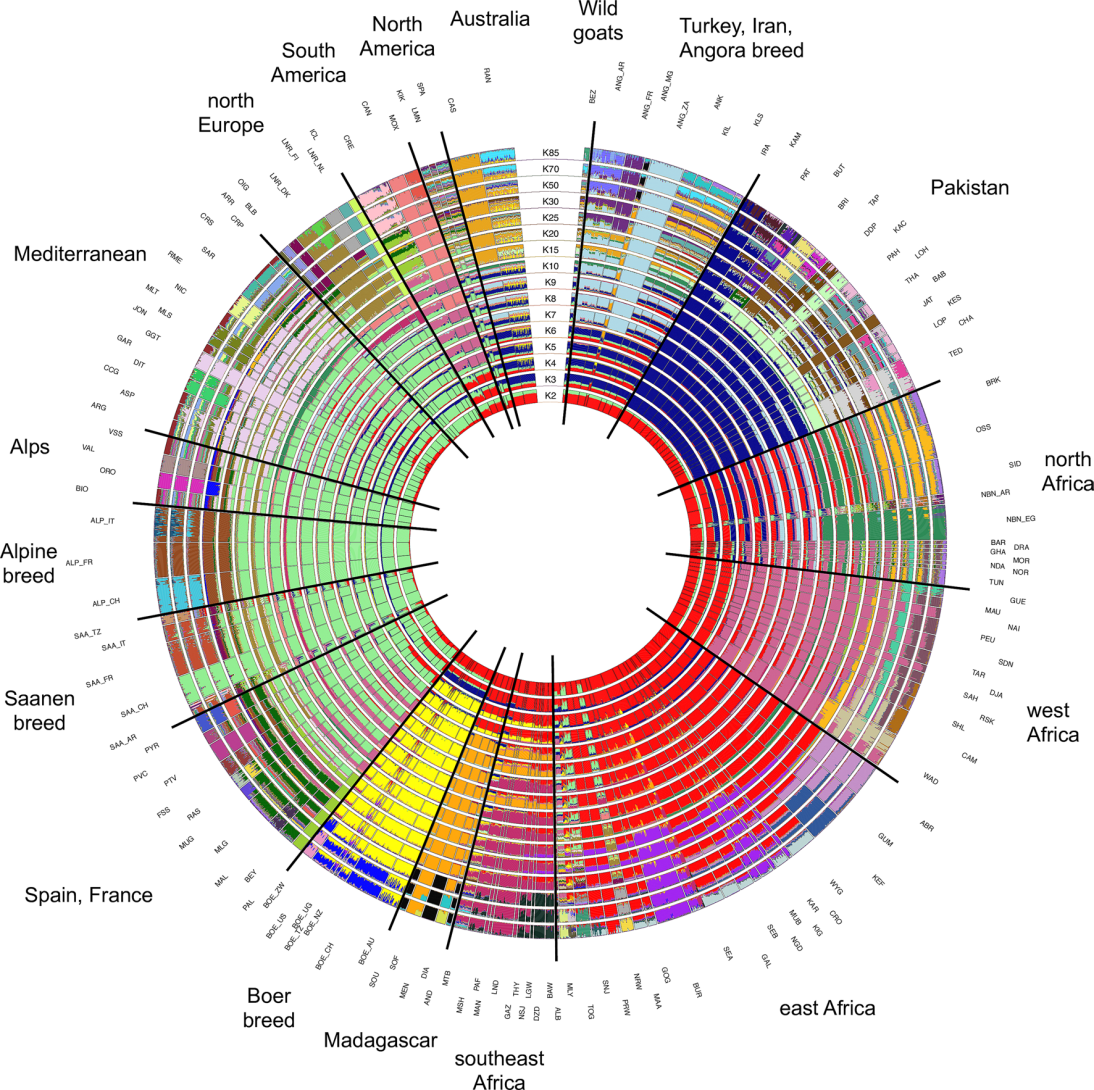
Worldwide population structure of goat breeds included in the AdaptMap project. Circular representation of Admixture software results for K = 2–10, 15, 20, 25, 30, 50, 70 and 85 (lowest cross-validation error value). To increase readability, the country codes are omitted from the population labels, with the exception of breeds sampled in multiple countries

Several analyses (Figs. 4, 5, 6 and Table 1) consistently pointed to remarkable subdivisions in goat genetic variation at the molecular level. A clear differentiation between continental groups of populations was initially highlighted by the plot of the first three dimensions (13.09, 8.93 and 3.18% of the total variance, respectively; cumulated percentage 25.20%) of the multi-dimensional Scaling analysis. As shown in Fig. 4a, dimension 1 differentiated European breeds from the other ones, while dimension 2 separated the Pakistani populations (lower left corner) from the African populations and from another cluster including the Boer, Angora, Turkish and Iranian breeds (IRA_IR, ANK_TR, KLS_TR and KIL_TR). These subdivisions were consistent with the major splits in the structure of the Neighbour-network based on between-breed D_R_ distances calculated from the WD set (see Additional file 9: Table S4 and Fig. 5), and the clustering obtained with Admixture software at K = 2 and 3 (Fig. 6).

**Table 1:** Analysis of molecular variance (AMOVA)

Source of variation	d.f.	Sum of squares	Variance components	Percentage of variation
Working dataset				
Among populations	143	11,575,312.65	1618.40	15.19
Among individuals within pops	3053	28,326,327.21	241.44	2.27
Within individuals	3197	28,118,634.00	8795.32	82.54
Total	6393	68,020,273.86	10,655.16	100.00
Africa + Europe + West Asia				
Among populations	120	10,007,868.13	1649.68	15.53
Among individuals within pops	2608	23,994,627.44	226.37	2.13
Within individuals	2729	23,872,328.00	8747.65	82.34
Total	5457	57,874,823.58	10,623.70	100.00
Africa + Europe + West Asia (continental groups)				
Among groups	2	3,461,484.91	952.57	8.69
Among populations within groups	118	6,546,383.22	1034.74	9.44
Among individuals within pops	2608	23,994,627.44	226.37	2.07
Within individuals	2729	23,872,328.00	8747.65	79.80
Total	5457	57,874,823.58	10,961.32	100.00
Africa				
Among populations	55	2,617,223.31	921.33	9.48
Among individuals within pops	1127	10,167,098.14	225.40	2.32
Within individuals	1183	10,138,998.00	8570.58	88.20
Total	2365	22,923,319.45	9717.31	100.00
Europe				
Among populations	41	2,471,724.52	1072.99	10.17
Among individuals within pops	953	9,269,653.09	246.39	2.33
Within individuals	995	9,187,871.00	9234.04	87.50
Total	1989	20,929,248.61	10,553.42	100.00
West Asia				
Among populations	22	1,455,329.56	1213.15	12.58
Among individuals within pops	528	4,550,804.00	190.75	1.98
Within individuals	551	4,538,829.00	8237.44	85.44
Total	1101	10,544,962.56	9641.35	100.00

This table summarizes the result of AMOVA analyses performed on: the working dataset; 3-continents dataset; 3-continents dataset including the “between continents” hierarchical level; African dataset; European dataset; West Asian dataset. For further details on the preparation of the continental datasets, (see Additional file 1)

*d.f.* degrees of freedom

A more precise estimate of the amount of existing variation corresponding to different hierarchical levels was obtained by analysis of molecular variance (Table 1); 8.69% of the variance was assigned to the “between-groups” level, which accounted for the geographical partitioning between continents (Table 1); 15.19% was allocated to the “between-populations” level, but this percentage varied by continent when the AD, ED and WAD sets were analysed: 9.48% for Africa, 10.17% for Europe and 12.58% for West Asia.

Within-continent substructures were also identified by both the Neighbour-network analysis and Admixture software (Fig. 5; K = 5–8 in Fig. 6). In particular the breeds from Pakistan were separated from the rest of the West Asian group, and within Africa, different sub-groups corresponded to Northwest Africa +Canary islands, East Central Africa and Southeast Africa, which are further subdivided into Madagascar and Malawi + Zimbabwe + Mozambique groups.

In Europe, groups of populations consistent with Spain + Sardinia, France + Corsica, Ireland + Alpine breeds, North Europe (Netherlands, Finland, Norway, Iceland), Central and South Italy were revealed (Fig. 5; K = 10 to 25 in Fig. 6). CRP_RO from Romania, the only Balkan breed, had an intermediate position between South European (Central Italy) and West Asian populations. At high K values, including K = 85 that was identified as the best-fitting resolution based on cross-validation error values (see Additional file 10: Figure S4), several European and Pakistani breeds were individually assigned to distinct clusters. However, for African goats the inferred gene pools still corresponded to geographical areas rather than to single populations.

Populations that are highly isolated due to geographical or demographic barriers were also highlighted by particularly long branches in the Neighbour-network and well-defined clusters in Admixture barplots (Fig. 5; K = 15 and 20 in Fig. 6), such as the insular breeds from Iceland (ICL_IS), Ireland (ARR_IR, BLB_IR, OIG_IR), the Canary Islands (PAL_ES), and Madagascar [54]. The populations from Pakistan, Brazil, Malawi and Mozambique were also highlighted as reproductively isolated, thus confirming the Jaatha software results (Fig. 2 and see Additional file 4: Table S2).

In the case of the wild goats from Iran, BEZ_IR, the long branch in the Neighbour-network probably reflects the combined effects of ascertainment bias, reproductive isolation, bottleneck and distinctiveness.

Remarkable admixture was also highlighted, particularly a strong introgression of African ancestry into the breeds of South America, Spain and Southern Italy (Fig. 5 and K = 2 in Fig. 6), and also into the Palmera goats from the Canary Islands (Fig. 4 and K = 5 in Fig. 6), which was particularly close to breeds from Northeast Africa (GUE_ ML and MAU_ML from Mali, and SAH_BF from Burkina Faso).

The behaviour of transboundary breeds (Boer, Toggenburg, Saanen and Angora) and of those sampled in North America, South America and Oceania varied according to their ancestry (Figs. 4, 5 and 6): they were either close to other populations of their respective breeds (i.e., Boer and Angora; Fig. 4a and b) or to African breeds (CAN_BR and MOX_BR), while those that have an African × European background (SPA_US, KIK_US, LMN_US, NBN_AR) occupy an intermediate position between the clusters of the corresponding continental populations. In particular, Fig. 5 shows that breeds from South America occupy an intermediate position between those from Northwest Africa and South Europe (Spain), whereas those from Australia (CAS_AU and RAN_AU) are close to the Turkish populations.

In the MDS plot of individual genotypes (see Additional file 11: Figure S5), the degree of scattering confirmed the variable genetic background of some populations of recent admixed origin, e.g., CRE_AR, MOX_BR, RAN_AU.

As expected, the coancestry matrix obtained with the fineSTRUCTURE software represented as a heat map (see Additional file 12: Figure S6 and Additional file 13: Figure S7) showed that the highest values of coancestry concentrated along the diagonal and corresponded to within-breed relationships. Larger blocks of high coancestry were also revealed, which involved the breeds from Pakistan and the European populations as a whole, and, within Europe, those from the Alpine region, the Pyrénées and some breeds from Ireland (see Additional file 12: Figure S6 and Additional file 13: Figure S7).

Population clusters identified by the fineSTRUCTURE software agreed closely with breed labels, particularly in the case of European populations, and geographical areas of origin (see Additional file 13: Figure S7 and Additional file 14). In the worldwide analysis, the consecutive levels of differentiation (see Additional file 14) are consistent with the MDS, Admixture and Neighbour-net patterns and support the strong geographical partitioning and admixture described above. In particular, the first and second subdivisions (K = 2 and 3) separated European, African and West Asian breeds, whereas successive hierarchical levels split many South European populations from the other European populations (K = 5), Pakistani goats (K = 6), and West African goats from the Southeast African ones (K = 7). The Palmera goats from the Canary Islands clustered with populations that were sampled around the Gulf of Guinea (Cameroon, Nigeria, Mali, Burkina-Faso). Within Southeast Africa, subclusters corresponded to the breeds from (i) Malawi, Zimbabwe and Mozambique, (ii) Madagascar, (iii) Uganda and Burundi, and (iv) Kenya, Tanzania and Ethiopia.

**Fig. 7:**
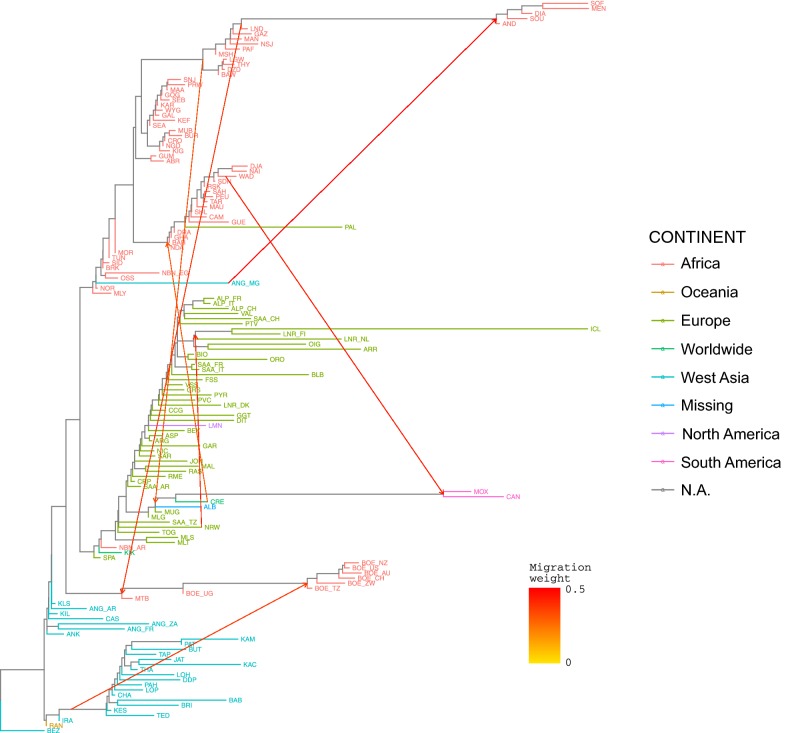
Treemix software graph obtained from the complete dataset and featuring seven migration edges (*m*7)

The variance explained by the Treemix software analysis increased from about 0.85 at *m*0 to 0.95 at *m*11 (see Additional file 15: Figure S8). However, this increase fluctuated between repeated runs for the same *m* value due to the complexity of the dataset and the high degree of admixture between the populations from specific areas (see Additional file 15: Figure S8). Thus, as *m* increased, alternative but recurrent migration edges were highlighted, which often involved admixed or imported breeds. The *m7* graph (Fig. 7) showed the most frequent high-weight edges which linked: (i) the Angora population from Madagascar ANG_MG with the basis of the clade of Madagascan populations (AND_MG, SOU_MG, DIA_MG, SOF_MG, MEN_MG); (ii) the West African Dwarf breed from New Guinea and Cameroon, WAD, with the Brazilian breeds, MOX_BR and CAN_BR; (iii) the breeds from Mozambique, and LND_MZ in particular, with Matabele from Zimbabwe, MTB_ZW; (iv) the basis of the branch that supports the clade of West Asian breeds from Pakistan with the basis of the Boer clade; (v) the population of goats of Norwegian origin reared in Tanzania NRW_TZ with the breeds from North Europe, LNR_FI, ICL_IS, LNR_NL; (vi) the Creole breed from Argentina, CRE_AR, with the basis of the clade that includes the populations from Morocco, the Canary Islands and West Africa; and (vii) the mixed Alpine × Boer breed from Malawi, ALB_MW, with the clade including the other populations from the same country, LGW_MW, THY_MW, DZD_MW, BAW_MW.

When Treemix analysis was performed on the 3CD set, the fraction of variance explained was generally higher and the migration edges showed greater consistency across repeated runs at the same *m* value, in particular up to *m10* (see Additional file 16: Figure S9a). Among the migration edges that were subsequently highlighted, we observed those that link the Pakistani breeds clade with MTB_ZW, MTB_ZW with the Boer populations from Zimbabwe and Tanzania, BOE_TZ and BOE_ZW, which are conversely linked to the Boer population from Uganda and Malya from Tanzania, BOE_UG and MLY_TZ; the basis of the clade that includes the West African populations SDN_ML, WAD, NAI_ML and DJA_BF is linked to the Spanish MUG_ES and MLG_ES breeds. The NOR_MO, RAS_ES and MAL_ES populations formed a clade, which show links with BEY_ES and the group formed by West African populations and the Canarian PAL_ES; the basis of the European group of breeds was linked with the Moroccan populations.

## Discussion

### Ascertainment bias

SNPs have many advantages compared to microsatellites (e.g. [55]), but are susceptible to ascertainment bias, which derives from an over-representation of SNPs that have high MAF in the populations used to identify the polymorphic SNPs (discovery populations). This ascertainment bias typically leads to an underestimation of the genetic diversity in other breeds, which becomes more evident as the relatedness between discovery and test populations decreases, and it affects measures of population differentiation, such as F_ST_ [56]. For this reason, the SNP panel used in the AdaptMap project was developed based on sequence data from a diversified panel of goats, which included Saanen, Alpine, Creole, Boer, Kacang, and Savanna (http://www.goatgenome.org/), and the resulting SNPs were tested on a large set of divergent breeds and populations [30].

**Fig. 8:**
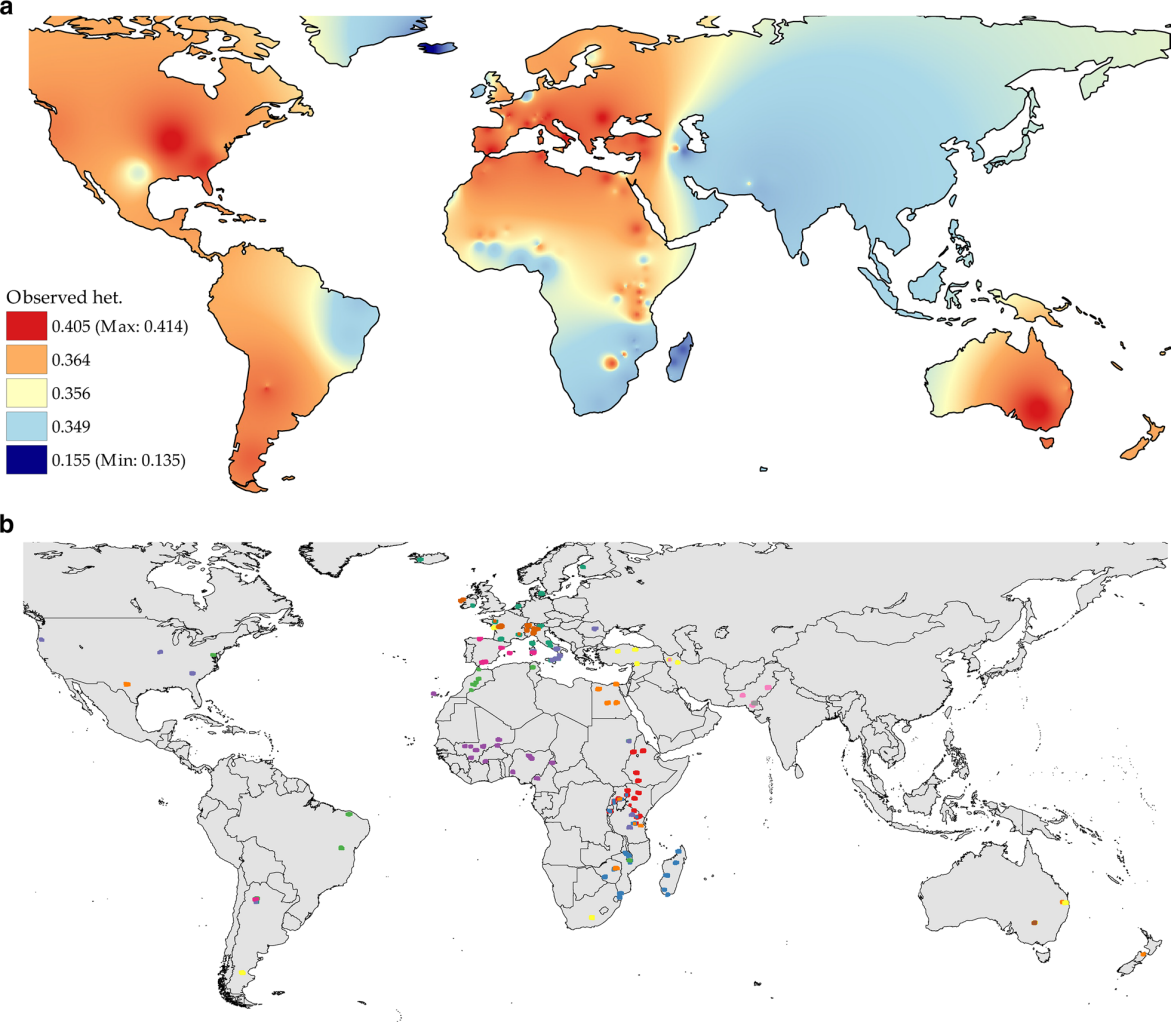
Geographical distribution pattern of: **a** observed heterozygosity, Ho; and **b** Chromopainter software clustering at K = 13

In the case of our dataset, low H_O_ were scored in breeds that are isolated by geographical barriers (e.g. islands) or by strict management procedures. In addition, moderate differences in heterozygosity values between breeds from different continents showed that any potential bias did not appear to have a strong impact on the major outcomes of our analyses. However, we cannot exclude an effect over the slightly lower H_O_ that was scored for populations from specific geographical areas (Fig. 8a), especially for the wild Bezoar (H_O_ = 0.267).

In addition, the inclusion of the Boer breed in the discovery panel may have emphasized its distinctiveness, as suggested by the Admixture results (K = 4; Fig. 6), and the third MDS dimension (Fig. 4b).

### Demographic patterns

Estimates of effective population size (Ne) globally indicated a gradual but constant reduction in size over time, as previously shown at a local scale [31, 32, 57]. Interestingly, the trend in Ne also showed a difference between two groups of breeds, which roughly correspond to populations intensively or extensively managed. For example, the Angora, Boer, Nubian, Cashmere, and to a lesser extent Saanen and Alpine populations all generally showed small Ne values, irrespective of their geographical area of rearing. In contrast, local breeds, such as those from Africa, Spain and Central-Southern Italy, had larger Ne values (see Additional file 7: Figure S3 and Additional file 8).

Likewise, the patterns of gene flow as inferred from the results obtained with the Jaatha software correlated inversely with genetic distances, as represented in Fig. 4 (and Additional file 5: Figure S2), and were generally consistent with both geographical isolation and husbandry regimes. Specifically, out-going gene flow was low for populations in small geographically isolated regions, such as islands and mountainous areas, and their surrounding regions, but higher for populations within these regions. In addition, gene flow seemed to be influenced by husbandry practices, particularly by the decision to establish and maintain populations as formally distinct “breeds”, as is the case in most of Europe and USA. However, these management choices allow maintaining stable breed phenotypes, which can be exploited in analyses of selection signatures to search for genetic variants and chromosomal regions that underlie phenotypic variability [15].

Observed heterozygosity showed a clear association with geography (Fig. 8a), which reflects geo-climatic conditions, geographical isolation and management systems. Limited levels of heterozygosity in breeds from Madagascar, Iceland, the Canary Islands and Ireland are consistent with the hypothesis of lack of gene flow due to physical isolation (MEN_MG, SOF_MG ICL_IS DIA_MG PAL_ES ARR_IE). Similarly, geographical isolation can explain the slightly lower than average values scored for some breeds native to the valleys in the Alps (ORO_IT, VAL_IT also reported by [22]) and in the Pyrénées (PYR_FR). Breed segregation due to management practices is probably responsible for the low H_O_ that characterizes the Malawi populations (BAW_MW, DZD_MW, LGW_MW, NSJ_MW, THY_MW), whereas the high H_O_ observed for populations in East Africa, such as Tanzania, Uganda and Sudan, most likely mirror admixture by cross-breeding.

More generally, an increase in heterozygosity due to pastoralism and nomadism is frequently reported in sheep [58, 59] and is consistent with specific geographical patterns in Iranian goats [60]. In Europe, transhumance is still common in South Italy, which may explain the high H_O_ observed for GAR_IT and ION_IT [22]. In Central and East Africa, yearly changes in climate and pasture availability impose seasonal migrations across geographical areas with different cultures and livestock management practices, which further complicates the pattern of H_O_.

We performed different analyses that confirmed the occurrence of three main gene pools in goats corresponding to Europe, Africa and West Asia, which is similar to the between-continent partitioning of diversity previously observed in cattle [17, 18, 61] and sheep [16]. According to our AMOVA analysis on goat populations, such a subdivision accounts for 8.69% of the global variation, which is higher than that calculated for worldwide sheep (2.98%) [16], and for European versus African taurine cattle (3.2%) [17, 18].

Frequently, continental subdivisions are interpreted as the legacy of gene pool divergence between ancestral populations that migrated out of the domestication centre along different routes [16]. In the case of goats, this view is substantiated by evidence on aDNA that indicates a relationship between the present-day continental gene pools and the highly structured Neolithic populations from different regions of the Fertile Crescent [28].

Thus, compared to sheep, the stronger subdivision observed in goats can be explained either (i) by the hypothesis that the level of population structure in Neolithic ancestors was higher in goats than in sheep, or (ii) that in spite of their similar size, these species have undergone quite different population dynamics at the global scale, with goats experiencing a lower level of post-domestication gene flow compared to the extensive exchanges documented in sheep [16].

### Post-domestication history of goats

In the following section, the finer details of population structure and inferred migration events, obtained here, are combined with previous evidence from archaeological findings, written records and genetic data, to formulate hypotheses on the post-domestication worldwide spread of goat populations.

#### Early migration waves out of the domestication centre

Goat domestication took place ca. 10,500–9900 YBP in the region that covers present-day East Anatolia and Northwest Iran [2, 62]. In general, breeds from the areas close to the domestication centre are expected to have retained partial ancestral diversity, and indeed the goat populations from Turkey and Iran included in our dataset actually display a high level of diversity and a genomic composition similar to that of the wild ancestor, the Bezoar (Fig. 6). Furthermore, at ca. 1000 generations ago (see Additional file 8), these populations had larger Ne than all the other breeds, which likely accounts for the greater genomic variation retained by the populations at that time, and derives from the original variability of the wild ancestors sampled at the time of domestication.

Early domestic goats followed the spread of agriculture and farming by radiating from the Fertile Crescent to Asia, Europe and Africa. As shown by the diversity of aDNA, Neolithic goat populations from Southwest Asia possessed a remarkable genetic structure. Clear distinct gene pools characterized the populations in different areas surrounding the domestication centre, with early domestic goats from the west, east and southwest sides of the Fertile Crescent, respectively, showing genomic affinities with present-day populations from Europe, Asia and Africa [28]. Divergent migration waves, which involved distinct source populations, left their traces in the clear partitioning of diversity between continents, as highlighted by several of our analyses (Figs. 4, 5, and 6 and see Additional file 12). Subsequently, after the Neolithic spread of goats, a geographical within-continent structuring of diversity emerged, as illustrated by goat breeds in Africa and Europe (Figs. 5, 6, and 8b) where the persistence of regional gene pools was further promoted by the high levels of gene flow that characterizes the populations in large areas within both continents (Fig. 2). In the case of Europe, goat populations are partitioned locally into regions corresponding to the east Mediterranean, the central Mediterranean, the east Alps together with continental France, and Ireland and North Europe. The clusters revealed by Chromopainter and fineStructure software (Fig. 8b) differed slightly, which may be due to imperfect phasing of the 52 K genotypes.

Within Africa, breed clusters correspond to West, Northeast, East, Southeast Africa and Madagascar. Interestingly, the geographical distribution of the African gene pools overlaps well with that of groups of breeds that share similar morphological characters [9], i.e., short-eared trypanotolerant breeds of the “West African Dwarf” group in the west-central Africa; lop-eared goats of near eastern ancestry in the northeast; short-eared breeds of the “Small East African” group distributed in the southeast; and lop-eared breeds in the far south.

#### Spread into Europe

The genetic makeup of the European breeds, representing the south, west and north regions of the continent, according to Admixture pattern includes a number of local gene pools (K = 15; Fig. 6) that occur respectively: (i) in northern breeds at high frequency (LNR_DK, LNR_FI, LNR_NL, OIG_IE, BLB_IE, ARR_IE) but also in the Romanian breed from the Carpathians (CRP_RO) and in the breeds from the Alps (e.g. SAA and ALP populations); (ii) in the southwest populations mainly from the Italian peninsula (GGT_IT, ASP_IT, RME_IT, etc.), but also those from Spain and France (e.g. MLG_ES, RAS_ES, MUG_ES and PYR_FR). In northern breeds, the gene flow was lower (Fig. 2) and genetic distances are relatively greater (Figs. 4, 7 and see Additional file 5: Figure S2). These breeds also tended to be positioned further from the centre of the Neighbour-net graph (Fig. 4) or from the root of the Treemix trees (Fig. 7 and see Additional file 16: Figure S9b) compared to Mediterranean and Romanian goats. Presumably, this pattern mirrors the combined effects of the first Neolithic migration, which spread the “proto-European” goat gene pool into the continent, then the influences of African and Asian goats in South Europe (detailed below), and the relatively reduced gene flow in populations in the North (Fig. 2).

#### Spread into the African continent

Considering the distribution of the local gene pools in Africa and the dynamics of the spread of livestock into the continent as described previously in [63], we propose the following scenario: after entering Africa via the Isthmus of Suez, the migration trajectory of goats subsequently bifurcated westwards and southwards along the North and East African coast, respectively. This hypothesis is fully compatible with the Neighbour-net and Treemix patterns and is corroborated by archaeological evidence, which suggests a rapid spread of small ruminants into north and east Africa between 7000 and 5000 YBP [5–7] after their introduction from Southwest Asia [64].

The southbound migration route may have followed the same path already identified for the diffusion of cattle pastoralism to South Africa from the region of the Great Lakes to modern-day Uganda around 2000 YBP and subsequently southwards through the eastern part of the continent [63].

#### Introgression into Africa

After the diffusion of goats into Africa, several subsequent waves of introgression occurred: (i) from Europe to Northwest Africa via Gibraltar and (ii) from Southwest Asia westwards to Northwest Africa along the Mediterranean coast and southwards to the eastern part of the continent. A contribution from the Iberian Peninsula into North African gene pools of livestock has already been identified in several species, including goats [4, 65].

It is interesting to note (Fig. 6; blue cluster from K = 3 to 10) that the western and far-south African gene pools are clearly less influenced by introgression from the West Asian component than other African goats. Conversely, in the north-western and eastern parts of the continent the clearly detected West Asian influence gradually decreases from Egypt to Tunisia to Morocco in the North and from Egypt to Ethiopia, Kenya and Tanzania in the East. This may reflect the original Neolithic immigrations, but the post-domestication introductions of the zebu [63] and the fat-tailed sheep in Eastern Africa indicate the existence of later intense contacts with West Asia, for instance during the spread of Islam into the African continent since the 7th century. Data on goats from the Arabian Peninsula and the regions surrounding the Fertile Crescent may allow to substantiate this hypothesis.

#### West African introgression into Europe and the New World

The West African component is present at high percentages in the Palmera breed from the Canary Islands and in the South American breeds from Brazil and Argentina. The goats in the Canary Islands are generally considered to be descendants of Iberian domesticated goats that were introduced by the Spanish colonists during the last part of the 15th century of the CE [9] and, thus, they subsequently influenced the formation of the South American Creole breeds [66, 67]. However, our results consistently confirm a West African origin (Figs. 5, 6 and 7) as already proposed by Capote et al. [67] based on morphological features. Similarly, the South American goats (CRE from Argentina, but in particular CAN and MOX from Brazil) display a strong component of West African ancestry, which contradicts the usually invoked Spanish origin [9, 68]. This strengthens the evidence already reported for sheep [69] for huge inputs of livestock from the Atlantic coast of Africa and the Atlantic archipelagos into South America, which was mediated by the transatlantic trades since the 16th century.

The same West African gene pool also occurs in Spanish breeds (ca. 40% of the genome) and to a lesser extent also in South European breeds from France and Italy, thus confirming previous evidence of introgression of African livestock gene pool into the Iberian peninsula and Southwest Europe [4].

Treemix migration edges (see Additional file 16: Figure S9b) and the Admixture software pattern (Fig. 6) suggest the occurrence of frequent exchanges between Northwest Africa and the Iberian Peninsula, and interestingly the Admixture software pattern also indicates that this African influence originated from West Africa rather than from Morocco. This suggests a long-distance sea-borne mode of introgression rather than a mostly land route passing through Morocco and the Strait of Gibraltar.

#### The mediterranean crossroads

Besides the presence of the already mentioned West African influence, West Mediterranean goats from Spain and France share genomic components with breeds from Ireland (BLB, ARR, OIG) and North Italy (VAL in particular) (Fig. 6; K = 25). Interestingly, this matches well the diffusion of the proto-Italo-Celtic people during the Bronze Age, which started from ca. 4500 YBP, as described by the distribution of the S116 polymorphism of the human paternal lineages of the R1b haplogroup [70].

Central and East Mediterranean goats from south Italy and Romania were simultaneously influenced by West Africa and Southwest Asia (Fig. 6; blue component at K = 3 and Nubian-Egyptian emerald green component at K = 10; and in the Neighbour-net graph of Fig. 5 their position is closer to the West Asian branches), thus confirming the role of the central Mediterranean region as crossroads of trades and human migrations which had already begun during the prehistoric period. In particular, since the Middle Ages transhumance practices in south Italy [22] may have contributed to spread this introgression into most of the breeds.

Although we cannot provide reliable dates for such gene flow events, we can formulate some hypotheses: the West African influx may have reached Italy either directly from North Africa at the times of the Arab colonization of the West Mediterranean basin or later from Spain mediated by the Spanish Bourbons rulers during the seventeenth to nineteenth centuries. In contrast, the Southwest Asian influx may either date back to the cline established by the Neolithic introduction of domestic goats, or may have occurred in later ages e.g., following the maritime trade routes that crossed the Mediterranean basin in the east–west direction already in prehistoric or early historic times, or during the spread of Islam that started in the seventh century.

#### Formation of breeds, importations, and selection

Since the 18th century, formation of goat breeds started in most of Europe and in several other countries with an organized agricultural infrastructure (e.g., South Africa, North America), via morphological standardization and systematic selection to improve production traits. At the genomic level, these practices caused a remarkably higher degree of LD in European and West Asian breeds than in the African populations, and the emergence of several single-breed clusters in the two high K reconstructions obtained with the Admixture software (Fig. 6). They also resulted in reduced effective population sizes during the last 50 generations (ca. 200 years) which halved in a 22-generation interval (see the 23 and 45 generations panels in Additional file 8). In contrast, during the same period, the African breeds do not seem to have undergone such a comparable reduction in effective population size.

Several recent exports of highly productive or specialized breeds outside their areas of origin are also identified, such as for the European Norwegian, Toggenburg and Saanen goats introduced to Tanzania and Kenya, Boer goats in Oceania, Europe and USA, Nubian and Saanen goats in Argentina, Angora populations bred in several countries worldwide, and Cashmere goats in Australia. This is often followed by intermixing with local gene pools, such as the introgression of the African gene pool into all the European goat populations reared in Tanzania, and the highly admixed nature of the Malya goats reared in the USA, or the Nubian and Saanen goats in Argentina. An exception to this trend is represented by the Cashmere and Angora goats, which still possess a Southwest Asian background although they were exported to Australia, Europe and South America.

The case of the Boer breed represents a remarkable example of introgression combined with human-mediated selective pressures changing over time. In fact, this breed is recorded as having received an input from Indian and European breeds during the 18th or 19th century in order to improve milk production [9, 71], but during the 20th century, the breed was subjected to an intensive selection for meat production.

## Conclusions

Our study represents the widest assessment of goat diversity available to date. By investigating the patterns of population structure, gene flow and migration events, we highlighted a strong and ancient geographical partitioning of diversity between continents. In recent times, the introduction of cosmopolitan highly productive breeds to several countries across the world has led to genomic admixture and introgression into a number of local goat populations. In addition, we observed that geographical and reproductive isolation due to management practices account for gene flow reduction.

We also outlined the major events that characterized the history of this livestock species: from their domestication centre in the Fertile Crescent, early domestic goats spread to Europe, Africa and Asia through divergent migration routes. This has determined the underlying genomic background and partitioning of the continental populations. During the subsequent centuries, geographical and reproductive isolation led to a regional sub-structuring of diversity. Additional and more recent migrations and/or importations spread domestic goats to the Americas and Oceania. At the global scale, our evidence reveals a remarkable level of diversity. Since introgression from cosmopolitan breeds and reduced gene flow may raise concerns about the long-term preservation of goat diversity, our results provide a useful framework for monitoring and protecting the farm animal resources represented by the goat world populations, and may help direct genetic improvement and breeding plans.

## Additional files

**Additional file 1: Table S1.** Summary statistics calculated over the whole set of AdaptMap goat populations. Breed code including country code, breed name, continent and country of provenance, longitude, latitude, number of individuals pre-QC, post-QC and in the working dataset (WD), number of polymorphic loci, observed heterozygosity (H_O_), expected heterozygosity (H_E_) values corrected over the number of usable SNPs, inbreeding coefficient (F_IS_). Underlined figures indicate thinning of large population sizes. Statistical significance: *P < 0.05, **P < 0.01, ***P < 0.005.

**Additional file 2.** Preparation of the continental datasets and list of populations excluded from SNeP software analyses.

**Additional file 3: Figure S1.** Plot of F_IS_ values. Red dots identify populations with statistically significant (*P* < 0.05) values.

**Additional file 4: Table S2.** Pairwise estimates of number of migrants (Nm) and F_ST_. Matrix of the number of migrants calculated between pairs of populations according to Jaatha software (upper diagonal) and of F_ST_ (lower diagonal) based on Arlequin software results. The colour-code assigned to each value follows the same scale adopted in Fig. 2 and in Figure S2 (see Additional File 5: Figure S2). All F_ST_ values are statistically significant at *P* = 0.05, with the exception of those marked with §.

**Additional file 5: Figure S2.** Heatmap of pairwise F_ST_ values. Variation of F_ST_ values calculated pairwise between breeds with Arlequin software. The corresponding numerical values and their statistical significance are given in Table S2 (see Additional file 4: Table S2).

**Additional file 6: Table S3.** Distribution of the pairs of SNPs in Linkage Disequilibrium between populations. The number of SNPs in LD have been calculated between populations within continent for (a) Africa; (b) Europe, and (c) West Asia. The first and second column from the left respectively indicate the number of populations in which a specific pair of SNPs was found in linkage and the corresponding percentage calculated over the total number of populations from that continent.

**Additional file 7: Figure S3.** Effective population size (Ne). Trends in effective population size, Ne, estimated with SNeP software for a number of generations between 13 and 959. For further details (see Additional files 2 and 8).

**Additional file 8.** Single-generation plots of Ne values calculated with SNeP software. The single panels correspond to effective population size values, Ne, estimated for a number of generations before present varying between 13 and 959. Breed circles are coloured according to the continent of provenance: blue = west Asia, green = Europe, red = Africa, light blue = South America, orange = Oceania.

**Additional file 9: Table S4.** Matrix of pairwise Reynolds distances between populations.

**Additional file 10: Figure S4.** Cross-validation error and number of iterations. Description: Cross-validation (CV) error values (upper panel) and number of iterations required to reach convergence (lower panel) calculated for Admixture software runs for K values from 2 to 100. The arrow indicates the K = 85 value with the lowest CV score.

**Additional file 11: Figure S5.** MDS plots of Dimensions 1 versus 2 (panel a) and 1 versus 3 (panel b). Each point represents a single individual. The correspondence between breeds and symbols is given in the legend box in upper right corner.

**Additional file 12: Figure S6.** Heatmap-like representation of Chromopainter coancestry matrix. The structure of the clusters on top of the heatmap is displayed in Figure S7 (see Additional file 13: Figure S7).

**Additional file 13: Figure S7.** Detail of the cluster structure on top of Chromopainter coancestry matrix in Figure S6 [see Additional file 12 Figure S6]. The cluster tree is turned counterclockwise with respect to Figure S6 (see Additional file 12: Figure S6). The rectangles highlight clusters of individuals from the same breed or with the same geographical provenance. The colouring of the boxes is only for visual convenience and there is no strict correspondence between colour and geographical provenance.

**Additional file 14.** Geographical distribution pattern of Chromopainter clustering. Results are shown for clustering solutions for K values from 2 to 14, K = 20 and K = 50.

**Additional file 15: Figure S8.** Fraction of variance explained by repeated runs of Treemix software on the working dataset. Treemix software was run for a number of postulated migration edges, *m*, increasing from *m*0 to *m*11 (3 replicates per *m*).

**Additional file 16: Figure S9.** Additional Treemix software results. The panels represent (a) the fraction of variance explained by repeated runs of Treemix software on the 3-continents dataset for a number of postulated migration edges, *m*, increasing from *m*0 to *m*15 (3 replicates per *m*); and (b) Treemix software graph obtained from the 3-continents dataset and featuring 10 migration edges (*m*10).
