# Supplementary material for: Genome-wide SNP profiling of worldwide goat populations reveals strong partitioning of diversity and highlights post-domestication migration routes
Source: Genet Sel Evol. 2018 Nov 19;50:58. doi: 10.1186/s12711-018-0422-x (PMC6240949; doi:10.1186/s12711-018-0422-x)
Supplement: Supplementary file 1 — Additional file 1: Table S1. Summary statistics calculated over the whole set of AdaptMap goat populations. Breed code including country code, breed name, continent and country of provenance, longitude, latitude, number of individuals pre-QC, post-QC and in the working dataset (WD), number of polymorphic loci, observed heterozygosity (HO), expected heterozygosity (HE) values corrected over the number of usable SNPs, inbreeding coefficient (FIS). Underlined figures indicate thinning of large population sizes. Statistical significance: *P < 0.05, **P < 0.01, ***P < 0.005. [file 12711_2018_422_MOESM1_ESM.docx]

**Table S1**

**Summary statistics calculated over the whole set of ADAPTmap goat populations**.

| **Breed_Country code** | **Breed name** | **Continent** | **Country** | **Long.** | **Lat.** | **N. ind. - raw** | **N. ind. - QC** | **N. ind. - WD** | **n. polym. loci** | **Ho** | **He** | **Fis** |
| --- | --- | --- | --- | --- | --- | --- | --- | --- | --- | --- | --- | --- |
| ABR_ET | Abergelle | Africa | Ethiopia | 38.83 | 13.33 | 53 | 49 | 49 | 47216 | 0.3602 | 0.3652 | 0.0128 |
| ALB_MW | Alpine x Boer | Africa | Malawi | 35.13 | -16.07 | 7 | 5 | 5 | 43600 | 0.4046 | 0.3851 | -0.0582 |
| ALP_CH | Alpine  (Camosciata delle Alpi) | Europe | Switzerland | 7.67 | 46.95 | 67 | 57 | 50 | 48350 | 0.3911 | 0.3926 | 0.0027 |
| ALP_IT |  |  | Italy | 9.25 | 44.94 | 160 | 113 | 50 | 48085 | 0.3985 | 0.4097 | 0.0242 |
| ALP_FR |  |  | France | 0.45 | 46.63 | 52 | 50 | 50 | 48704 | 0.4029 | 0.4129 | 0.0253 |
| AND_MG | Androy | Africa | Madagascar | 45.45 | -25.31 | 7 | 6 | 6 | 40897 | 0.3174 | 0.3238 | 0.0217 |
| ANG_MG | Angora | Worldwide | Madagascar | 44.41 | -24.04 | 7 | 7 | 7 | 42621 | 0.3496 | 0.3478 | 0.0008 |
| ANG_AR |  |  | Argentina | -69.18 | -46.30 | 332 | 217 | 50 | 47299 | 0.3847 | 0.3866 | 0.0258 |
| ANG_FR |  |  | France | -1.23 | 46.38 | 26 | 26 | 26 | 46635 | 0.3454 | 0.3546 | -0.0059 |
| ANG_ZA |  |  | South Africa | 25.02 | -31.49 | 48 | 48 | 48 | 47158 | 0.3246 | 0.3396 | 0.0439 |
| ANK_TR | Ankara | W Asia | Turkey | 31.96 | 39.97 | 20 | 18 | 18 | 46897 | 0.3840 | 0.3955 | 0.0283 |
| ARG_IT | Argentata | Europe | Italy | 15.13 | 38.02 | 25 | 24 | 24 | 48610 | 0.4083 | 0.4168 | 0.0206 |
| ARR_IE | Traditional Arran | Europe | Ireland | -9.89 | 53.31 | 10 | 8 | 8 | 39279 | 0.2936 | 0.2977 | 0.0141 |
| ASP_IT | Aspromontana | Europe | Italy | 15.91 | 37.99 | 24 | 23 | 23 | 48471 | 0.3856 | 0.4102 | 0.0611 |
| BAB_PK | Barbari | W Asia | Pakistan | 72.48 | 30.30 | 17 | 16 | 16 | 38076 | 0.3048 | 0.2921 | -0.0518 |
| BAG_GB | Bagot | Europe | UK | -3.29 | 54.45 | 1 | 1 | N.A. | N.A. | N.A. | N.A. | N.A. |
| BAR_MA | Barcha | Africa | Morocco | -7.17 | 31.09 | 4 | 4 | 4 | 43183 | 0.3811 | 0.3985 | 0.0502 |
| BAW_MW | Balaka-Ulongwe | Africa | Malawi | 34.87 | -14.92 | 12 | 12 | 12 | 43621 | 0.3263 | 0.3356 | 0.0279 |
| BEY_ES | Bermeya | Europe | Spain | -5.26 | 43.34 | 24 | 23 | 23 | 48202 | 0.3985 | 0.3999 | 0.0027 |
| BEZ_IR | Bezoar | W Asia | Iran | 48.07 | 37.06 | 7 | 7 | 7 | 42229 | 0.2669 | 0.3501 | 0.2513*** |
| BIO_IT | Bionda dell'Adamello | Europe | Italy | 10.36 | 46.03 | 24 | 24 | 24 | 48511 | 0.4002 | 0.4090 | 0.0216 |
| BLB_IE | Bilberry | Europe | Ireland | -7.13 | 52.25 | 10 | 10 | 10 | 41600 | 0.3308 | 0.3265 | -0.0158 |
| BOE_AU | Boer | Worldwide | Australia | 151.83 | -27.28 | 61 | 32 | 32 | 47077 | 0.3655 | 0.3619 | -0.0106 |
| BOE_US |  |  | USA | -100.16 | 31.19 | 34 | 29 | 29 | 47355 | 0.3528 | 0.3685 | 0.0054 |
| BOE_TZ |  |  | Tanzania | 38.12 | -6.75 | 4 | 4 | 4 | 41799 | 0.3569 | 0.3835 | -0.0142 |
| BOE_NZ |  |  | New Zealand | 173.43 | -41.33 | 14 | 13 | 13 | 45572 | 0.3656 | 0.3607 | 0.0798 |
| BOE_ZW |  |  | Zimbabwe | 31.55 | -18.19 | 25 | 17 | 17 | 45834 | 0.3808 | 0.3616 | 0.0439 |
| BOE_UG |  |  | Uganda | 32.75 | 0.33 | 5 | 5 | 5 | 44109 | 0.3832 | 0.3996 | 0.0422 |
| BOE_CH |  |  | Switzerland | 7.95 | 46.98 | 190 | 133 | 50 | 48124 | 0.3658 | 0.3680 | -0.0552 |
| BOEx_MW | Admixed Boer | Africa | Malawi | 35.16 | -16.75 | 4 | 3 | N.A. | N.A. | N.A. | N.A. | N.A. |
| BOEx_UG |  | Africa | Uganda | 32.75 | 0.33 | 3 | 3 | N.A. | N.A. | N.A. | N.A. | N.A. |
| BRI_PK | Bari | W Asia | Pakistan | 68.86 | 26.08 | 35 | 25 | 25 | 44876 | 0.3048 | 0.3265 | 0.0659* |
| BRK_EG | Barki | Africa | Egypt | 26.90 | 29.89 | 153 | 106 | 50 | 47465 | 0.3930 | 0.4033 | 0.0212 |
| BUR_BI | Burundi goat | Africa | Burundi | 29.83 | -2.91 | 72 | 66 | 50 | 47891 | 0.3460 | 0.3539 | 0.0212 |
| BUT_PK | Bugituri | W Asia | Pakistan | 68.86 | 26.08 | 40 | 31 | 31 | 44446 | 0.3095 | 0.3236 | 0.0418 |
| CAM_CM | Cameroon Goat | Africa | Cameroon | 14.39 | 10.11 | 40 | 37 | 37 | 47513 | 0.3585 | 0.3665 | 0.0218 |
| CAN_BR | Caninde' | S America | Brazil | -40.34 | -3.75 | 31 | 23 | 23 | 41674 | 0.3344 | 0.3100 | -0.0857* |
| CAS_AU | Cashmere | Oceania | Australia | 152.57 | -27.52 | 48 | 44 | 44 | 47283 | 0.3836 | 0.3757 | -0.0240 |
| CCG_IT | Ciociara Grigia | Europe | Italy | 13.82 | 41.61 | 19 | 16 | 16 | 48155 | 0.3821 | 0.4137 | 0.0783* |
| CHA_PK | Chappar | W Asia | Pakistan | 68.86 | 26.08 | 10 | 9 | 9 | 44843 | 0.3593 | 0.3722 | 0.0358 |
| CRE_AR | Creole | S America | Argentina | -65.25 | -26.43 | 186 | 141 | 50 | 47101 | 0.3671 | 0.3987 | 0.0749*** |
| CRO_UG | Local Cross | Africa | Uganda | 31.02 | -0.07 | 5 | 5 | 5 | 42968 | 0.3669 | 0.3727 | 0.0171 |
| CRP_RO | Carpatian goat | Europe | Romania | 25.78 | 46.12 | 14 | 14 | 14 | 48047 | 0.4068 | 0.4254 | 0.0447 |
| CRS_FR | Corse | Europe | France | 9.00 | 42.19 | 30 | 29 | 29 | 48506 | 0.3850 | 0.4070 | 0.0535 |
| DDP_PK | Dera Din Panah | W Asia | Pakistan | 72.48 | 30.30 | 22 | 20 | 20 | 40702 | 0.3126 | 0.3287 | 0.0404 |
| DIA_MG | Diana | Africa | Madagascar | 48.40 | -13.71 | 17 | 14 | 14 | 41444 | 0.2759 | 0.2974 | 0.0744 |
| DIT_IT | Di Teramo | Europe | Italy | 13.37 | 42.38 | 24 | 19 | 19 | 46444 | 0.3777 | 0.3635 | -0.0408 |
| DJA_BF | Djallonke | Africa | Burkina Faso | -3.53 | 10.56 | 12 | 10 | 10 | 43080 | 0.3386 | 0.3432 | 0.0122 |
| DRA_MA | Draa | Africa | Morocco | -7.61 | 29.62 | 4 | 4 | 4 | 42872 | 0.3791 | 0.3941 | 0.0437 |
| DZD_MW | Dedza | Africa | Malawi | 34.33 | -14.37 | 15 | 15 | 15 | 43358 | 0.3033 | 0.3289 | 0.0795 |
| FSS_FR | Fosses | Europe | France | -1.12 | 47.97 | 26 | 24 | 24 | 48196 | 0.3778 | 0.3986 | 0.0520 |
| GAL_KE | Galla | Africa | Kenya | 37.66 | 2.01 | 23 | 23 | 23 | 47035 | 0.3635 | 0.3691 | 0.0150 |
| GALxSAA_KE | Galla x Saanen | Africa | Kenya | 37.88 | 2.22 | 1 | 1 | N.A. | N.A. | N.A. | N.A. | N.A. |
| GAR_IT | Garganica | Europe | Italy | 15.57 | 40.70 | 20 | 15 | 15 | 42958 | 0.4038 | 0.3829 | -0.0689 |
| GAZ_MZ | Gaza | Africa | Mozambique | 32.58 | -25.97 | 4 | 4 | 4 | 36812 | 0.3161 | 0.3181 | 0.0067 |
| GGT_IT | Girgentana | Europe | Italy | 14.17 | 37.61 | 30 | 24 | 24 | 47085 | 0.3573 | 0.3650 | 0.0208 |
| GHA_MA | Ghazalia | Africa | Morocco | -6.60 | 31.12 | 4 | 4 | 4 | 43017 | 0.3815 | 0.3960 | 0.0424 |
| GOG_TZ | Gogo | Africa | Tanzania | 36.68 | -6.28 | 13 | 12 | 12 | 45693 | 0.3611 | 0.3674 | 0.0171 |
| GSH_UG | Gishu | Africa | Uganda | 32.75 | 0.33 | 3 | 2 | N.A. | N.A. | N.A. | N.A. | N.A. |
| GUE_ML | Guera | Africa | Mali | -9.19 | 14.54 | 25 | 16 | 16 | 46087 | 0.3639 | 0.3548 | -0.0269 |
| GUM_ET | Gumez | Africa | Ethiopia | 36.20 | 12.97 | 41 | 39 | 39 | 47331 | 0.3678 | 0.3704 | 0.0067 |
| ICL_IS | Icelandic Goat | Europe | Iceland | -21.23 | 64.70 | 13 | 11 | 11 | 20869 | 0.1346 | 0.1506 | 0.1105 |
| IRA_IR | Iranian goat | W Asia | Iran | 45.93 | 37.78 | 9 | 9 | 9 | 46986 | 0.3678 | 0.4048 | 0.0959 |
| JAT_PK | Jattan | W Asia | Pakistan | 68.86 | 26.08 | 24 | 15 | 15 | 43089 | 0.3247 | 0.3341 | 0.0252 |
| JON_IT | Jonica | Europe | Italy | 15.57 | 40.70 | 16 | 11 | 11 | 42826 | 0.4092 | 0.3835 | -0.0841 |
| KAC_PK | Kachan | W Asia | Pakistan | 68.86 | 26.08 | 24 | 19 | 19 | 38609 | 0.2802 | 0.2876 | 0.0231 |
| KAM_PK | Kamori | W Asia | Pakistan | 68.86 | 26.08 | 42 | 38 | 38 | 41553 | 0.2872 | 0.2902 | 0.0046 |
| KAR_UG | Karamonja | Africa | Uganda | 34.67 | 2.53 | 20 | 19 | 19 | 46171 | 0.3618 | 0.3695 | 0.0200 |
| KEF_ET | Keffa | Africa | Ethiopia | 37.00 | 7.42 | 49 | 44 | 44 | 46905 | 0.3472 | 0.3585 | 0.0311 |
| KES_PK | Koh-e-sulmani | W Asia | Pakistan | 65.64 | 28.23 | 14 | 13 | 13 | 44210 | 0.3584 | 0.3741 | 0.0380 |
| KIG_UG | Kigezi | Africa | Uganda | 30.97 | -0.44 | 5 | 4 | 4 | 40782 | 0.3747 | 0.3691 | -0.0178 |
| KIK_US | Kiko | N America | USA | -91.10 | 39.87 | 11 | 11 | 11 | 47887 | 0.4108 | 0.4170 | 0.0150 |
| KIL_TR | Kil | W Asia | Turkey | 36.62 | 40.47 | 25 | 23 | 23 | 48228 | 0.3970 | 0.4021 | 0.0123 |
| KLS_TR | Kilis | W Asia | Turkey | 37.12 | 36.72 | 40 | 36 | 36 | 48378 | 0.3895 | 0.4023 | 0.0317 |
| LGW_MW | Lilongwe | Africa | Malawi | 33.78 | -13.98 | 7 | 3 | 3 | 33804 | 0.3260 | 0.3268 | 0.0026 |
| LMN_US | LaMancha | N America | USA | -123.31 | 43.20 | 10 | 8 | 8 | 46219 | 0.3771 | 0.3900 | 0.0350 |
| LND_MZ | Landin | Africa | Mozambique | 32.36 | -25.50 | 33 | 29 | 29 | 46971 | 0.3037 | 0.3215 | 0.0561 |
| LNR_DK | Landrance Goat | Europe | Denmark | 11.44 | 55.56 | 120 | 84 | 50 | 48107 | 0.3569 | 0.3831 | 0.0688** |
| LNR_FI |  |  | Finland | 22.56 | 62.77 | 20 | 20 | 20 | 46390 | 0.3534 | 0.3645 | 0.0307 |
| LNR_NL |  |  | Netherlands | 5.12 | 52.09 | 15 | 15 | 15 | 43499 | 0.3172 | 0.3220 | 0.0148 |
| LOH_PK | Lohri | W Asia | Pakistan | 68.86 | 26.08 | 25 | 17 | 17 | 42644 | 0.3386 | 0.3334 | -0.0220 |
| LOP_PK | Local Pothohari | W Asia | Pakistan | 72.48 | 30.30 | 16 | 13 | 13 | 44096 | 0.3510 | 0.3539 | 0.0058 |
| MAA_TZ | Maasai | Africa | Tanzania | 37.23 | -4.53 | 20 | 18 | 18 | 46242 | 0.3587 | 0.3618 | 0.0083 |
| MAL_ES | Mallorquina | Europe | Spain | 3.03 | 39.55 | 20 | 18 | 18 | 47435 | 0.3575 | 0.3799 | 0.0602 |
| MAN_MZ | Manica | Africa | Mozambique | 32.58 | -25.97 | 3 | 3 | 3 | 34133 | 0.3149 | 0.3237 | 0.0332 |
| MAU_ML | Maure | Africa | Mali | -7.47 | 14.47 | 14 | 13 | 13 | 46328 | 0.3718 | 0.3687 | -0.0090 |
| MEN_MG | Menabe | Africa | Madagascar | 45.13 | -20.16 | 21 | 19 | 19 | 41141 | 0.2524 | 0.2586 | 0.0240 |
| MLG_ES | Malaguena | Europe | Spain | -4.42 | 37.07 | 42 | 40 | 40 | 48276 | 0.4104 | 0.4152 | 0.0108 |
| MLS_IT | Maltese sarda | Europe | Italy | 9.35 | 40.13 | 15 | 12 | 12 | 46018 | 0.3666 | 0.3794 | 0.0332 |
| MLT_IT | Maltese | Europe | Italy | 14.36 | 37.60 | 16 | 16 | 16 | 47552 | 0.3594 | 0.3807 | 0.0569 |
| MLY_TZ | Malya | Worldwide | Tanzania | 36.42 | -6.20 | 12 | 11 | 23 | 48062 | 0.3664 | 0.4043 | 0.0946* |
| MLY_US |  |  | USA | -76.91 | 39.05 | 13 | 12 |  |  |  |  |  |
| MOR_MA | Moroccan goat | Africa | Morocco | -5.39 | 32.64 | 10 | 10 | 10 | 47436 | 0.3743 | 0.4023 | 0.0729 |
| MOX_BR | Moxoto' | S America | Brazil | -40.34 | -3.75 | 30 | 23 | 23 | 44813 | 0.3410 | 0.3341 | -0.0248 |
| MSH_ZW | Mashona | Africa | Zimbabwe | 31.10 | -18.50 | 23 | 22 | 22 | 47165 | 0.3287 | 0.3454 | 0.0493 |
| MTB_ZW | Matebele | Africa | Zimbabwe | 28.51 | -20.55 | 26 | 22 | 22 | 47939 | 0.3881 | 0.3899 | 0.0043 |
| MTBx_ZW | Matabele cross | Africa | Zimbabwe | 30.83 | -20.07 | 24 | 13 | - | N.A. | N.A. | N.A. | N.A. |
| MUB_UG | Mubende | Africa | Uganda | 32.29 | 0.44 | 23 | 18 | 18 | 46188 | 0.3454 | 0.3582 | 0.0365 |
| MUBx_UG | Admixed Mubende | Africa | Uganda | 31.40 | 0.60 | 3 | 3 | - | N.A. | N.A. | N.A. | N.A. |
| MUG_ES | Murciano-Granadina | Europe | Spain | -3.58 | 37.17 | 20 | 20 | 20 | 48121 | 0.4000 | 0.4007 | 0.0016 |
| MYO_US | Myotonic | N America | USA | -84.87 | 35.68 | 1 | 1 | - | N.A. | N.A. | N.A. | N.A. |
| NAI_ML | Naine | Africa | Mali | -7.48 | 11.42 | 17 | 14 | 14 | 44757 | 0.3416 | 0.3427 | 0.0030 |
| NBN_AR | Nubian | Worldwide | Argentina | -65.38 | -27.35 | 20 | 13 | 13 | 45158 | 0.3977 | 0.4191 | 0.0465 |
| NBN_EG |  |  | Egypt | 31.47 | 31.21 | 84 | 79 | 50 | 46438 | 0.3525 | 0.3642 | 0.0276 |
| NDA_MA | Noire de l'Atlas | Africa | Morocco | -6.25 | 31.72 | 4 | 4 | 4 | 42967 | 0.3730 | 0.3967 | 0.0688 |
| NGD_UG | Nganda | Africa | Uganda | 32.58 | 0.32 | 11 | 11 | 11 | 46239 | 0.3665 | 0.3710 | 0.0122 |
| NIC_IT | Nicastrese | Europe | Italy | 16.45 | 38.93 | 25 | 20 | 20 | 48164 | 0.3832 | 0.4125 | 0.0723 |
| NOR_MA | Nord | Africa | Morocco | -5.45 | 35.24 | 4 | 4 | 4 | 44241 | 0.3876 | 0.4122 | 0.0687 |
| NRW_TZ | Norwegian | Africa | Tanzania | 35.20 | -7.24 | 18 | 17 | 17 | 45986 | 0.3851 | 0.3807 | -0.0149 |
| NSJ_MW | Nsanje | Africa | Malawi | 35.17 | -16.75 | 7 | 6 | 6 | 38329 | 0.2996 | 0.3088 | 0.0296 |
| OEG_GB | Old English | Europe | UK | -5.96 | 53.97 | 2 | - | - | N.A. | N.A. | N.A. | N.A. |
| OIG_IE | Old Irish Goat | Europe | Ireland | -9.78 | 53.90 | 20 | 13 | 13 | 42950 | 0.3401 | 0.3337 | -0.0224 |
| OIGx_IE | Old Irish goat cross | Europe | Ireland | -8.78 | 53.52 | 10 | 9 | - | N.A. | N.A. | N.A. | N.A. |
| ORO_IT | Orobica | Europe | Italy | 9.50 | 46.04 | 24 | 22 | 22 | 47197 | 0.3548 | 0.3602 | 0.0145 |
| OSS_EG | Oasis | Africa | Egypt | 29.20 | 26.17 | 72 | 70 | 50 | 47323 | 0.3611 | 0.3920 | 0.0760*** |
| PAF_MZ | Pafuri | Africa | Mozambique | 32.65 | -25.02 | 4 | 4 | 4 | 37464 | 0.3322 | 0.3288 | -0.0127 |
| PAH_PK | Pahari | W Asia | Pakistan | 72.48 | 30.30 | 19 | 19 | 19 | 45282 | 0.3571 | 0.3549 | -0.0079 |
| PAL_ES | Palmera (Canaria island breed) | Europe | Spain | -17.69 | 28.66 | 15 | 15 | 15 | 39687 | 0.2749 | 0.2760 | 0.0037 |
| PAT_PK | Pateri | W Asia | Pakistan | 68.86 | 26.08 | 37 | 27 | 27 | 45369 | 0.3240 | 0.3327 | 0.0253 |
| PEU_ML | Peulh | Africa | Mali | -4.20 | 14.50 | 25 | 22 | 22 | 47169 | 0.3653 | 0.3643 | -0.0028 |
| PRW_TZ | Pare White | Africa | Tanzania | 37.92 | -4.25 | 22 | 19 | 19 | 45360 | 0.3413 | 0.3492 | 0.0225 |
| PTV_FR | Poitevine | Europe | France | 0.35 | 46.50 | 29 | 27 | 27 | 47739 | 0.3599 | 0.3782 | 0.0486 |
| PVC_FR | Provencale | Europe | France | 4.01 | 46.28 | 18 | 17 | 17 | 47740 | 0.4002 | 0.4025 | 0.0048 |
| PYR_FR | Pyrenean | Europe | France | 0.52 | 43.33 | 27 | 26 | 26 | 48079 | 0.3629 | 0.3892 | 0.0685* |
| RAN_AU | Rangeland | Oceania | Australia | 144.25 | -32.88 | 66 | 62 | 50 | 48459 | 0.4093 | 0.4140 | 0.0105 |
| RAS_ES | Blanca de Rasquera | Europe | Spain | 0.61 | 41.00 | 20 | 20 | 20 | 47791 | 0.3672 | 0.3897 | 0.0585 |
| RME_IT | Rossa Mediterranea | Europe | Italy | 15.57 | 40.70 | 45 | 30 | 30 | 45366 | 0.4084 | 0.3984 | -0.0367 |
| RSK_NG | Red Sokoto | Africa | Nigeria | 8.17 | 11.89 | 21 | 19 | 19 | 46875 | 0.3471 | 0.3702 | 0.0636 |
| SAA_AR | Saanen | Worldwide | Argentina | -65.74 | -26.56 | 23 | 16 | 16 | 45327 | 0.3762 | 0.4240 | 0.1114* |
| SAA_CH |  |  | Switzerland | 7.87 | 46.87 | 47 | 41 | 41 | 47767 | 0.3768 | 0.3777 | 0.0008 |
| SAA_FR |  |  | France | 1.70 | 46.61 | 56 | 56 | 50 | 48732 | 0.4057 | 0.4208 | 0.0359 |
| SAA_IT |  |  | Italy | 9.57 | 46.07 | 24 | 23 | 23 | 48620 | 0.4145 | 0.4148 | 0.0005 |
| SAA_TZ |  |  | Tanzania | 35.83 | -3.84 | 19 | 15 | 15 | 45421 | 0.3850 | 0.3948 | 0.0203 |
| SAA_KE |  |  | Kenya | 35.88 | -0.33 | 2 | 2 | - | N.A. | N.A. | N.A. | N.A. |
| SAAxANB_BR | Saanen x Anglo Nubian | S America | Brazil | --4140 | -11.48 | 14 | 13 | - | N.A. | N.A. | N.A. | N.A. |
| SAAxCRE_AR | Saanen x Creole | S America | Argentina | -65.31 | -27.19 | 19 | 13 | - | N.A. | N.A. | N.A. | N.A. |
| SAH_BF | Sahel | Africa | Burkina Faso | -0.39 | 14.73 | 15 | 15 | 15 | 46072 | 0.3573 | 0.3659 | 0.0235 |
| SAR_IT | Sarda | Europe | Italy | 9.22 | 39.71 | 39 | 27 | 27 | 48093 | 0.3880 | 0.4120 | 0.0576 |
| SCL_US | San Clemente | N America | USA | -118.50 | 32.89 | 2 | 1 | - | N.A. | N.A. | N.A. | N.A. |
| SDN_ML | Soudanaise | Africa | Mali | -6.27 | 13.45 | 24 | 22 | 22 | 47184 | 0.3620 | 0.3642 | 0.0060 |
| SEA_KE | Small East African | Africa | Kenya | 36.97 | 0.61 | 31 | 31 | 50 | 48113 | 0.3512 | 0.3779 | 0.0709** |
| SEA_MZ |  |  | Mozambique | 32.30 | -25.97 | 9 | 7 |  |  |  |  |  |
| SEA_UG |  |  | Uganda | 32.07 | 0.58 | 15 | 14 |  |  |  |  |  |
| SEAx_KE | Admixed Small East African | Africa | Kenya | 35.97 | 0.45 | 2 | 2 | - | N.A. | N.A. | N.A. | N.A. |
| SEAxALP_KE | Small East African x Alpine | Africa | Kenya | 35.99 | 0.46 | 3 | 3 | - | N.A. | N.A. | N.A. | N.A. |
| SEAxGAL_KE | Small East African x Galla | Africa | Kenya | 37.19 | 1.39 | 18 | 17 | - | N.A. | N.A. | N.A. | N.A. |
| SEAxSAA_KE | Small East African x Saanen | Africa | Kenya | 35.82 | -0.03 | 2 | 2 | - | N.A. | N.A. | N.A. | N.A. |
| SEAxTOG_KE | Small East African x Toggenburg | Africa | Kenya | 36.86 | 0.57 | 5 | 5 | - | N.A. | N.A. | N.A. | N.A. |
| SEB_UG | Sebei | Africa | Uganda | 34.45 | 1.40 | 24 | 21 | 21 | 46675 | 0.3598 | 0.3607 | 0.0022 |
| SHL_NG | Sahel | Africa | Nigeria | 8.73 | 11.25 | 21 | 19 | 19 | 47287 | 0.3700 | 0.3773 | 0.0195 |
| SID_EG | Saidi | Africa | Egypt | 31.58 | 26.24 | 60 | 58 | 50 | 47514 | 0.3770 | 0.3981 | 0.0499* |
| SNJ_TZ | Sonjo | Africa | Tanzania | 36.32 | -2.70 | 22 | 20 | 20 | 44026 | 0.3570 | 0.3522 | -0.0176 |
| SOF_MG | Sofia | Africa | Madagascar | 47.67 | -16.74 | 24 | 22 | 22 | 38566 | 0.2388 | 0.2582 | 0.0766* |
| SOU_MG | Sud Ouest | Africa | Madagascar | 44.40 | -23.97 | 10 | 8 | 8 | 41451 | 0.3018 | 0.3049 | 0.0103 |
| SPA_US | Spanish | N America | USA | -82.52 | 34.07 | 20 | 17 | 17 | 47249 | 0.3982 | 0.4219 | 0.0560 |
| TAP_PK | Tapri | W Asia | Pakistan | 68.36 | 25.47 | 24 | 22 | 22 | 44259 | 0.3270 | 0.3433 | 0.0421 |
| TAR_ML | Targui | Africa | Mali | -0.05 | 16.27 | 22 | 19 | 19 | 46990 | 0.3632 | 0.3691 | 0.0161 |
| TED_PK | Teddi | W Asia | Pakistan | 72.48 | 30.30 | 51 | 47 | 47 | 46325 | 0.3313 | 0.3445 | 0.0369 |
| TET_MZ | Tete | Africa | Mozambique | 32.58 | -25.97 | 2 | 2 | - | N.A. | N.A. | N.A. | N.A. |
| THA_PK | Thari | W Asia | Pakistan | 68.86 | 26.08 | 16 | 16 | 16 | 44536 | 0.3511 | 0.3577 | 0.0164 |
| THY_MW | Thyolo | Africa | Malawi | 35.13 | -16.07 | 9 | 9 | 9 | 41147 | 0.2906 | 0.3216 | 0.1009 |
| TOG_KE | Toggenburg | Africa | Kenya | 35.76 | -1.15 | 1 | 1 | 20 | 46034 | 0.3877 | 0.3994 | 0.0249 |
| TOG_TZ |  |  | Tanzania | 35.09 | -4.24 | 22 | 19 |  |  |  |  |  |
| TUN_TN | Tunisian | Africa | Tunisia | 9.14 | 35.74 | 23 | 21 | 21 | 48383 | 0.3958 | 0.4039 | 0.0203 |
| VAL_IT | Valdostana | Europe | Italy | 7.38 | 45.71 | 24 | 24 | 24 | 47899 | 0.3645 | 0.3835 | 0.0505 |
| VSS_IT | Valpassiria | Europe | Italy | 11.21 | 46.80 | 24 | 24 | 24 | 48507 | 0.3996 | 0.4119 | 0.0303 |
| WAD_NG | West African Dwarf | Africa | Nigeria | 3.74 | 7.59 | 21 | 21 | 50 | 47157 | 0.3306 | 0.3504 | 0.0566* |
| WAD_CM |  |  | Cameroon | 10.27 | 5.90 | 34 | 34 |  |  |  |  |  |
| WYG_ET | Woyito Guji | Africa | Ethiopia | 37.48 | 5.25 | 46 | 39 | 39 | 47219 | 0.3630 | 0.3666 | 0.0094 |

Breed code including country code, breed name, continent and country of provenance, longitude, latitude, number of individuals pre-QC, post-QC and in the working dataset (WD), number of polymorphic loci, observed heterozygosity (H_O_), expected heterozygosity (H_E_) values corrected over the number of usable loci, inbreeding coefficient (F_IS_). Underlined figures indicate thinning of large population sizes. Statistical significance as follows: *= P<0.05, **= P< 0.01, ***= P< 0.005.
