# Supplementary material for: Genome-wide SNP profiling of worldwide goat populations reveals strong partitioning of diversity and highlights post-domestication migration routes
Source: Genet Sel Evol. 2018 Nov 19;50:58. doi: 10.1186/s12711-018-0422-x (PMC6240949; doi:10.1186/s12711-018-0422-x)
Supplement: Supplementary file 2 — Additional file 2. Preparation of the continental datasets and list of populations excluded from SNeP software analyses. [file 12711_2018_422_MOESM2_ESM.docx]

**Additional File 2**

**Preparation of the continental datasets:**

Starting from the working dataset, separate datasets for the three continents (i.e. Africa, Europe and west Asia) were created. Different criteria were used to define the animals to be included. First, only the breeds that originated from and were bred in the continent were retained. Secondly, some breeds were split into subgroups/merged according to differences/similarities in the genetic make-up detected by the analyses performed on the working dataset. In particular, results from Admixture, Chromopainter and phylogenetic representations (Neighbour-net from Reynolds distances, D_R_) were used to define these rearrangements.

**Africa**

Some breeds of non-African origin or bred outside the continents were removed, in particular NBN_AR, NRW, TOG, MLY population sampled in the USA, ALB, BOE_AU, BOE_CH, BOE_NZ, and BOE_US.

NBN_EG breed was split into two groups based on sampling information: the group named NBN_EG1 included the animals EG_NBN0003, EG_NBN0004, EG_NBN0005, EG_NBN0007, EG_NBN0021, EG_NBN0023, EG_NBN0028, EG_NBN0030, EG_NBN0031, and EG_NBN0035; all other animals were included in group NBN_EG2).

WAD breed was split in two groups based on the sampling location: Nigeria (WAD_NG) and Cameroon (WAD_CM). The latter group was further split into two sub-groups based on the genetic pattern detected by Admixture analyses: group WAD_CM1 included the animals CM_WAD0024, CM_WAD0026, CM_WAD0028, CM_WAD0046, CM_WAD0052, and CM_WAD0054; all other animals were assigned to group WAD_CM2).

SEA was split into three subsets based on sampling information: SEA_KE from Kenya, SEA_MZ from Mozambique and SEA_UG from Uganda.

MSH was split in two groups based on genetic pattern highlighted by Admixture analyses: group MSH_1 included the animals ZW_MSH0001, ZW_MSH0002, ZW_MSH0003, ZW_MSH0004, ZW_MSH0005, ZW_MSH0006, ZW_MSH0007, ZW_MSH0015, ZW_MSH0016, ZW_MSH0017, ZW_MSH0018, ZW_MSH0020, and ZW_MSH0021; all other animals were included into group MSH_2.

The pooling of populations based on shared genetic make-up has been done as follows: genetic pool from Morocco (gMA) included the populations BAR, DRA, GHA, and NDA; genetic pool from Burkina Faso and Mali (gML) included the populations SAH, TAR, PEU, and MAU; genetic pool from Uganda (gUG) included the populations MUB, NGD, and KIG; genetic pool from Malawi (gMW) included the populations BAW, DZD, and LGW.

In total the African dataset comprised 1,183 animals, subdivided into 56 populations, and 48827 markers.

**Europe**

Only the LNR_DK breed was split into two subgroups based on Admixture results: group LNR_DK1 included the animals DK_LNR0022, DK_LNR0030, DK_LNR0033, DK_LNR0035, DK_LNR0047, DK_LNR0049, DK_LNR0063, DK_LNR0064, DK_LNR0065, DK_LNR0067, DK_LNR0068, DK_LNR0098, and DK_LNR0117; all other animals were included into group LNR_DK2.

In total the European dataset comprised 995 animals, subdivided into 42 populations, and 48,827 markers.

**Southwest Asia**

The Cashmere breed (CAS) was sampled in Australia but its ancestral geographical origin is Turkey. Since this breed has strongly contributed to the formation of some modern breeds worldwide, it was considered as belonging to the west Asian continental dataset.

For similar reasons, the Angora breed sampled in different locations (in France ANG_FR, and South Africa ANG_ZA) was retained as well.

In total the west Asian dataset comprised 551 animals, subdivided into 23 populations, and 48,827 markers

For Treemix analyses, the wild goat (Bezoar BEZ_IR) population was added as an outgroup.

**List of populations excluded from SNeP software analyses due to small sample size (n. individuals <22).**

| **Breed_Country code** | **Breed name** | **Continent** | **Country** |
| --- | --- | --- | --- |
| LGW_MW | Lilongwe | Africa | Malawi |
| MAN_MZ | Manica | Africa | Mozambique |
| BAR_MA | Barcha | Africa | Morocco |
| BOE_TZ | Boer | worldwide | Tanzania |
| DRA_MA | Draa | Africa | Morocco |
| GAZ_MZ | Gaza | Africa | Mozambique |
| GHA_MA | Ghazalia | Africa | Morocco |
| KIG_UG | Kigezi | Africa | Uganda |
| NDA_MA | Noire de l'Atlas | Africa | Morocco |
| NOR_MA | Nord | Africa | Morocco |
| PAF_MZ | Pafuri | Africa | Mozambique |
| ALB_MW | Alpine x Boer | Africa | Malawi |
| BOE_UG | Boer | worldwide | Uganda |
| CRO_UG | Local Cross | Africa | Uganda |
| AND_MG | Androy | Africa | Madagascar |
| NSJ_MW | Nsanje | Africa | Malawi |
| ANG_MG | Angora | worldwide | Madagascar |
| BEZ_IR | Bezoar | west Asia | Iran |
| ARR_IE | Traditional Arran | Europe | Ireland |
| LMN_US | LaMancha | North America | Usa |
| SOU_MG | Sud Ouest | Africa | Madagascar |
| CHA_PK | Chappar | west Asia | Pakistan |
| IRA_IR | Iranian goat (unknown) | west Asia | Iran |
| THY_MW | Thyolo | Africa | Malawi |
| BLB_IE | Bilberry | Europe | Ireland |
| DJA_BF | Djallonke | Africa | Burkina Faso |
| MOR_MA | Moroccan goat (unknown) | Africa | Morocco |
| ICL_IS | Icelandic Goat | Europe | Iceland |
| JON_IT | Jonica | Europe | Italy |
| KIK_US | Kiko | North America | Usa |
| NGD_UG | Nganda | Africa | Uganda |
| BAW_MW | Balaka-Ulongwe | Africa | Malawi |
| GOG_TZ | Gogo | Africa | Tanzania |
| MLS_IT | Maltese sarda | Europe | Italy |
| BOE_NZ | Boer | worldwide | New Zealand |
| KES_PK | Koh-e-sulmani | west Asia | Pakistan |
| LOP_PK | Local Pothohari | west Asia | Pakistan |
| MAU_ML | Maure | Africa | Mali |
| NBN_AR | Nubian | worldwide | Argentina |
| OIG_IE | Old Irish Goat | Europe | Ireland |
| CRP_RO | Carpatian goat | Europe | Romania |
| DIA_MG | Diana | Africa | Madagascar |
| NAI_ML | Naine | Africa | Mali |
| DZD_MW | Dedza | Africa | Malawi |
| GAR_IT | Garganica | Europe | Italy |
| JAT_PK | Jattan | west Asia | Pakistan |
| LNR_NL | Landrance Goat | Europe | Netherlands |
| PAL_ES | Palmera (Canaria breed) | Europe | Spain |
| SAA_TZ | Saanen | worldwide | Tanzania |
| SAH_BF | Sahel | Africa | Burkina Faso |
| BAB_PK | Barbari | west Asia | Pakistan |
| CCG_IT | Ciociara Grigia | Europe | Italy |
| GUE_ML | Guera | Africa | Mali |
| MLT_IT | Maltese | Europe | Italy |
| SAA_AR | Saanen | worldwide | Argentina |
| THA_PK | Thari | west Asia | Pakistan |
| BOE_ZW | Boer | worldwide | Zimbabwe |
| LOH_PK | Lohri | west Asia | Pakistan |
| NRW_TZ | Norwegian | Africa | Tanzania |
| PVC_FR | Provencale | Europe | France |
| SPA_US | Spanish | North America | Usa |
| ANK_TR | Ankara | west Asia | Turkey |
| MAA_TZ | Maasai | Africa | Tanzania |
| MAL_ES | Mallorquina | Europe | Spain |
| MUB_UG | Mubende | Africa | Uganda |
| DIT_IT | Di Teramo | Europe | Italy |
| KAC_PK | Kachan | west Asia | Pakistan |
| KAR_UG | Karamonja | Africa | Uganda |
| MEN_MG | Menabe | Africa | Madagascar |
| PAH_PK | Pahari | west Asia | Pakistan |
| PRW_TZ | Pare White | Africa | Tanzania |
| RSK_NG | Red Sokoto | Africa | Nigeria |
| SHL_NG | Sahel | Africa | Nigeria |
| TAR_ML | Targui | Africa | Mali |
| DDP_PK | Dera Din Panah | west Asia | Pakistan |
| LNR_FI | Landrance Goat | Europe | Finland |
| MUG_ES | Murciano-Granadina | Europe | Spain |
| NIC_IT | Nicastrese | Europe | Italy |
| RAS_ES | Blanca de Rasquera | Europe | Spain |
| SNJ_TZ | Sonjo | Africa | Tanzania |
| TOG_KE | Toggenburg | Africa | Kenya |
| SEB_UG | Sebei | Africa | Uganda |
| TOG_TZ | Toggenburg | Africa | Tanzania |
| TUN_TN | Tunisian | Africa | Tunisia |
