# Supplementary figures and images for: Genome-wide SNP profiling of worldwide goat populations reveals strong partitioning of diversity and highlights post-domestication migration routes
Source: Genet Sel Evol. 2018 Nov 19;50:58. doi: 10.1186/s12711-018-0422-x (PMC6240949; doi:10.1186/s12711-018-0422-x)

$F_{IS}$

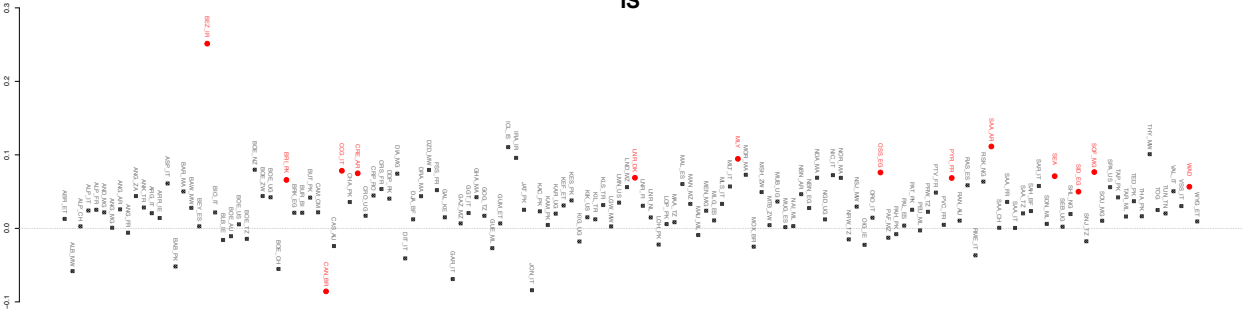

Supplement: Supplementary file 3 — Additional file 3: Figure S1. Plot of FIS values. Red dots identify populations with statistically significant (P < 0.05) values. [file 12711_2018_422_MOESM3_ESM.pdf]

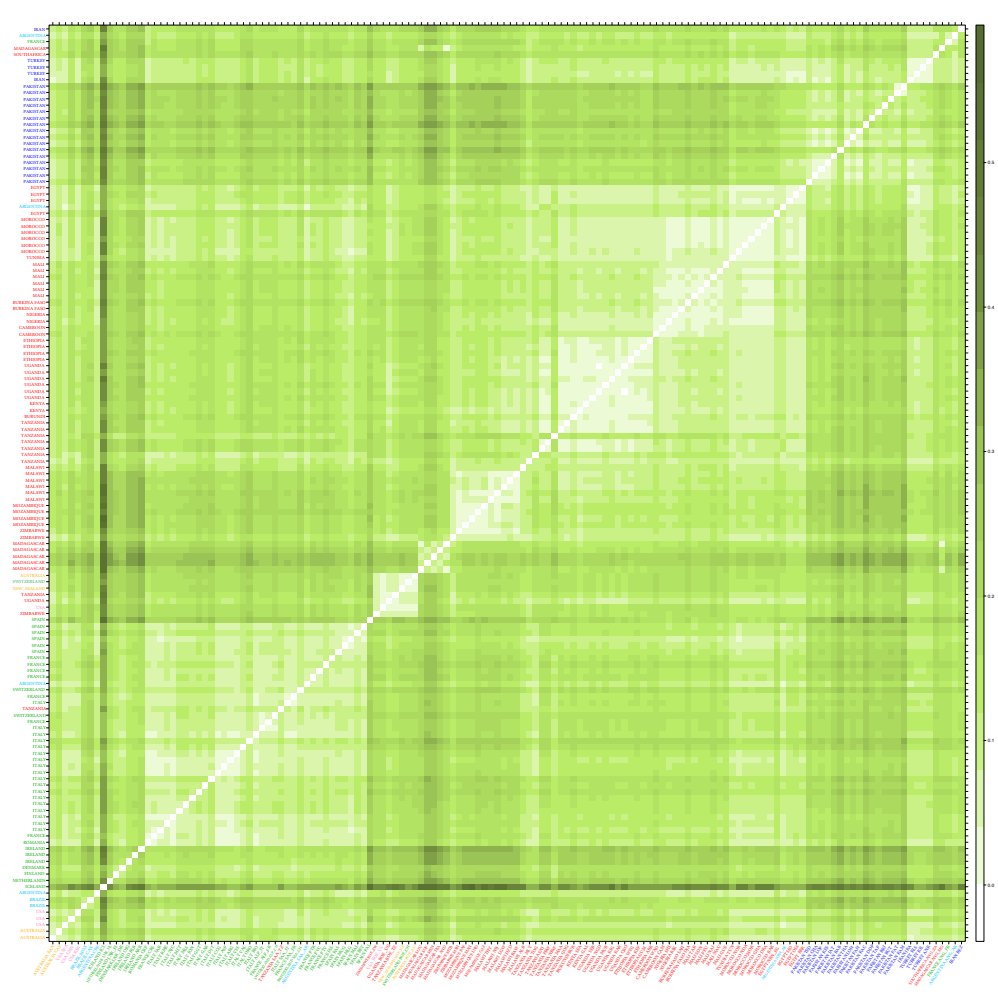

Supplement: Supplementary file 5 — Additional file 5: Figure S2. Heatmap of pairwise FST values. Variation of FST values calculated pairwise between breeds with Arlequin software. The corresponding numerical values and their statistical significance are given in Table S2 (see Additional file 4: Table S2). [file 12711_2018_422_MOESM5_ESM.pdf]

Ne

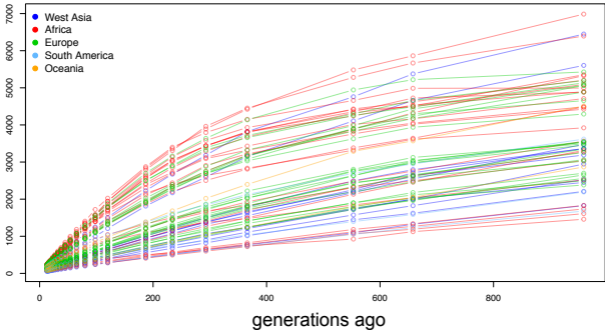

Supplement: Supplementary file 7 — Additional file 7: Figure S3. Effective population size (Ne). Trends in effective population size, Ne, estimated with SNeP software for a number of generations between 13 and 959. For further details (see Additional files 2 and 8). [file 12711_2018_422_MOESM7_ESM.pdf]

CV error

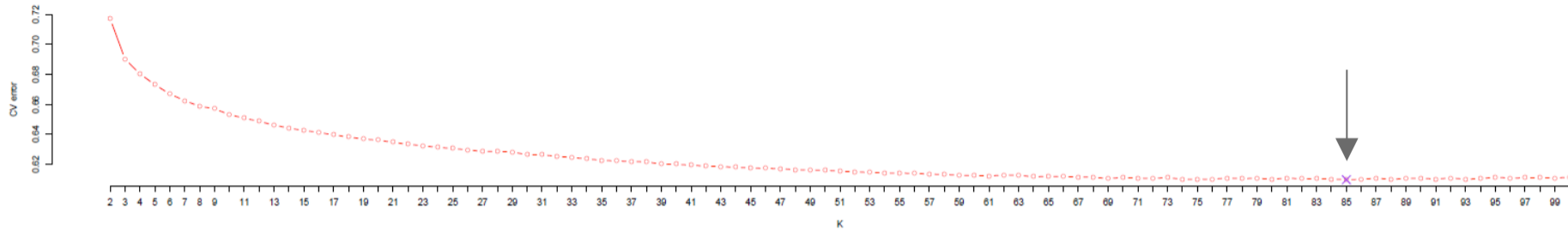

Number of iterations

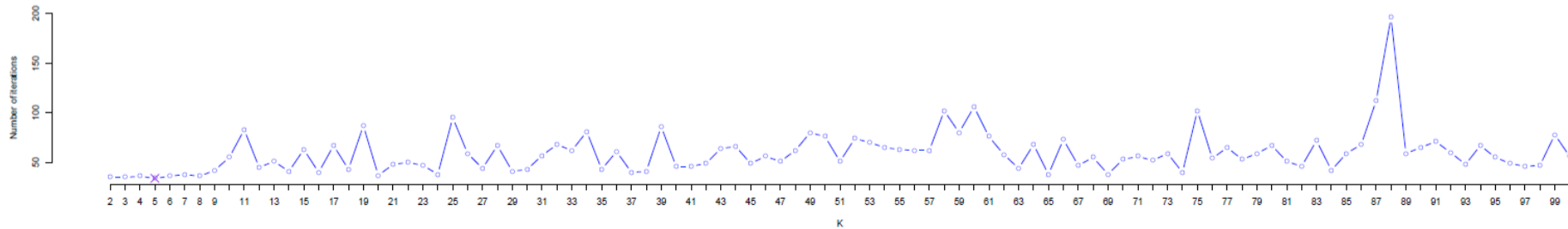

Supplement: Supplementary file 10 — Additional file 10: Figure S4. Cross-validation error and number of iterations. Description: Cross-validation (CV) error values (upper panel) and number of iterations required to reach convergence (lower panel) calculated for Admixture software runs for K values from 2 to 100. The arrow indicates the K = 85 value with the lowest CV score. [file 12711_2018_422_MOESM10_ESM.pdf]

a)

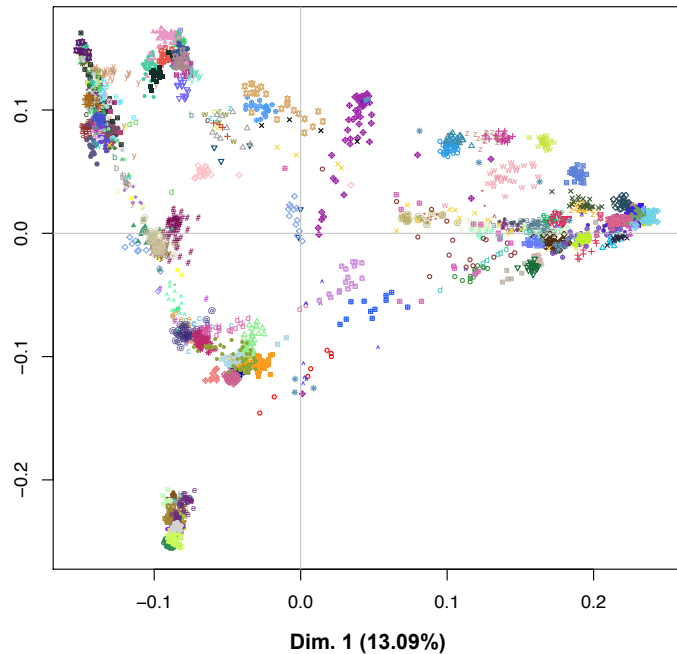

b)

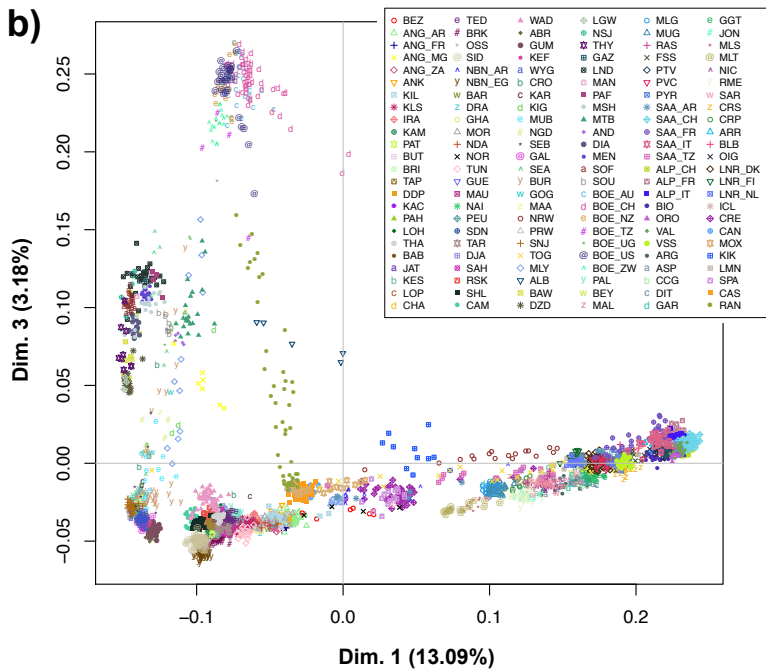

Supplement: Supplementary file 11 — Additional file 11: Figure S5. MDS plots of Dimensions 1 versus 2 (panel a) and 1 versus 3 (panel b). Each point represents a single individual. The correspondence between breeds and symbols is given in the legend box in upper right corner. [file 12711_2018_422_MOESM11_ESM.pdf]

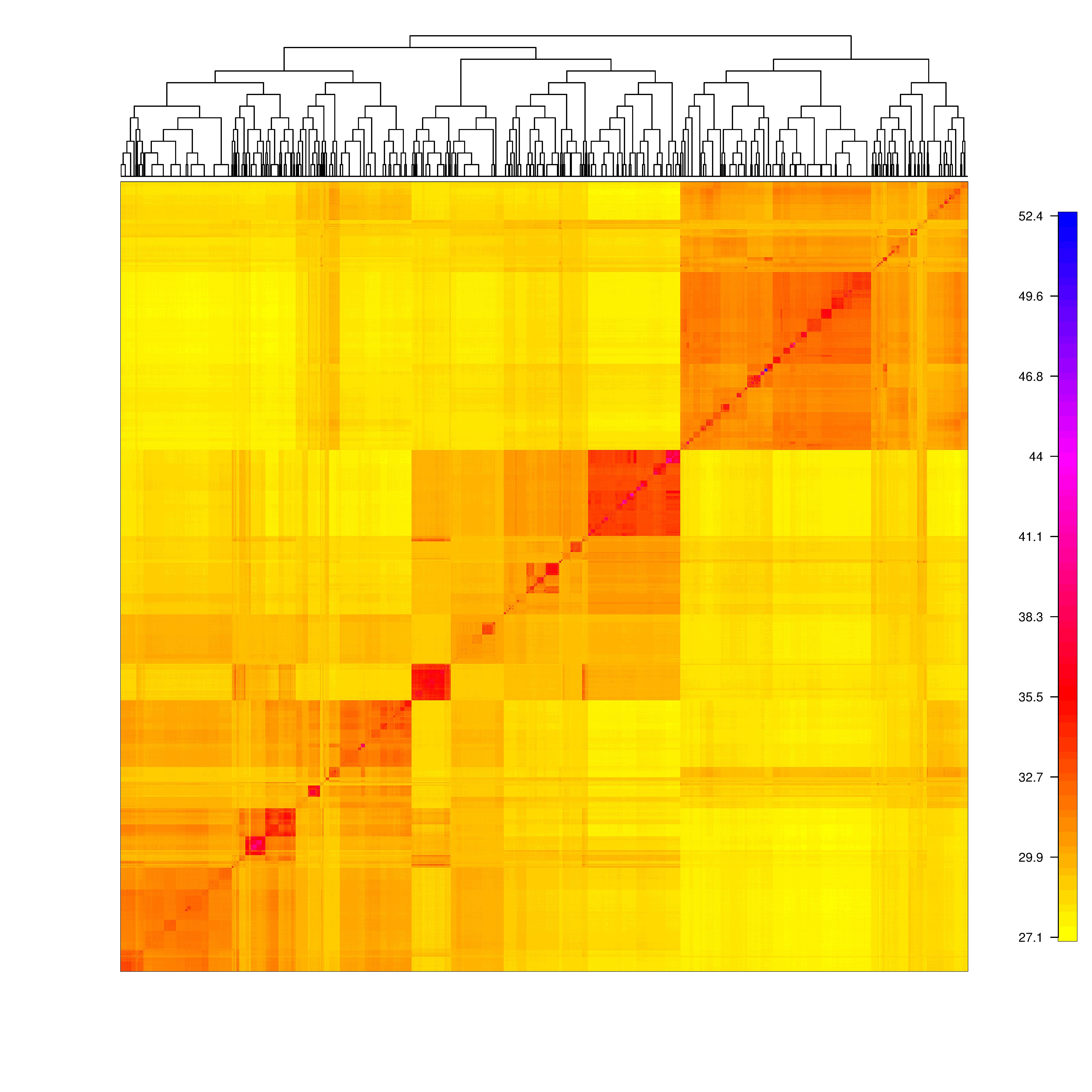

Supplement: Supplementary file 12 — Additional file 12: Figure S6. Heatmap-like representation of Chromopainter coancestry matrix. The structure of the clusters on top of the heatmap is displayed in Figure S7 (see Additional file 13: Figure S7). [file 12711_2018_422_MOESM12_ESM.tiff]

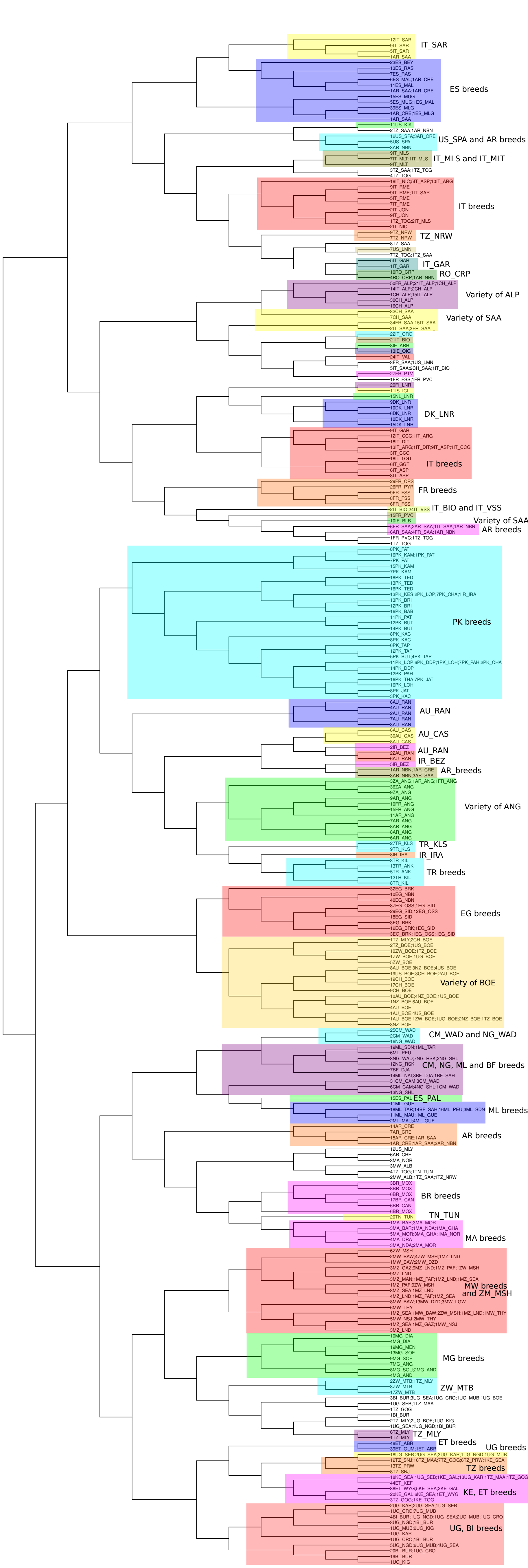

Supplement: Supplementary file 13 — Additional file 13: Figure S7. Detail of the cluster structure on top of Chromopainter coancestry matrix in Figure S6 [see Additional file 12 Figure S6]. The cluster tree is turned counterclockwise with respect to Figure S6 (see Additional file 12: Figure S6). The rectangles highlight clusters of individuals from the same breed or with the same geographical provenance. The colouring of the boxes is only for visual convenience and there is no strict correspondence between colour and geographical provenance. [file 12711_2018_422_MOESM13_ESM.pdf]

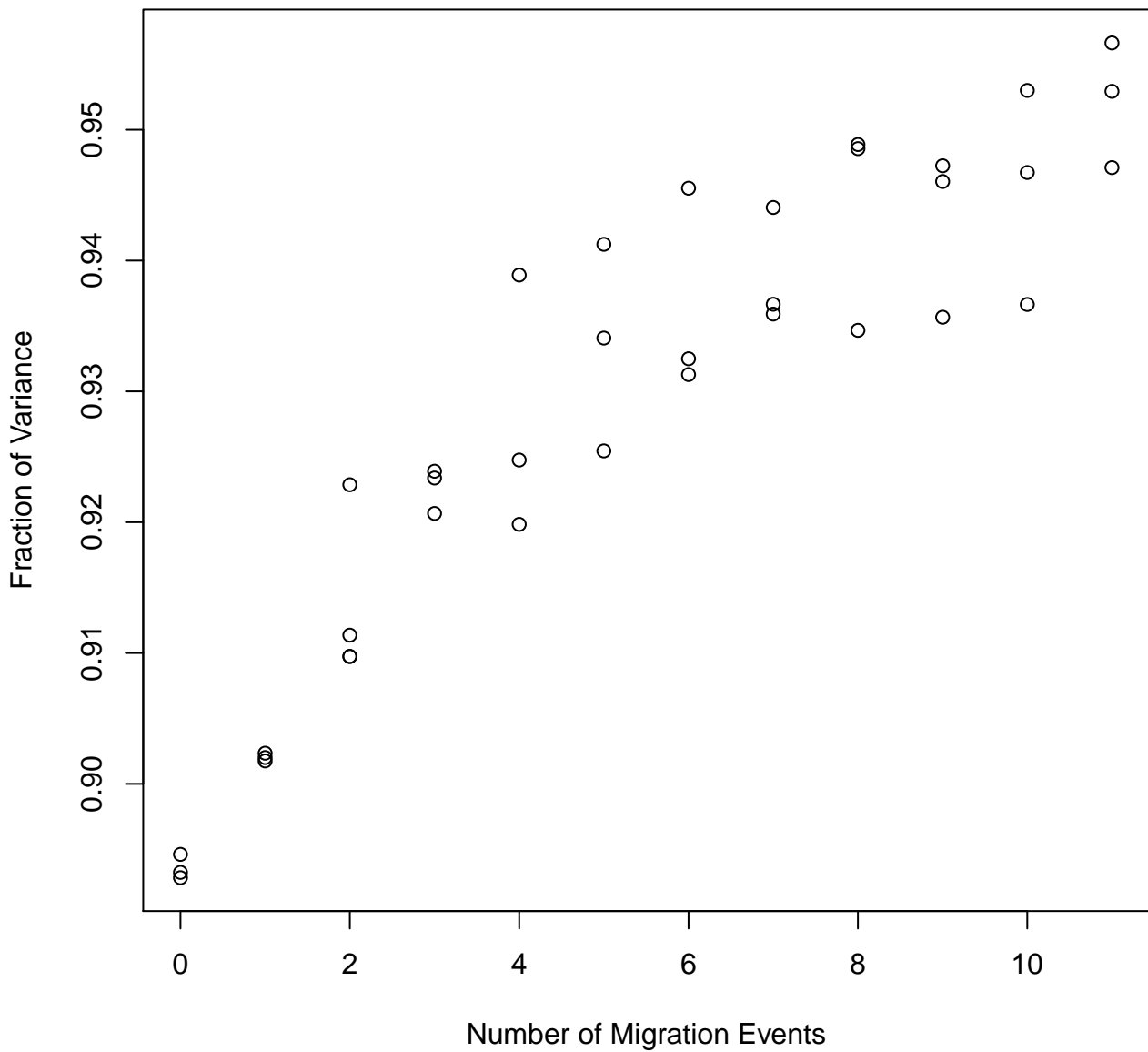

Supplement: Supplementary file 15 — Additional file 15: Figure S8. Fraction of variance explained by repeated runs of Treemix software on the working dataset. Treemix software was run for a number of postulated migration edges, m, increasing from m0 to m11 (3 replicates per m). [file 12711_2018_422_MOESM15_ESM.pdf]

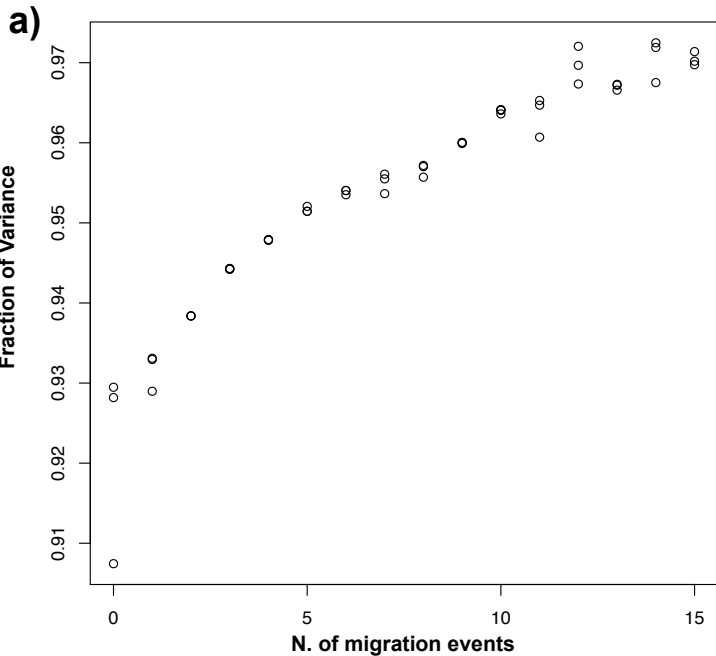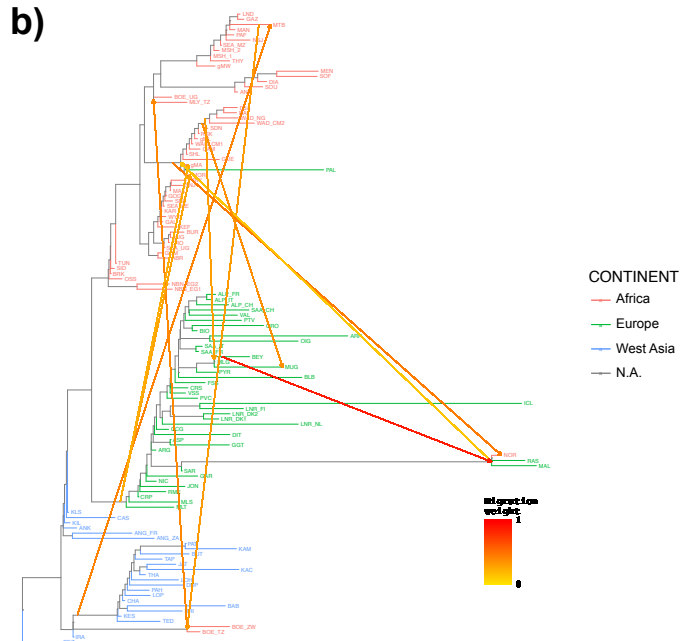

Supplement: Supplementary file 16 — Additional file 16: Figure S9. Additional Treemix software results. The panels represent (a) the fraction of variance explained by repeated runs of Treemix software on the 3-continents dataset for a number of postulated migration edges, m, increasing from m0 to m15 (3 replicates per m); and (b) Treemix software graph obtained from the 3-continents dataset and featuring 10 migration edges (m10). [file 12711_2018_422_MOESM16_ESM.pdf]
