# Supplementary material for: Genome-wide SNP profiling of worldwide goat populations reveals strong partitioning of diversity and highlights post-domestication migration routes
Source: Genet Sel Evol. 2018 Nov 19;50:58. doi: 10.1186/s12711-018-0422-x (PMC6240949; doi:10.1186/s12711-018-0422-x)
Supplement: Supplementary file 6 — Additional file 6: Table S3. Distribution of the pairs of SNPs in Linkage Disequilibrium between populations. The number of SNPs in LD have been calculated between populations within continent for (a) Africa; (b) Europe, and (c) West Asia. The first and second column from the left respectively indicate the number of populations in which a specific pair of SNPs was found in linkage and the corresponding percentage calculated over the total number of populations from that continent. [file 12711_2018_422_MOESM6_ESM.docx]

**Table S3**

**Distribution of the pairs of SNPs in Linkage Disequilibrium within and across populations.**

| **a)** | |  | **b)** | |  | **c)** | |
| --- | --- | --- | --- | --- | --- | --- | --- |
| **AFRICA** | |  | **EUROPE** | |  | **WEST ASIA** | |
| N. breeds | N. of SNP pairs |  | N. breeds | N. of SNP pairs |  | N. breeds | N. of SNP pairs |
| 1 | 520708 |  | 1 | 2157459 |  | 1 | 2781751 |
| 2 | 943331 |  | 2 | 2106756 |  | 2 | 1670774 |
| 3 | 1333927 |  | 3 | 1446227 |  | 3 | 740993 |
| 4 | 1505504 |  | 4 | 794297 |  | 4 | 274997 |
| 5 | 1382982 |  | 5 | 376705 |  | 5 | 89091 |
| 6 | 1056209 |  | 6 | 161913 |  | 6 | 27351 |
| 7 | 678821 |  | 7 | 65692 |  | 7 | 8637 |
| 8 | 373514 |  | 8 | 25753 |  | 8 | 2998 |
| 9 | 181403 |  | 9 | 10508 |  | 9 | 1411 |
| 10 | 79589 |  | 10 | 4608 |  | 10 | 834 |
| 11 | 33319 |  | 11 | 2480 |  | 11 | 608 |
| 12 | 14358 |  | 12 | 1597 |  | 12 | 457 |
| 13 | 6793 |  | 13 | 1174 |  | 13 | 368 |
| 14 | 3799 |  | 14 | 958 |  | 14 | 347 |
| 15 | 2494 |  | 15 | 823 |  | 15 | 239 |
| 16 | 1848 |  | 16 | 660 |  | 16 | 209 |
| 17 | 1391 |  | 17 | 588 |  | 17 | 192 |
| 18 | 1038 |  | 18 | 482 |  | 18 | 193 |
| 19 | 929 |  | 19 | 456 |  | 19 | 181 |
| 20 | 814 |  | 20 | 439 |  | 20 | 172 |
| 21 | 595 |  | 21 | 392 |  | 21 | 152 |
| 22 | 555 |  | 22 | 346 |  | 22 | 156 |
| 23 | 518 |  | 23 | 270 |  | 23 | 169 |
| 24 | 435 |  | 24 | 284 |  | Total | 5602280 |
| 25 | 379 |  | 25 | 262 |  |  |  |
| 26 | 333 |  | 26 | 247 |  |  |  |
| 27 | 316 |  | 27 | 224 |  |  |  |
| 28 | 312 |  | 28 | 197 |  |  |  |
| 29 | 292 |  | 29 | 171 |  |  |  |
| 30 | 246 |  | 30 | 163 |  |  |  |
| 31 | 188 |  | 31 | 186 |  |  |  |
| 32 | 197 |  | 32 | 156 |  |  |  |
| 33 | 218 |  | 33 | 181 |  |  |  |
| 34 | 165 |  | 34 | 134 |  |  |  |
| 35 | 189 |  | 35 | 158 |  |  |  |
| 36 | 173 |  | 36 | 162 |  |  |  |
| 37 | 158 |  | 37 | 129 |  |  |  |
| 38 | 166 |  | 38 | 178 |  |  |  |
| 39 | 151 |  | 39 | 144 |  |  |  |
| 40 | 157 |  | 40 | 150 |  |  |  |
| 41 | 143 |  | 41 | 137 |  |  |  |
| 42 | 127 |  | 42 | 52 |  |  |  |
| 43 | 120 |  | Total | 7163898 |  |  |  |
| 44 | 128 |  |  |  |  |  |  |
| 45 | 94 |  |  |  |  |  |  |
| 46 | 101 |  |  |  |  |  |  |
| 47 | 126 |  |  |  |  |  |  |
| 48 | 114 |  |  |  |  |  |  |
| 49 | 84 |  |  |  |  |  |  |
| 50 | 105 |  |  |  |  |  |  |
| 51 | 87 |  |  |  |  |  |  |
| 52 | 123 |  |  |  |  |  |  |
| 53 | 75 |  |  |  |  |  |  |
| 54 | 99 |  |  |  |  |  |  |
| 55 | 80 |  |  |  |  |  |  |
| 56 | 48 |  |  |  |  |  |  |
| Total |  |  |  |  |  |  |  |

The number of SNPs in LD have been calculated between populations within continent for a) Africa; b) Europe, and c) West Asia. The first and second column from the left respectively indicate the number of populations in which a specific pair of SNPs was found in linkage and the corresponding percentage calculated over the total number of populations from that continent.
