# Supplementary material for: Genome-wide SNP profiling of worldwide goat populations reveals strong partitioning of diversity and highlights post-domestication migration routes
Source: Genet Sel Evol. 2018 Nov 19;50:58. doi: 10.1186/s12711-018-0422-x (PMC6240949; doi:10.1186/s12711-018-0422-x)
Supplement: Supplementary file 8 — Additional file 8. Single-generation plots of Ne values calculated with SNeP software. The single panels correspond to effective population size values, Ne, estimated for a number of generations before present varying between 13 and 959. Breed circles are coloured according to the continent of provenance: blue = west Asia, green = Europe, red = Africa, light blue = South America, orange = Oceania. [file 12711_2018_422_MOESM8_ESM.pdf]

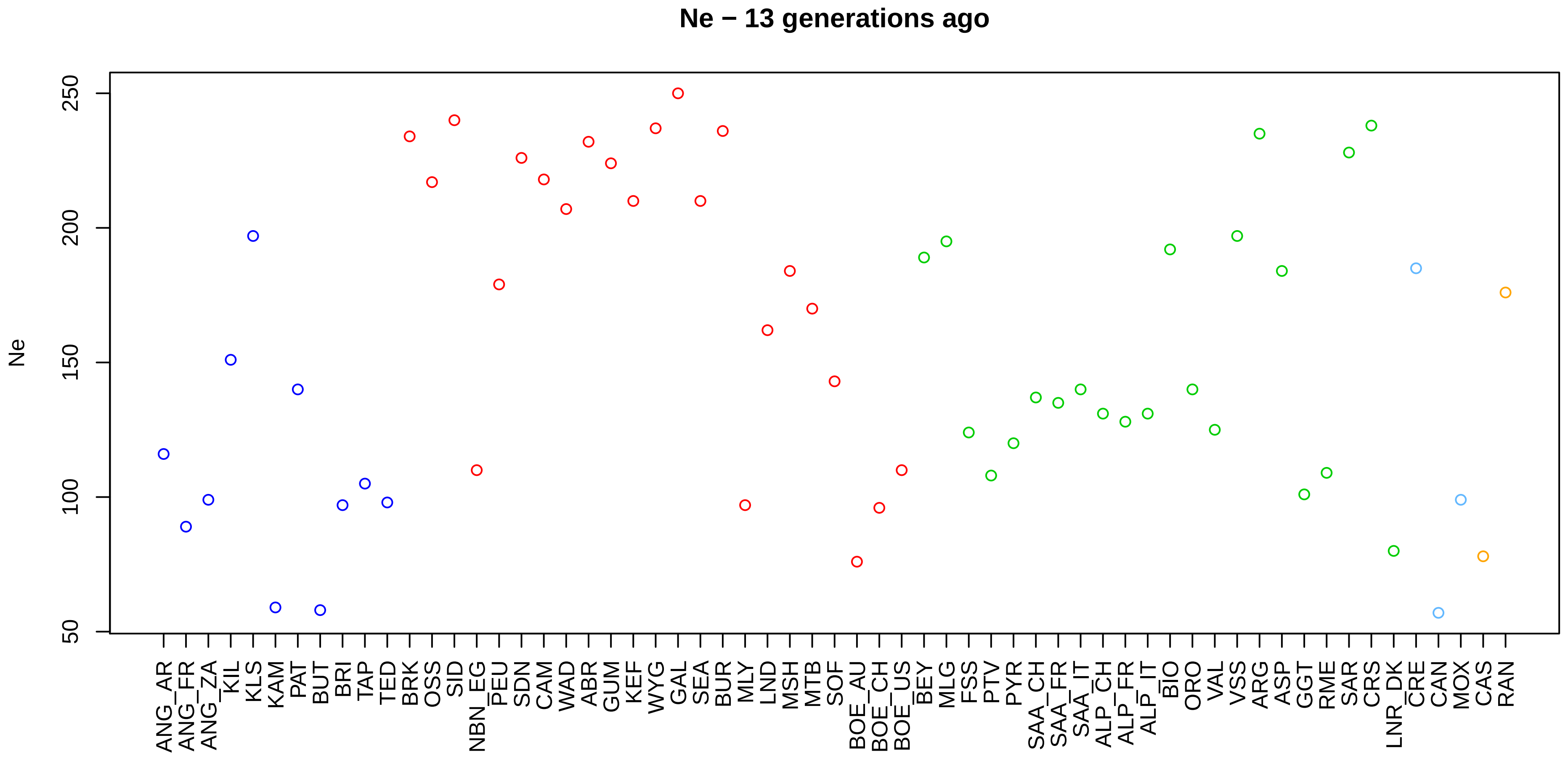

Ne – 13 generations ago
Ne
50
100
150
200
250
ANG_AR ANG_FR ANG_ZA KIL KLS KAM PAT BUT BRI TAP TED BRK OSS SID NBN_EG PEU SDN CAM WAD ABR GUM KEF WYG GAL SEA BUR MLY LND MSH MTB SOF BOE_AU BOE_CH BOE_US BEY MLG FSS PTV PYR SAA_CH SAA_FR SAA_IT ALP_CH ALP_FR ALP_IT BIO ORO VAL VSS ARG ASP GGT RME SAR CRS LNR_DK CRE CAN MOX CAS RAN

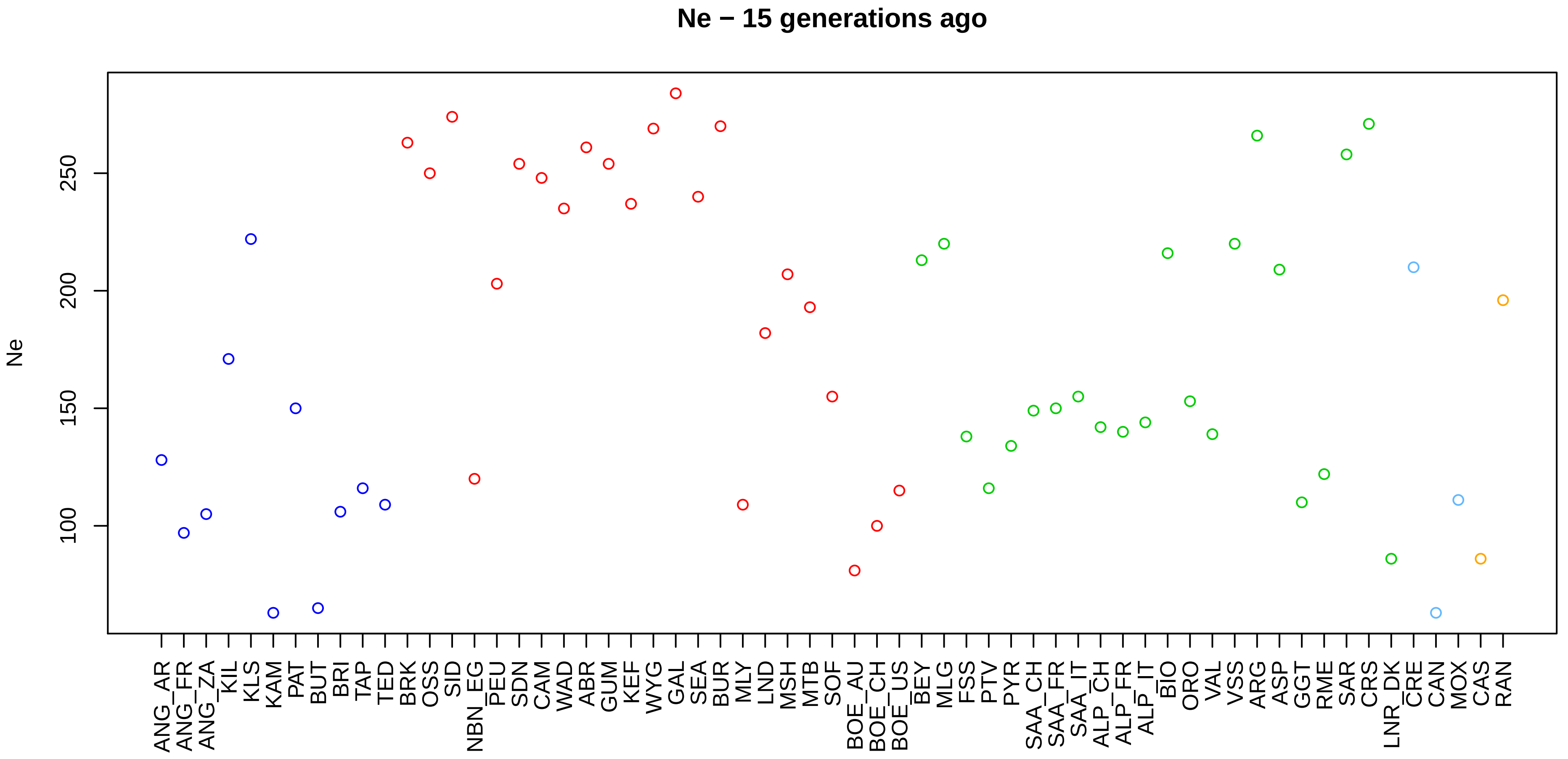

Ne – 15 generations ago
Ne
100
150
200
250
ANG_AR ANG_FR ANG_ZA KIL KLS KAM PAT BUT BRI TAP TED BRK OSS SID NBN_EG PEU SDN CAM WAD ABR GUM KEF WYG GAL SEA BUR MLY LND MSH MTB SOF BOE_AU BOE_CH BOE_US BEY MLG FSS PTV PYR SAA_CH SAA_FR SAA_IT ALP_CH ALP_FR ALP_IT BIO ORO VAL VSS ARG ASP GGT RME SAR CRS LNR_DK CRE CAN MOX CAS RAN

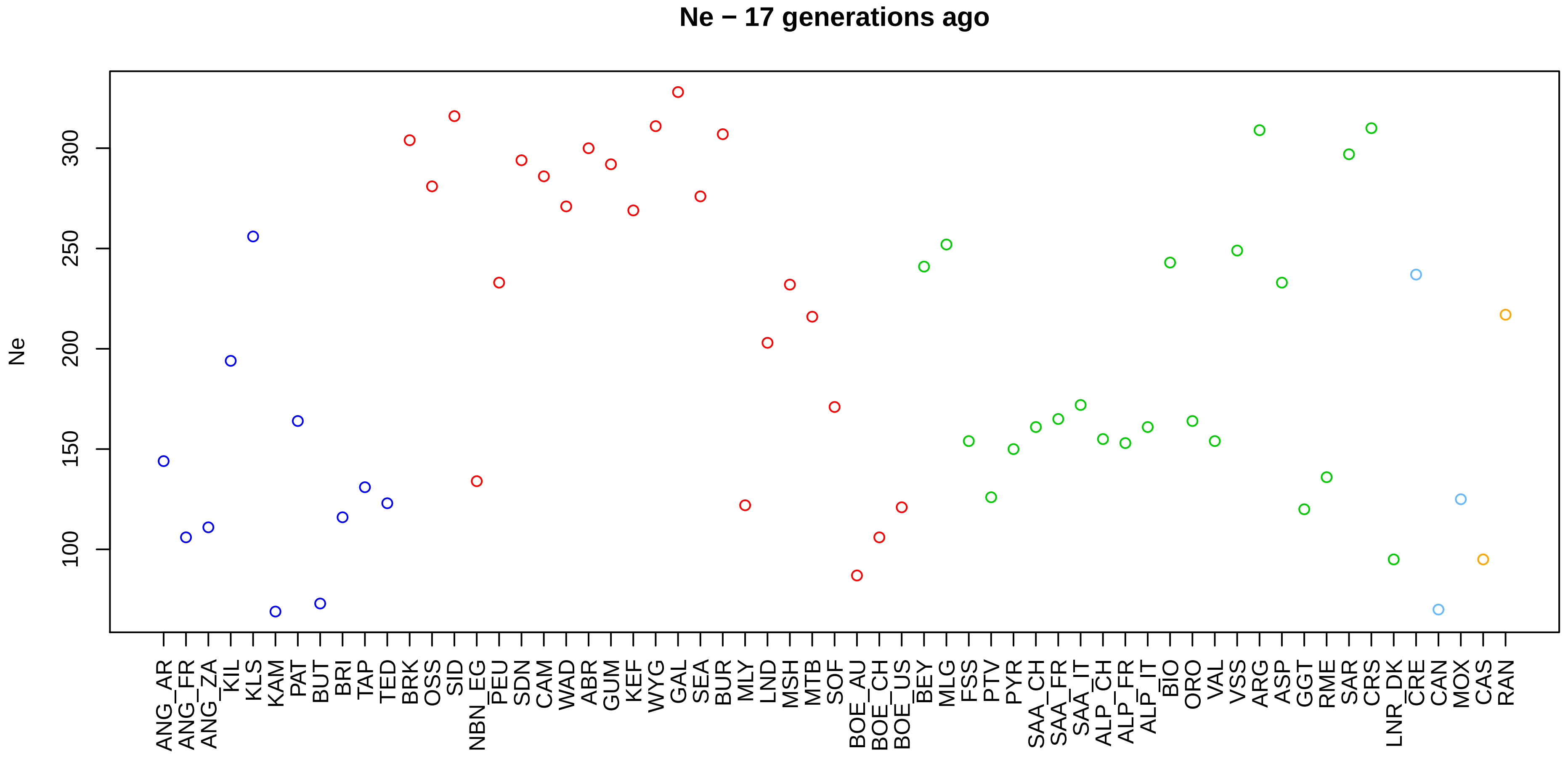

Ne – 17 generations ago
Ne
100
150
200
250
300
ANG_AR ANG_FR ANG_ZA KIL KLS KAM PAT BUT BRI TAP TED BRK OSS SID NBN_EG PEU SDN CAM WAD ABR GUM KEF WYG GAL SEA BUR MLY LND MSH MTB SOF BOE_AU BOE_CH BOE_US BEY MLG FSS PTV PYR SAA_CH SAA_FR SAA_IT ALP_CH ALP_FR ALP_IT BIO ORO VAL VSS ARG ASP GGT RME SAR CRS LNR_DK CRE CAN MOX CAS RAN

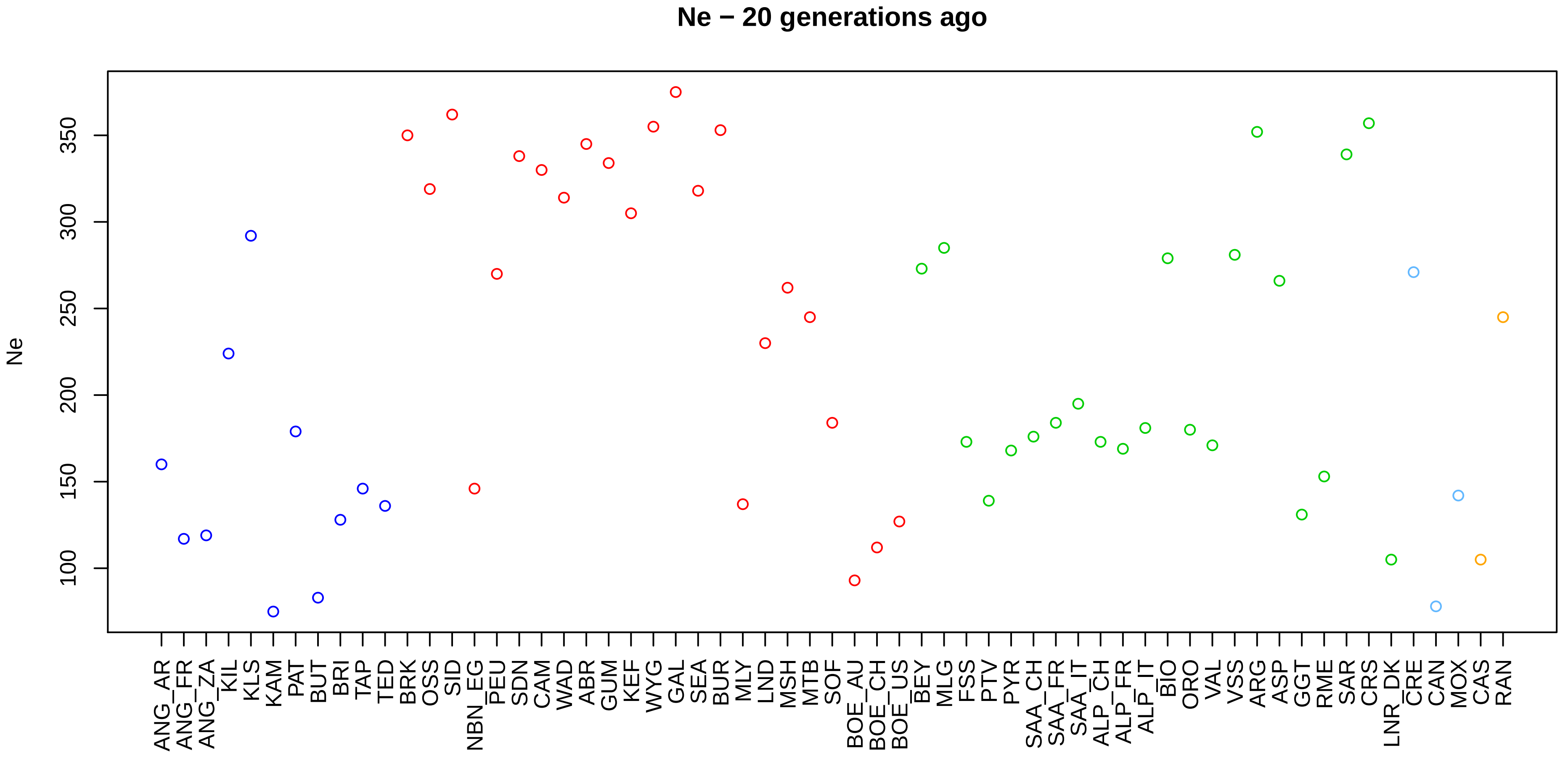

Ne – 20 generations ago
Ne
100
150
200
250
300
350
ANG_AR ANG_FR ANG_ZA KIL KLS KAM PAT BUT BRI TAP TED BRK OSS SID NBN_EG PEU SDN CAM WAD ABR GUM KEF WYG GAL SEA BUR MLY LND MSH MTB SOF BOE_AU BOE_CH BOE_US BEY MLG FSS PTV PYR SAA_CH SAA_FR SAA_IT ALP_CH ALP_FR ALP_IT BIO ORO VAL VSS ARG ASP GGT RME SAR CRS LNR_DK CRE CAN MOX CAS RAN

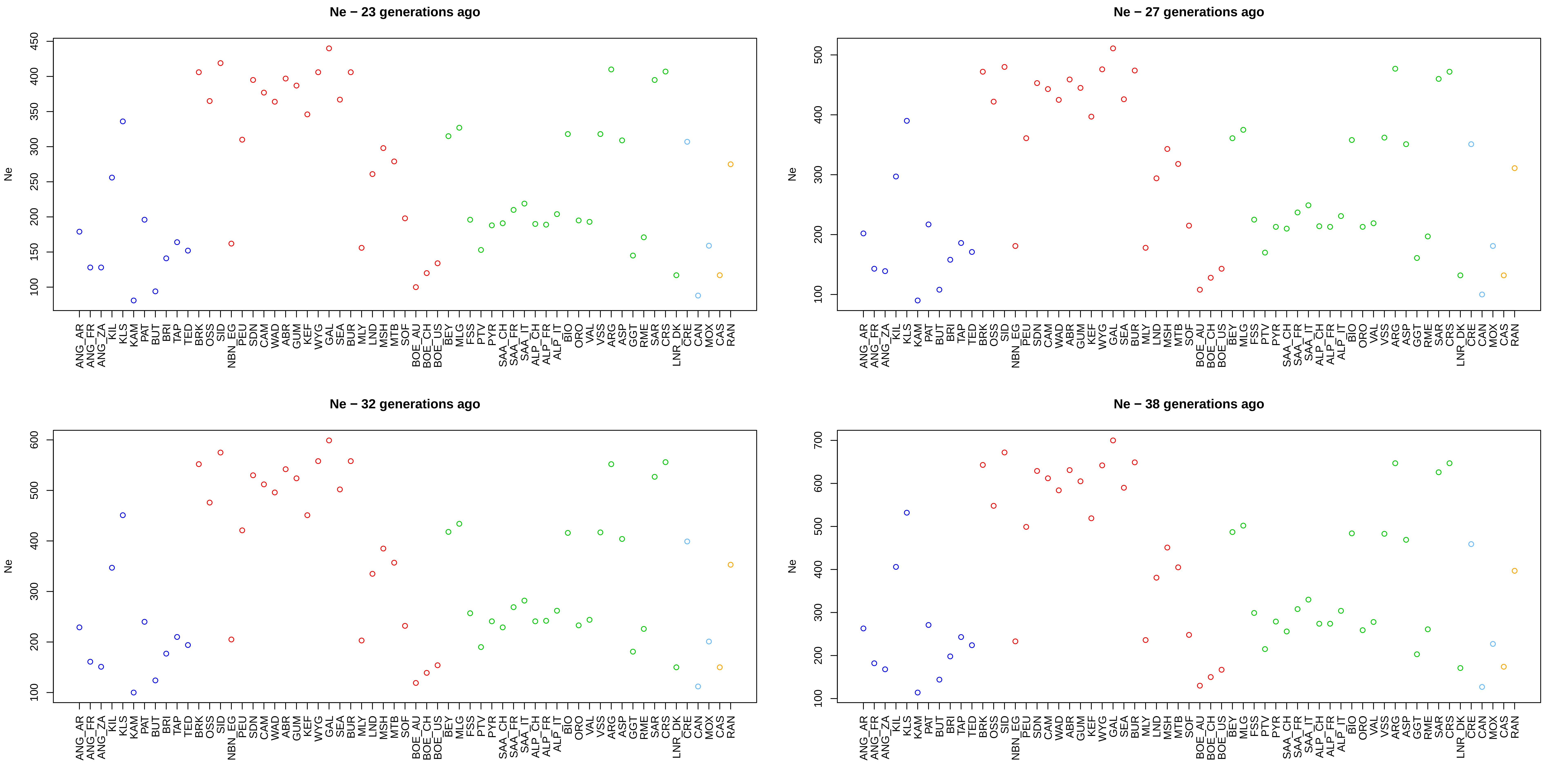
Ne − 23 generations ago
Ne
100 150 200 250 300 350 400 450
ANG_AR ANG_FR ANG_ZA KIL KLS KAM PAT BUT BRI TAP TED BRK OSS SID NBN_EG PEU SDN CAM WAD ABR GUM KEF WYG GAL SEA BUR MLY LND MSH MTB SOF BOE_AU BOE_CH BOE_US BEY MLG FSS PTV PYR SAA_CH SAA_FR SAA_IT ALP_CH ALP_FR ALP_IT BIO ORO VAL VSS ARG ASP GGT RME SAR CRS LNR_DK CRE CAN MOX CAS RAN
Ne − 27 generations ago
Ne
100 200 300 400 500
ANG_AR ANG_FR ANG_ZA KIL KLS KAM PAT BUT BRI TAP TED BRK OSS SID NBN_EG PEU SDN CAM WAD ABR GUM KEF WYG GAL SEA BUR MLY LND MSH MTB SOF BOE_AU BOE_CH BOE_US BEY MLG FSS PTV PYR SAA_CH SAA_FR SAA_IT ALP_CH ALP_FR ALP_IT BIO ORO VAL VSS ARG ASP GGT RME SAR CRS LNR_DK CRE CAN MOX CAS RAN
Ne − 32 generations ago
Ne
100 200 300 400 500 600
ANG_AR ANG_FR ANG_ZA KIL KLS KAM PAT BUT BRI TAP TED BRK OSS SID NBN_EG PEU SDN CAM WAD ABR GUM KEF WYG GAL SEA BUR MLY LND MSH MTB SOF BOE_AU BOE_CH BOE_US BEY MLG FSS PTV PYR SAA_CH SAA_FR SAA_IT ALP_CH ALP_FR ALP_IT BIO ORO VAL VSS ARG ASP GGT RME SAR CRS LNR_DK CRE CAN MOX CAS RAN
Ne − 38 generations ago
Ne
100 200 300 400 500 600 700
ANG_AR ANG_FR ANG_ZA KIL KLS KAM PAT BUT BRI TAP TED BRK OSS SID NBN_EG PEU SDN CAM WAD ABR GUM KEF WYG GAL SEA BUR MLY LND MSH MTB SOF BOE_AU BOE_CH BOE_US BEY MLG FSS PTV PYR SAA_CH SAA_FR SAA_IT ALP_CH ALP_FR ALP_IT BIO ORO VAL VSS ARG ASP GGT RME SAR CRS LNR_DK CRE CAN MOX CAS RAN

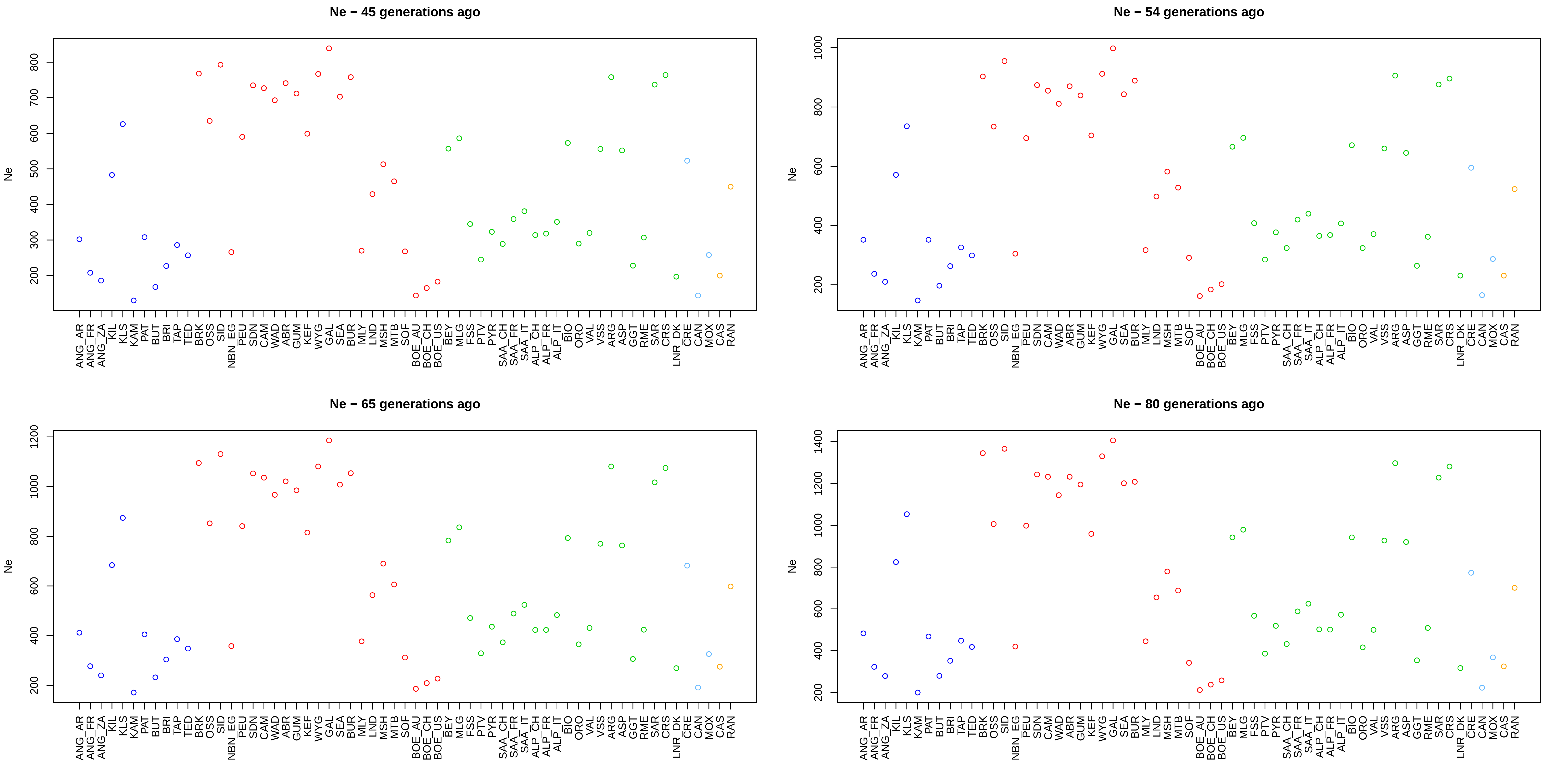

Ne − 45 generations ago
Ne − 54 generations ago
Ne − 65 generations ago
Ne − 80 generations ago
Ne
200 300 400 500 600 700 800
200 400 600 800 1000
200 400 600 800 1000 1200
200 400 600 800 1000 1200 1400
ANG_AR ANG_FR ANG_ZA KIL KLS KAM PAT BUT BRI TAP TED BRK OSS SID NBN_EG PEU SDN CAM WAD ABR GUM KEF WYG GAL SEA BUR MLY LND MSH MTB SOF BOE_AU BOE_CH BOE_US BEY MLG FSS PTV PYR SAA_CH SAA_FR SAA_IT ALP_CH ALP_FR ALP_IT BIO ORO VAL VSS ARG ASP GGT RME SAR CRS LNR_DK CRE CAN MOX CAS RAN

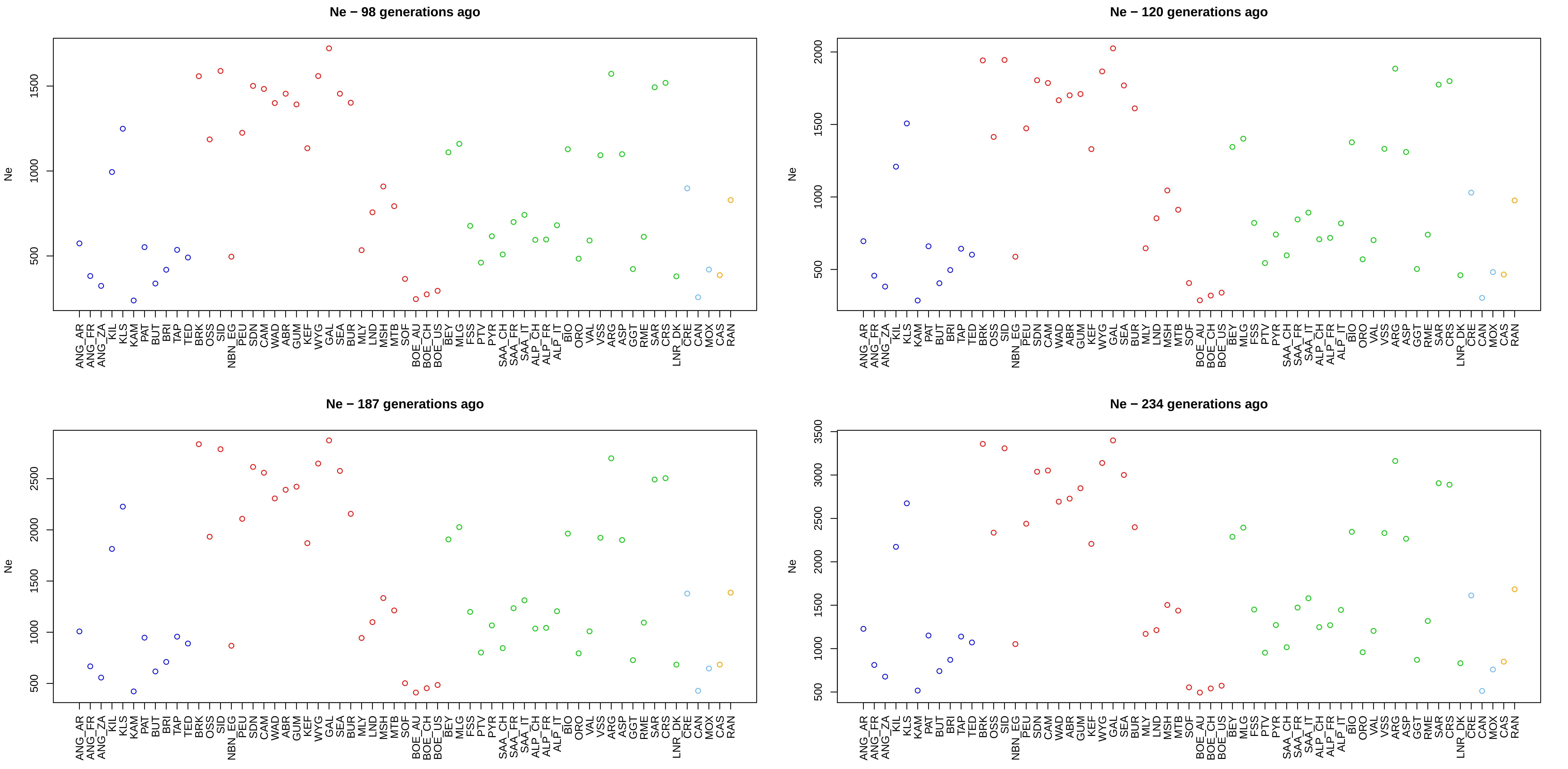

Ne – 98 generations ago
Ne
500
1000
1500
Ne – 120 generations ago
Ne
500
1000
1500
2000
Ne – 187 generations ago
Ne
500
1000
1500
2000
2500
Ne – 234 generations ago
Ne
500
1000
1500
2000
2500
3000
3500
ANG_AR ANG_FR ANG_ZA KIL KLS KAM PAT BUT BRI TAP TED BRK OSS SID NBN_EG PEU SDN CAM WAD ABR GUM KEF WYG GAL SEA BUR MLY LND MSH MTB SOF BOE_AU BOE_CH BOE_US BEY MLG FSS PTV PYR SAA_CH SAA_FR SAA_IT ALP_CH ALP_FR ALP_IT BIO ORO VAL VSS ARG ASP GGT RME SAR CRS LNR_DK CRE CAN MOX CAS RAN

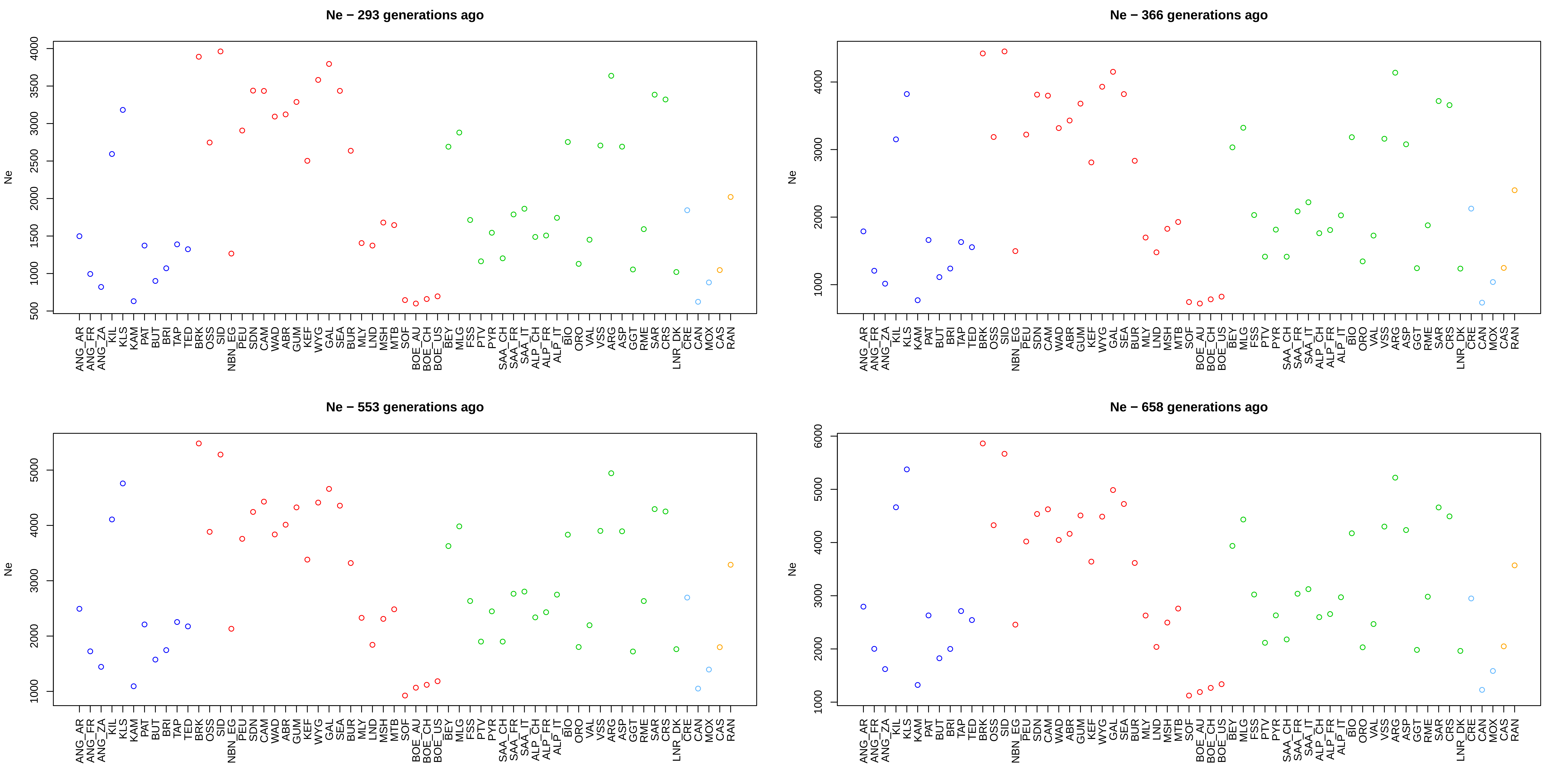
Ne – 293 generations ago
Ne
500 1000 1500 2000 2500 3000 3500 4000
Ne – 366 generations ago
Ne
1000 2000 3000 4000
Ne – 553 generations ago
Ne
1000 2000 3000 4000 5000
Ne – 658 generations ago
Ne
1000 2000 3000 4000 5000 6000
ANG_AR ANG_FR ANG_ZA KIL KLS KAM PAT BUT BRI TAP TED BRK OSS SID NBN_EG PEU SDN CAM WAD ABR GUM KEF WYG GAL SEA BUR MLY LND MSH MTB SOF BOE_AU BOE_CH BOE_US BEY MLG FSS PTV PYR SAA_CH SAA_FR SAA_IT ALP_CH ALP_FR ALP_IT BIO ORO VAL VSS ARG ASP GGT RME SAR CRS LNR_DK CRE CAN MOX CAS RAN

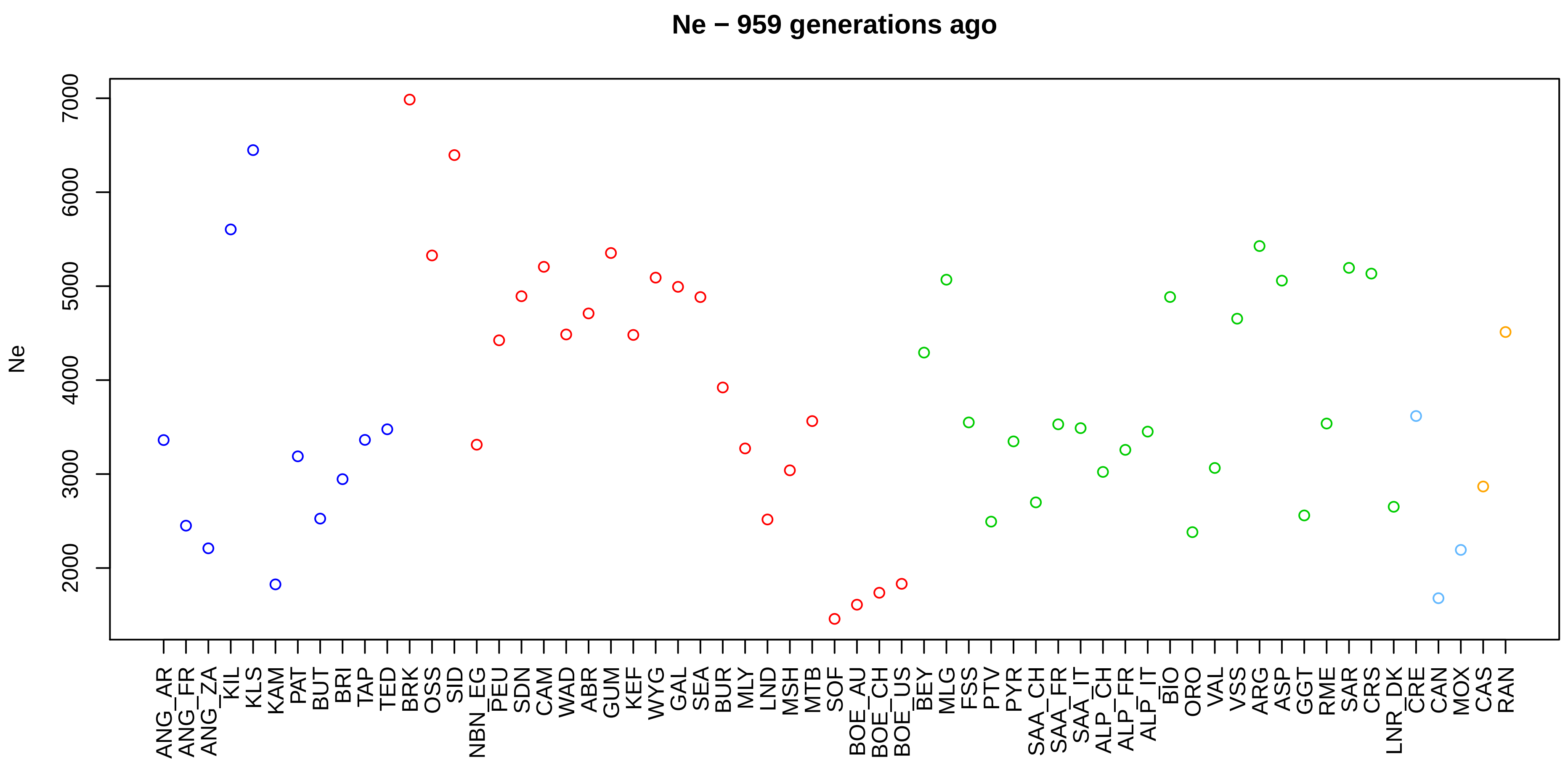
Ne − 959 generations ago
Ne
2000
3000
4000
5000
6000
7000
ANG_AR
ANG_FR
ANG_ZA
KIL
KLS
KAM
PAT
BUT
BRI
TAP
TED
BRK
OSS
SID
NBN_EG
PEU
SDN
CAM
WAD
ABR
GUM
KEF
WYG
GAL
SEA
BUR
MLY
LND
MSH
MTB
SOF
BOE_AU
BOE_CH
BOE_US
BEY
MLG
FSS
PTV
PYR
SAA_CH
SAA_FR
SAA_IT
ALP_CH
ALP_FR
ALP_IT
BIO
ORO
VAL
VSS
ARG
ASP
GGT
RME
SAR
CRS
LNR_DK
CRE
CAN
MOX
CAS
RAN
