# Supplementary material for: Genome-wide SNP profiling of worldwide goat populations reveals strong partitioning of diversity and highlights post-domestication migration routes
Source: Genet Sel Evol. 2018 Nov 19;50:58. doi: 10.1186/s12711-018-0422-x (PMC6240949; doi:10.1186/s12711-018-0422-x)

Geographical distribution of the clusters highlighted by Chromopainter software at K=2.

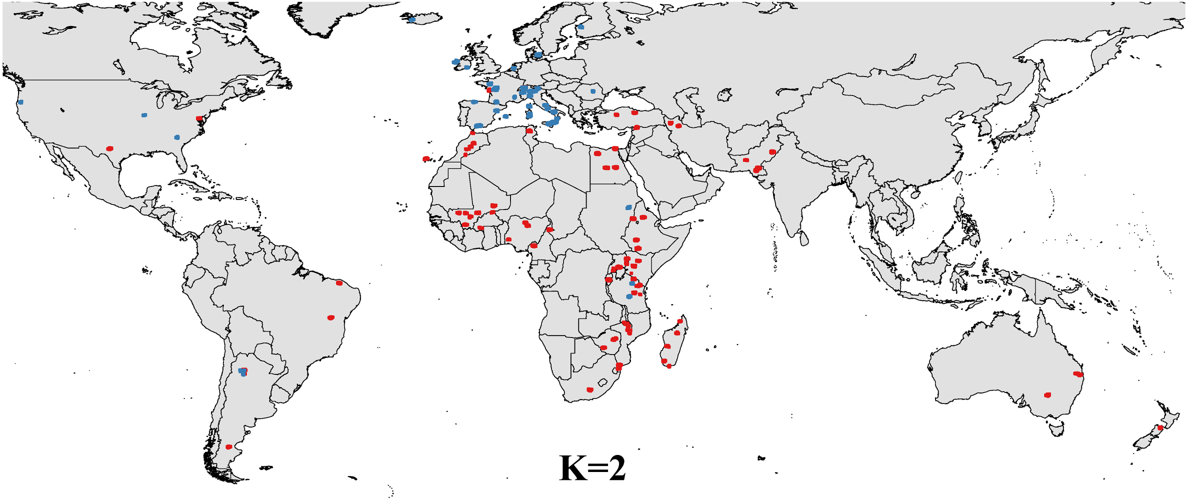

Geographical distribution of the clusters highlighted by Chromopainter software at K=3.

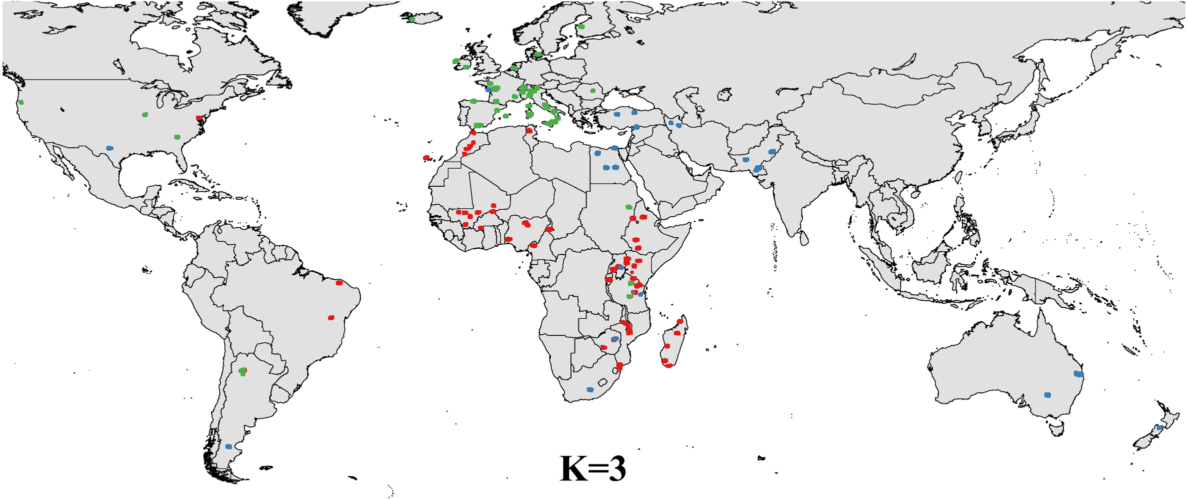

Geographical distribution of the clusters highlighted by Chromopainter software at K=4.

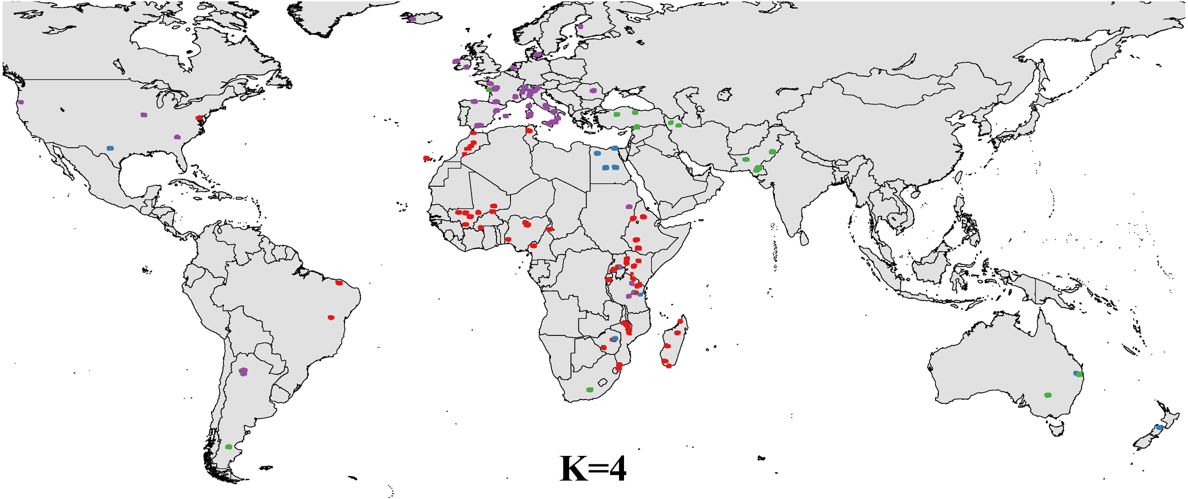

Geographical distribution of the clusters highlighted by Chromopainter software at K=5.

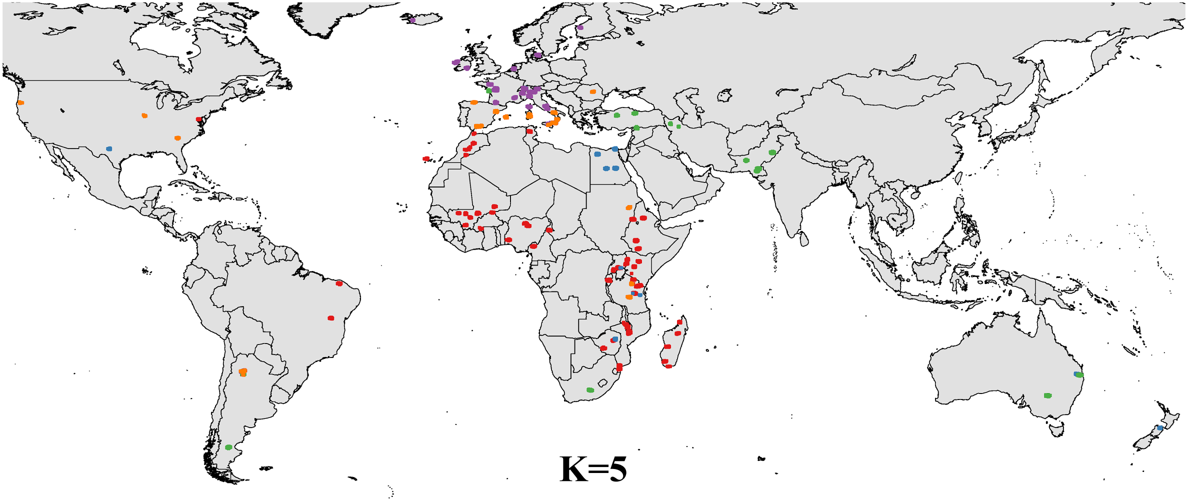

Geographical distribution of the clusters highlighted by Chromopainter software at K=6.

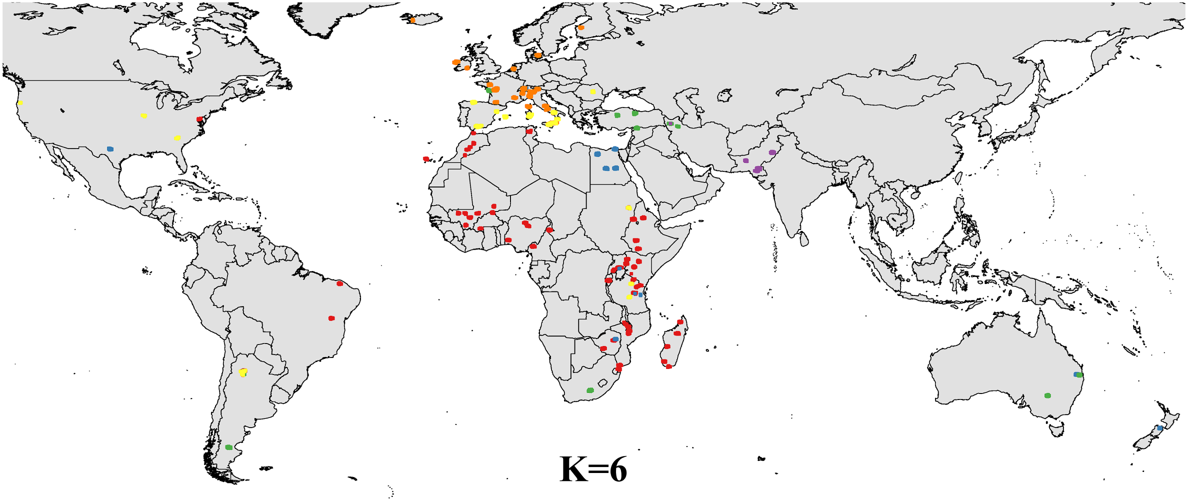

Geographical distribution of the clusters highlighted by Chromopainter software at K=7.

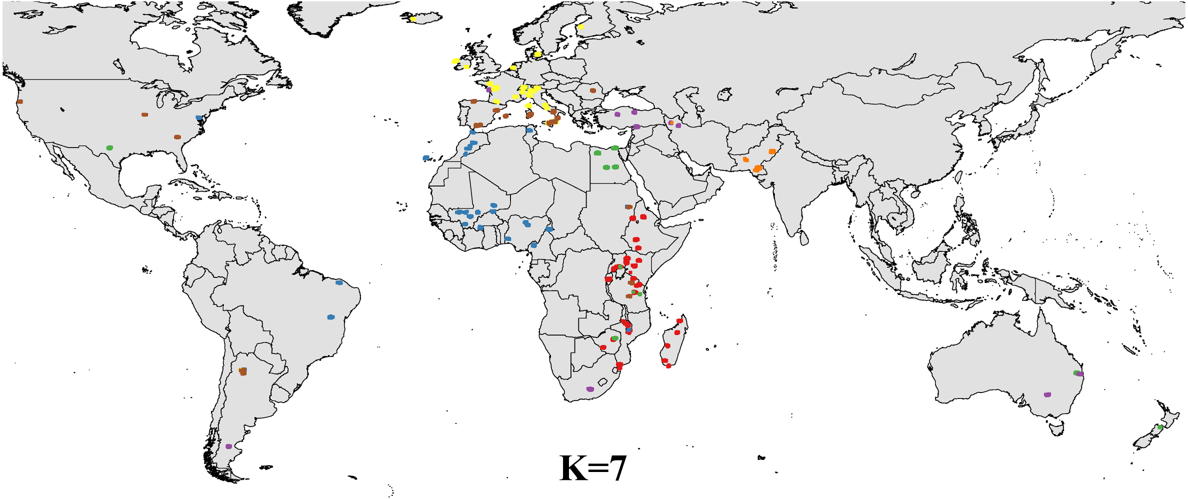

Geographical distribution of the clusters highlighted by Chromopainter software at K=8.

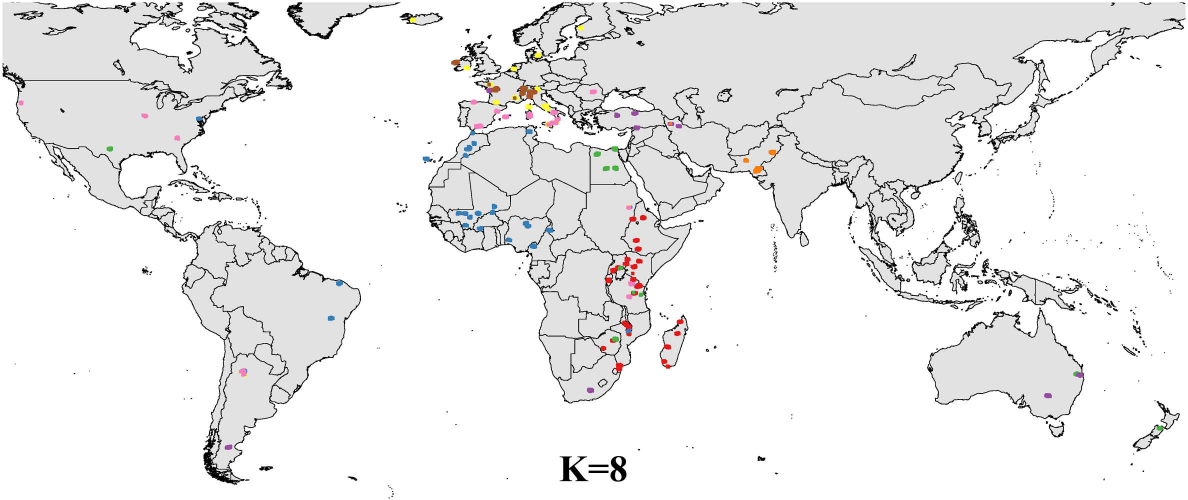

Geographical distribution of the clusters highlighted by Chromopainter software at K=9.

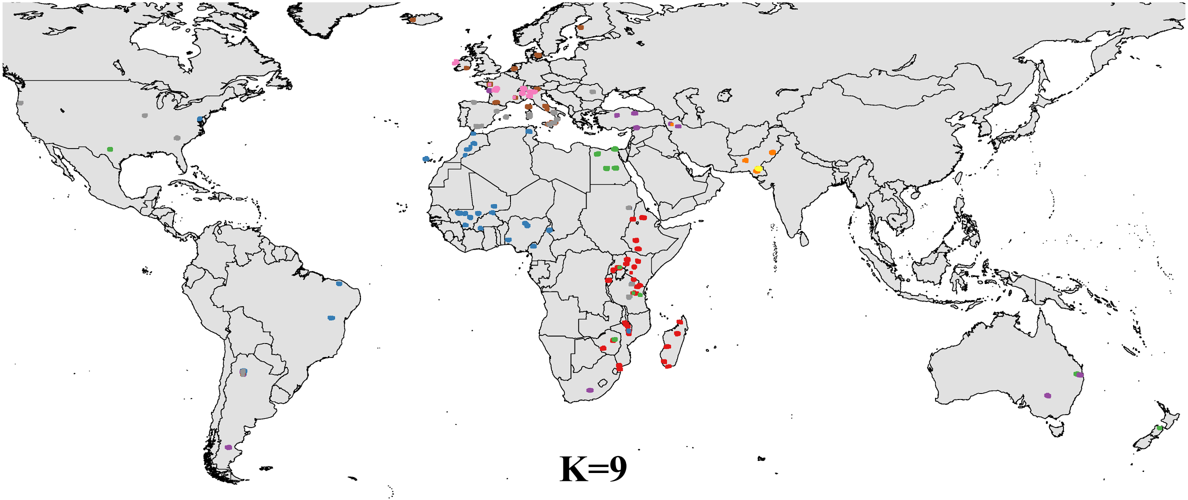

Geographical distribution of the clusters highlighted by Chromopainter software at K=10.

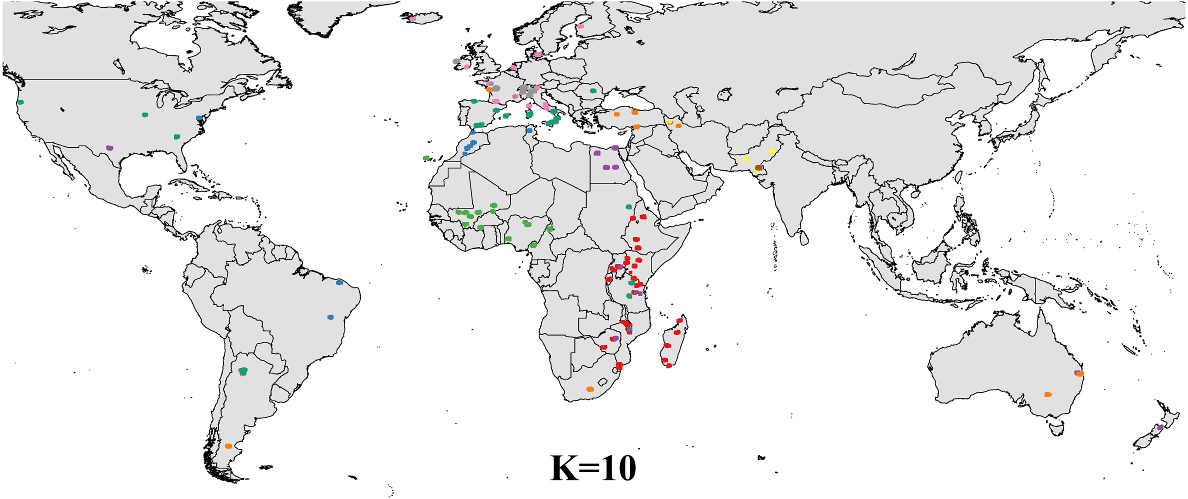

Geographical distribution of the clusters highlighted by Chromopainter software at K=11.

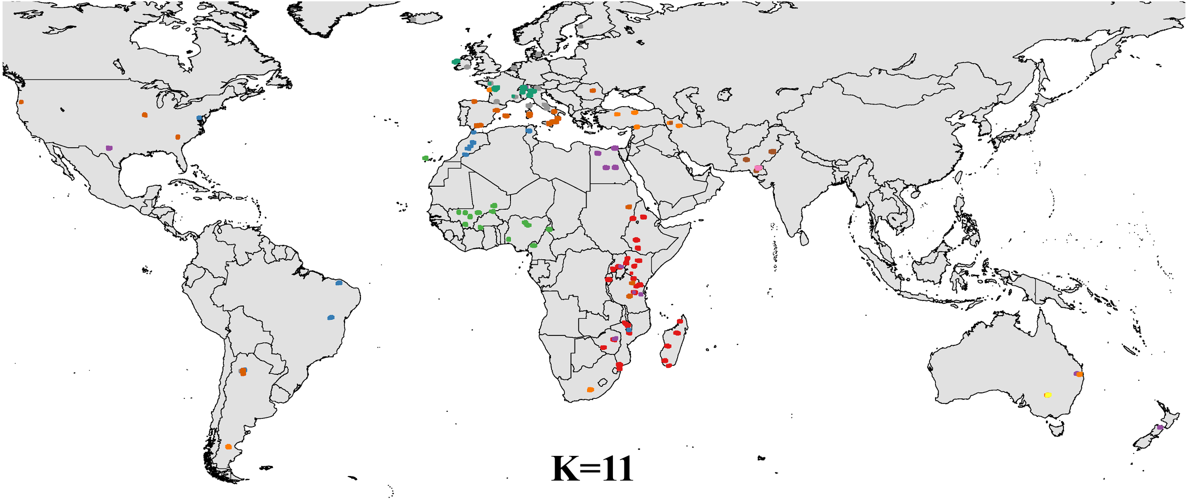

Geographical distribution of the clusters highlighted by Chromopainter software at K=12.

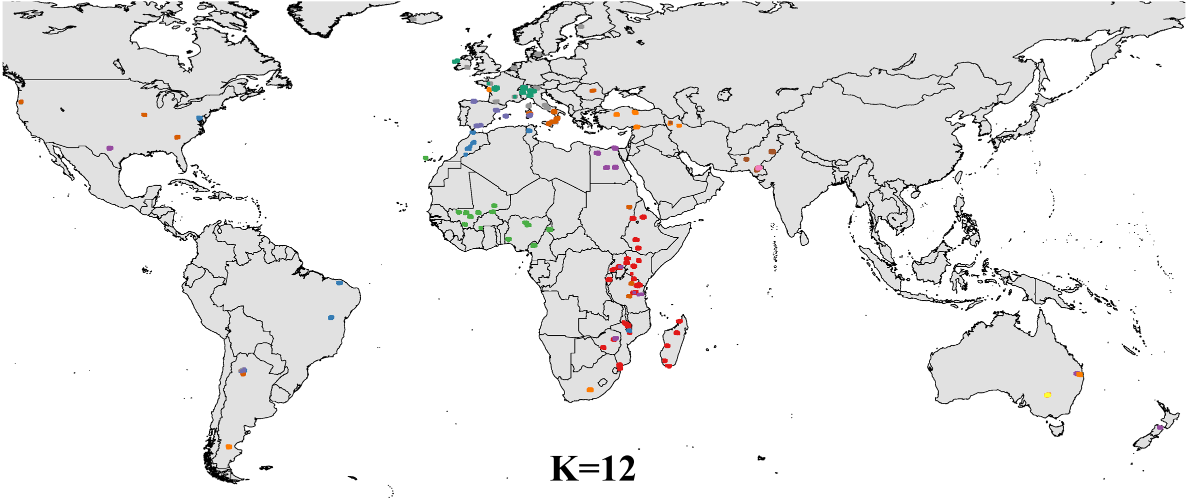

Geographical distribution of the clusters highlighted by Chromopainter software at K=13.

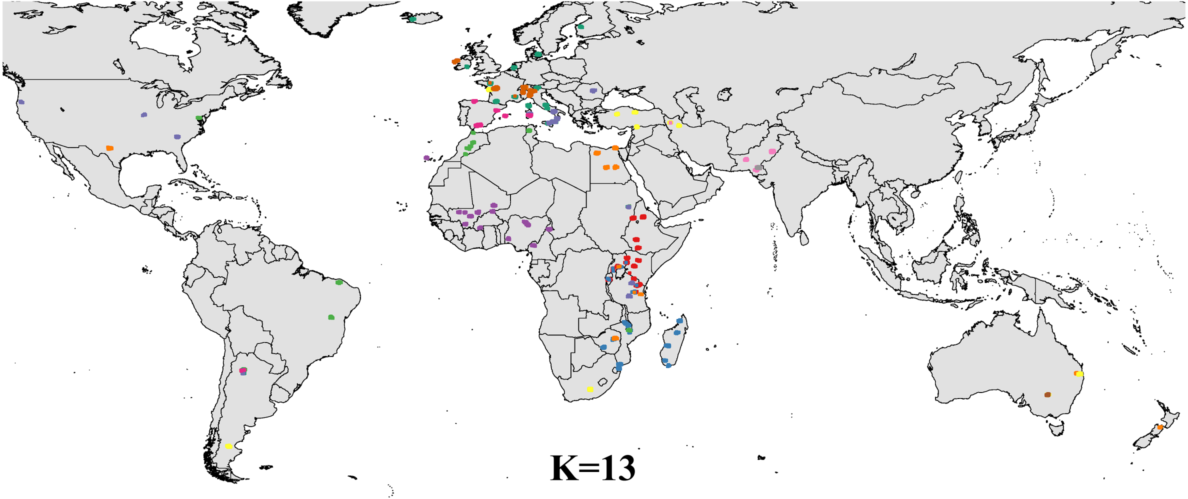

Geographical distribution of the clusters highlighted by Chromopainter software at K=14.

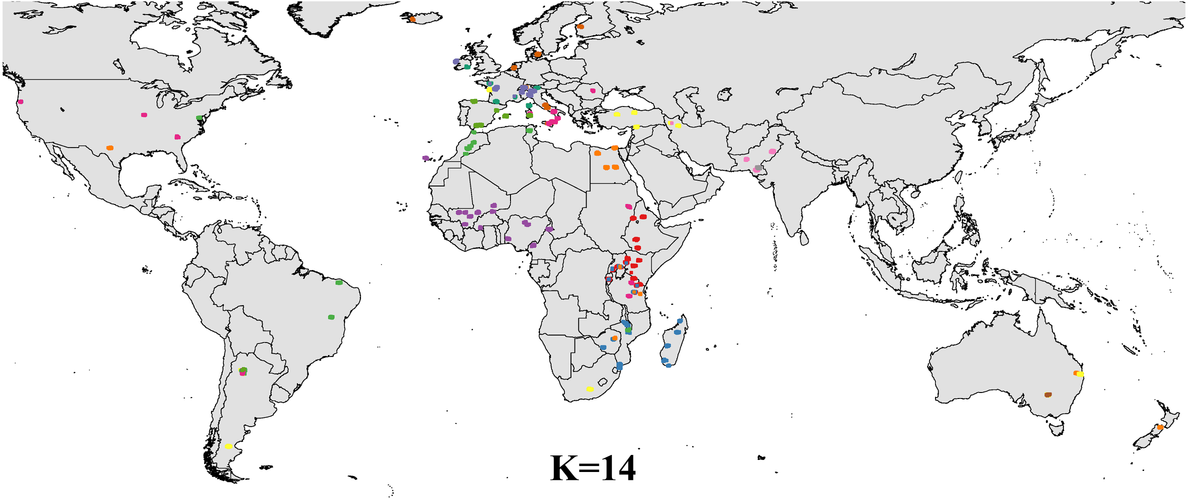

Geographical distribution of the clusters highlighted by Chromopainter software at K=20.

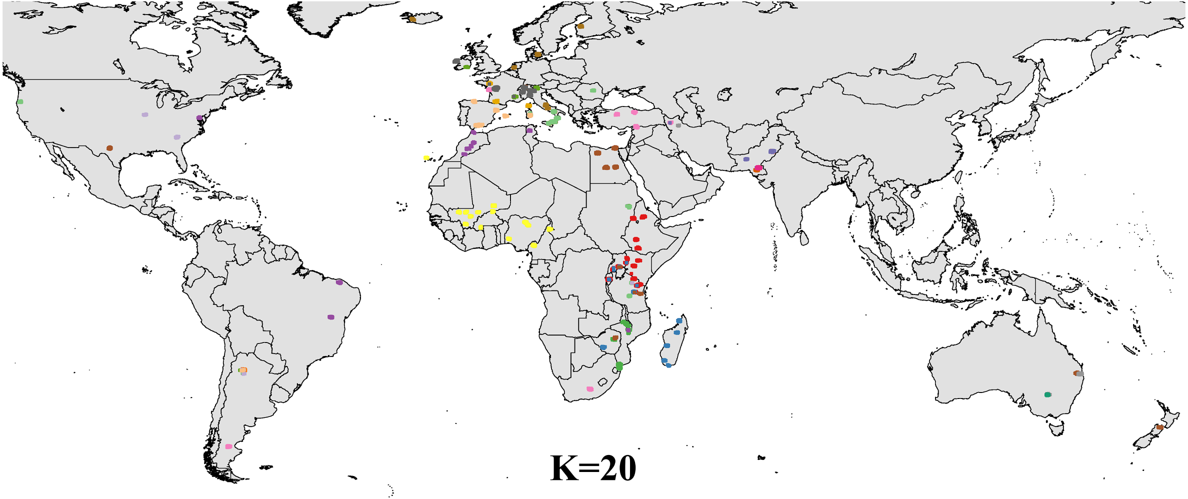

Geographical distribution of the clusters highlighted by Chromopainter software at K=50.

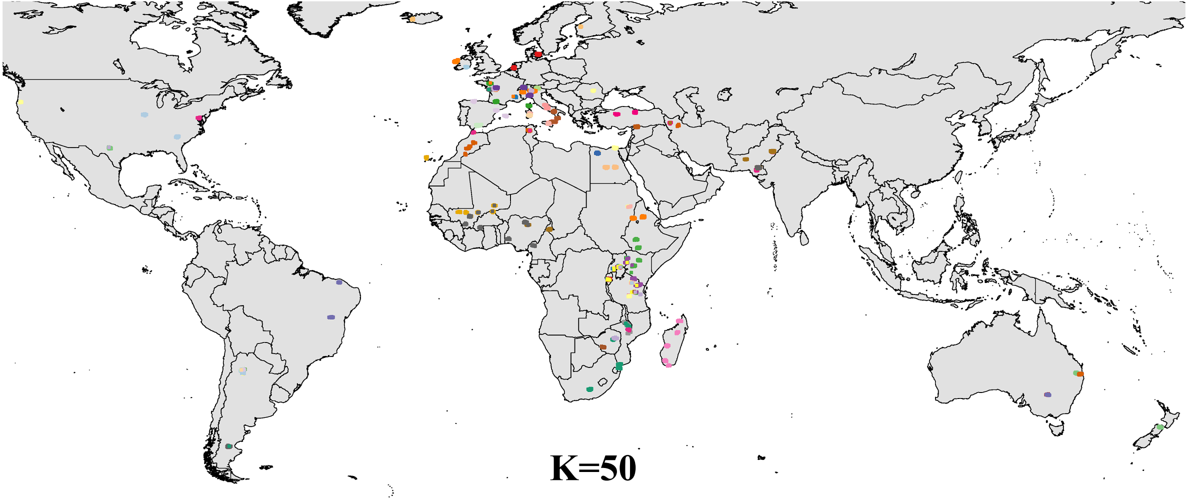

Supplement: Supplementary file 14 — Additional file 14. Geographical distribution pattern of Chromopainter clustering. Results are shown for clustering solutions for K values from 2 to 14, K = 20 and K = 50. [file 12711_2018_422_MOESM14_ESM.pdf]
